# Whole‐genome duplication promotes phenotypic and genetic adaptation to elevation

**DOI:** 10.1111/nph.71572

**Published:** 2026-09-21

**Authors:** Sandra Grünig, Marc Beringer, Christian Parisod

**Affiliations:** ^1^ Department of Biology University of Fribourg 1700 Fribourg Switzerland; ^2^ Institute of Plant Sciences University of Bern 3013 Bern Switzerland

**Keywords:** adaptation, autopolyploidy, *Biscutella laevigata*, elevation gradient, transplant experiment, whole‐genome sequencing

## Abstract

- Whole‐genome duplication (WGD) is often considered as either an adaptive dead‐end or a facilitator of rapid adaptation under changing environments. How diploids vs tetraploids out of WGD respond to selection remains, however, poorly understood.
- Using whole‐genome sequence data and phenotypic evidence from transplant experiments of diploid and autotetraploid populations of *Biscutella laevigata* (Brassicaceae) sampled at low and high elevation in a factorial design, we here assessed how genomic and phenotypic variation responded to selection across elevation in each ploidal level.
- While all populations showed similar decay of linkage disequilibrium and synonymous genetic variation, tetraploids showed higher nonsynonymous genetic variation, consistent with relaxed purifying selection following WGD and a higher proportion of candidate loci supportive of adaptation to elevation than diploids. Despite the strong plasticity of most phenotypes in both ploidal levels, diploids maintained high fitness homeostasis across elevation, whereas genetically variable tetraploids exhibited contrasted fitness consistent with adaptation to elevation.
- As compared to diploids of *B. laevigata* that evolved adaptive phenotypic plasticity, genomic variation accumulated in tetraploids promoted a strong response to selection, refuting the prediction that WGD is an adaptive dead‐end and indicating that it promotes post‐WGD adaptation across heterogenous environments.

## Graphical abstract

Fitness of diploids (2*x*) and tetraploids (4*x*) of *Biscutella laevigata* from low (circles) and high (triangles) elevation transplanted across elevation at 585, 1236 and 1967 m above sea level.

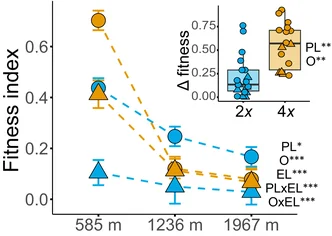

## Introduction

Whole‐genome duplication (WGD) is a prevalent evolutionary process (Wood *et al*., 2009; Jiao *et al*., 2011; Wang *et al*., 2025), often hypothesized to facilitate adaptive radiation during periods of environmental changes (Van de Peer *et al*., 2017). However, autotetraploids out of WGD *per se* (thereafter, tetraploids) have historically been viewed as ‘evolutionary dead‐ends’ (Stebbins, 1971) and their capacity to respond to selection – relative to that of diploids – remains poorly understood (Otto & Whitton, 2000; Soltis & Soltis, 2000; Clo, 2022; Zwaenepoel, 2025). Despite indirect evidence supporting that tetraploids commonly adapt and thrive in their own ecological niche (Meirmans & Kolář, 2025), particularly under harsh conditions (Rice *et al*., 2019), it remains unclear to what extent the distribution of diploids and related tetraploids across ecological gradients is primarily driven by phenotypic plasticity or by genetically based adaptation (Parisod *et al*., 2010). Although ploidal levels exhibited similar changes in traits across different environmental conditions (i.e. reaction norms) when experimentally tested (Petit *et al*., 1996; Bretagnolle & Thompson, 2001; Wei *et al*., 2019), WGD has been postulated to enhance adaptive phenotypic plasticity and increase the fitness homeostasis of tetraploids in heterogenous environments (i.e. tetraploids are ‘jack‐of‐all‐trades’; Richards *et al*., 2006; Wei *et al*., 2019). Understanding how selection shapes genetic and phenotypic adaptation to environmental heterogeneity in diploids vs tetraploids remains a key unresolved question regarding the evolutionary consequences of WGD.

The tetrasomic inheritance resulting from WGD has pervasive effects on the population genetics of tetraploids, slowing allele‐frequency shifts and delaying progression toward homozygosity, thereby reducing neutral genetic differentiation relative to diploids (Ronfort *et al*., 1998; Gallais, 2003). Combined with the greater number of possible conditions of dominance interactions, WGD is predicted to lower variance in fitness and response to selection (Hill, 1971; Ronfort *et al*., 1998), while increased masking of deleterious alleles may exacerbate genetic load during range expansion (Booker & Schrider, 2025). On the contrary, the WGD‐induced increase in effective population size for a given census size may enhance the adaptive sorting of (partially dominant) alleles in tetraploids (Otto & Whitton, 2000; Gallais, 2003). Combined with their ability to accumulate variants through mutation and/or gene flow, this has led to the suggestion that tetraploids may adapt faster than diploids to new environments (Baduel *et al*., 2018; Monnahan & Brandvain, 2020; Konečná *et al*., 2021). Although multilocus models also investigated how WGD may affect polygenic adaptation (Griswold & Williamson, 2017; Mostafaee & Griswold, 2019; Griswold, 2021; Zwaenepoel, 2025; Clo, 2026), predictions remain challenging as tetrasomic inheritance expectedly increases the recombination rate (Sved, 1964) and contrastingly affects linked selection, even in the absence of epistasis.

Several examples of effective tetraploid establishment have been documented (Manton, 1934; Arnold *et al*., 2015; Visger *et al*., 2016), offering valuable empirical systems toward a better understanding of adaptation following WGD. However, scarce studies have characterized the genetic responses of diploids as well as tetraploids to selection. Indirect evidence from genomic variation in the mixed‐ploidy *Arabidopsis arenosa* highlighted an increased load of nonsynonymous variants being confounded with reduced linkage disequilibrium (LD) in tetraploids compared to diploids, suggesting that WGD may interfere with linked selection and local adaptation (Monnahan *et al*., 2019). Although experimental evolution in yeast has shown that tetraploids adapt significantly faster to novel environments than diploids, the impact of selection and mutational input following WGD remains to be disentangled (Selmecki *et al*., 2015). Despite accumulating evidence that refute WGD as an adaptive dead‐end, underpinnings of adaptation to the environment require further investigation across mixed‐ploidy systems.

To compare the response of diploids vs tetraploids to selection, it is crucial to assess their genetic and phenotypic makeup across similar environmental pressures. Elevation gradients, associated with considerable abiotic and biotic changes over limited spatial extents, offer ideal opportunities to investigate responses to divergent selection and how organisms adapt to environmental heterogeneity (Kawecki & Ebert, 2004). In particular, higher elevation is typically associated with reduced temperature and partial CO_2_ and O_2_ pressures, shorter growing season, increased variability in climatic conditions, and increased exposure to ultra‐violet light (Körner, 2021). Many species accordingly demonstrated genetically based differentiation of developmental and physiological traits linked to such climatic differences, with genotypes from high elevation generally producing shorter plants growing slower with thicker leaves and supporting increased tolerance to frost as compared to genotypes from lower elevation (Halbritter *et al*., 2018). Although broad generalization is still premature (Moreira *et al*., 2018), herbivore pressure is expectedly decreased at high elevation (Galmán *et al*., 2018) so that adaptive variation related to both mutualistic and antagonistic biotic interactions can be expected (Knauer *et al*., 2018).

Genetic and phenotypic adaptation to the environment was here investigated using the long‐lived, strictly outcrossing *Biscutella laevigata* L. (Brassicaceae) species complex, in which WGD of specific diploids around the last glacial maximum gave rise to tetraploids displaying tetrasomic inheritance (Grünig *et al*., 2024). Current diploids of *B. laevigata* have demonstrated adaptation to serpentine (Bürki *et al*., 2024) and toxic soils such as mines (Babst‐Kostecka *et al*., 2014). Consistent with a negligible impact of WGD *per se* and a predominant role of post‐WGD evolution in the adaptive evolution of tetraploids (Grünig *et al*., 2026), genetic variation was regularly associated with the environment as expected by local adaptation of tetraploids (Parisod & Christin, 2008; Leempoel *et al*., 2018; Grünig *et al*., 2024). As both diploids and tetraploids of *B. laevigata* occur across large elevation gradients in the European Alps (from *c*. 100 to > 2500 m above sea level (asl)), this mixed‐ploid system offers a unique opportunity to assess how WGD interacts with selection. This study hence characterizes genomic and phenotypic variation among diploid vs tetraploid plants sampled at low vs high elevation in a factorial design, with the overarching aim to compare their response to selection across elevation gradients. More specifically, we here assess (i) genomic variation and LD to assess the impact of purifying vs linked selection in diploid vs tetraploid populations. Then, we estimate how diploids vs tetraploids have adapted to elevation through both (ii) indirect scans of whole‐genome sequence variation identifying loci with signatures of selection and (iii) direct phenotypic evidence following transplantation of a subset of populations across elevation.

## Materials and Methods

### Sampling and genotyping in diploids and tetraploids

Eight individuals in each of 12 populations of diploids and tetraploids of *Biscutella laevigata* L. (Brassicaceae) from low‐ vs high‐elevation environments sampled in a factorial design were sequenced to address genomic signatures of adaptation to elevation (Fig. 1a). All diploid populations reported at high elevation and most tetraploid populations known from low elevation were included (Grünig *et al*., 2024).

**Fig. 1:**
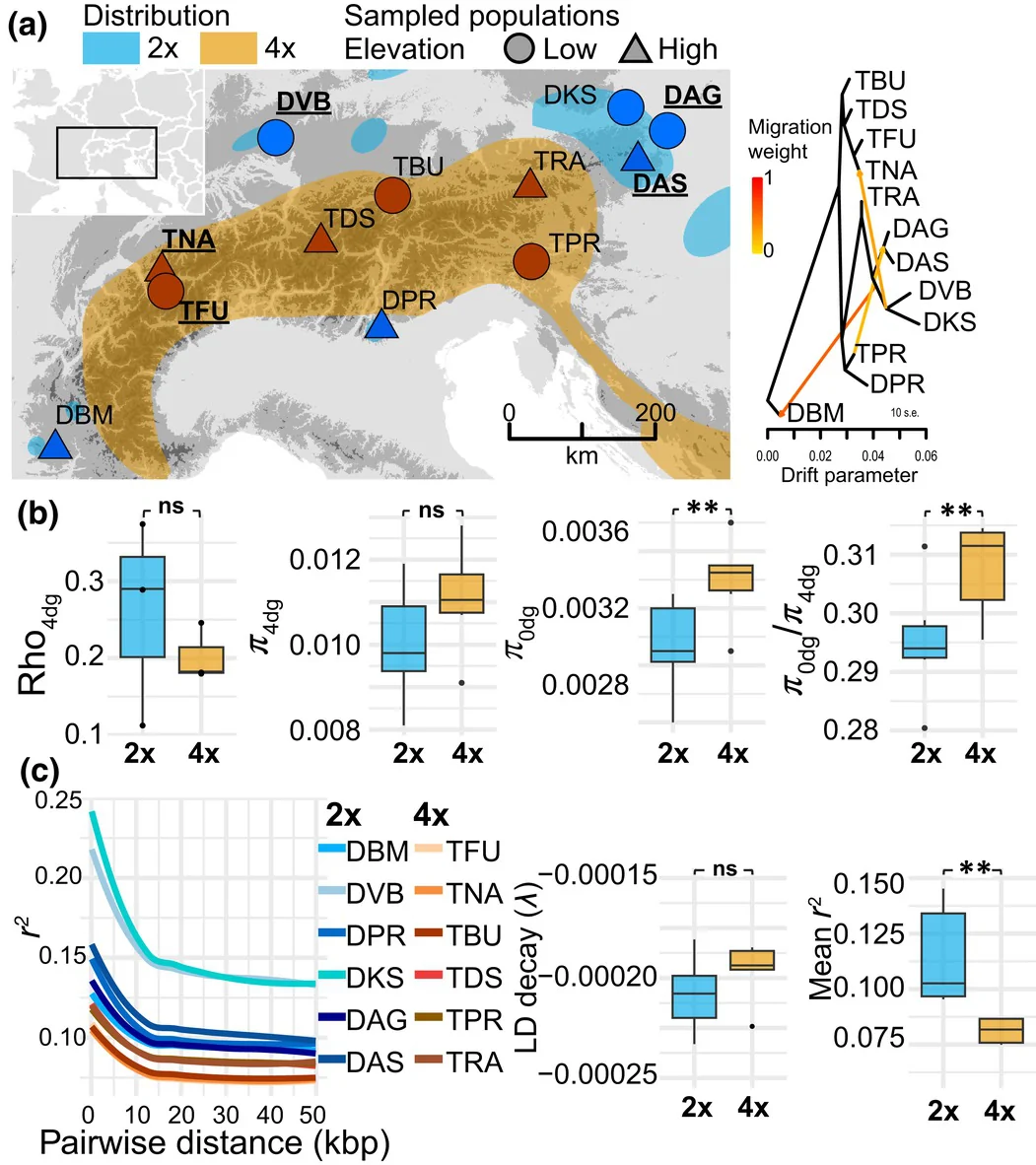
Distribution of genomic variation in diploid (2*x*) and tetraploid (4*x*) populations of *Biscutella laevigata* from low and high elevation. (a) Distribution of sampled populations across ploidal levels and population tree and migration events inferred in TreeMix. Underlined populations were included in the reciprocal transplant experiment. (b) Pairwise population rho differentiation at 4dg sites (three pairs per ploidy level) and *π* nucleotide diversity at 4dg, 0dg, and the ratio *π*_0dg_/*π*_4dg_ across ploidal levels (six pops per ploidy level). (c) Decay of linkage disequilibrium between pairs of loci (*r* ^2^) as a function of physical distance in field populations and comparison of rates of LD decay (*λ*) and overall mean *r* ^2^ after decay. Boxplots show the median, interquartile range (25th–75th percentiles), and whiskers extending to 1.5 × the range; values beyond the whiskers are shown as individual points. Significance levels: **, *P* < 0.01; ns, *P* ≥ 0.05.

For each individual sample, DNA was extracted from 20 mg of dried leaves using the DNeasy Plant Mini Kit (Qiagen) following the manufacturer's instructions. TruSeq DNA PCR‐Free libraries were prepared and sequenced with Paired‐End 2 × 250 bp cycles on six lanes of the Illumina NovaSeq 6000 at the Next Generation Sequencing platform of the University of Bern (Switzerland). To account for the higher coverage required to accurately genotype autopolyploids, tetraploid samples were sequenced twice as much as diploids (Supporting Information Table S1). Reads were mapped to the genome assembly of the diploid *B. laevigata* (Beringer *et al*., 2024), using the mem algorithm from the Burrows–Wheeler Alignment tool v.0.7.15 (Li & Durbin, 2009) with default settings. PCR duplicates were marked using picard v.2.8 (http://broadinstitute.github.io/picard) and discarded together with reads that showed low mapping quality (< 25). The Genome Analysis Toolkit (GATK) v.3.7 (McKenna *et al*., 2010) was used to call variants and invariant sites using the pipeline available at https://github.com/vlkofly/Fastq‐to‐vcf. For each sample, variants were called through local re‐assembly of haplotypes using the HaplotypeCaller module and calling full genotypes for both diploids and tetraploids. Variants were then aggregated across all samples with the GenotypeGVCFs module.

Biallelic single‐nucleotide polymorphisms (SNPs) were filtered for quality following the GATK hard‐filtering recommendations by removal of SNPs with Quality by Depth (QD) < 2.0, FisherStrand (FS) > 60.0, RMSMappingQuality (MQ) < 40.0, MappingQualityRankSumTest (MQRS) < −12.5, ReadPosRankSum < −8.0, and StrandOddsRatio (SOR) > 3.0. Invariant sites with a QUAL < 15 were excluded from the dataset. Only SNPs among the 13 largest scaffolds assembled into nine pseudo‐chromosomes (87.5% of the total assembly including 96.7% of gene models) were further considered to avoid artefacts from poorly assembled regions. Simple repetitive regions and genomic regions annotated as transposable elements (Beringer *et al*., 2024) were further excluded. Following Konečná *et al*. (2021), potentially paralogous mis‐assembled regions were excluded by removing sites with excess depth, defined as higher than two times the SD, in any of the field populations. Furthermore, we followed Arnold *et al*. (2015) and excluded annotated genes or intergenic segments of 2 kb being constantly genotyped (minimal number of called genotypes = 5) as heterozygous at more than two sites in at least two of the diploid populations. The resulting dataset was subsequently further filtered to fulfil the requirements of each analysis.

### Genetic diversity and linkage disequilibrium within and among field populations

The classification of 4dg and 0dg SNPs was inferred from their position in the *B. laevigata* gene model annotation (Beringer *et al*., 2024) using the Degeneracy pipeline (Kent *et al*., 2025; https://github.com/tvkent/Degeneracy). Genome‐wide metrics of diversity within populations (*π* and *θ*_W_) were estimated using the ScanTools pipeline (https://github.com/pmonnahan/ScanTools) on biallelic SNPs and invariant sites from the initial dataset that were harboring < 30% missing calls after setting minimal genotype depths of 4 for diploids and 8 for tetraploids. To estimate *π*, *θ*_W_, and Tajima's *D*, populations were sampled down to five individuals per site to account for the unequal distribution of missing data among samples, while keeping a maximum number of sites. Differences in genetic diversity *π* and *θ*_W_ between ploidal levels were tested using a Welch's *t*‐test and differences in Tajima's *D* using a Wilcoxon rank test.

Pairwise differentiation among populations was estimated from synonymous 4dg sites using rho (Ronfort *et al*., 1998), an analogue to *F*_ST_ that is independent of the ploidy level and of the rate of double reduction, and the difference of differentiation between ploidal levels was tested using a Welch's *t*‐test. Population tree and migration events were inferred from 4dg sites using TreeMix v.1.13 (Pickrell & Pritchard, 2012). To obtain a fixed topology, we built a consensus population tree from 100 bootstrap replicates, using the western diploid population DBM as the outgroup (Grünig *et al*., 2024) and a block size of 500 SNPs. The resulting trees were combined with PHYLIP. This consensus topology was then used for TreeMix analyses, with 10 independent replicates run for each number of migration edges (0–10). Model fit was evaluated using the optm R package (Fitak, 2021), and based on log‐likelihood curves, the model with three migration edges was selected for downstream interpretation (Fig. S1).

LD along chromosomes was assessed as the squared Pearson correlation between pairs of loci (*r* ^2^) estimated from genotype likelihoods in 50 kbp nonoverlapping windows using the ldsep R package (Gerard, 2021). The exponential distribution of LD decay was characterized by fitting a linear regression model to the natural logarithm of *r* ^2^ values against pairwise distances up to 2500 bp apart. The slope of this regression, denoted as *λ*, thus quantifies the rate of LD decay, with a steeper slope (i.e. higher absolute value of *λ*) indicating a shorter distance to half‐decay consistent with a higher recombination rate. The overall LD within populations was estimated as the mean *r* ^2^ values after decay. For each field population, *r* ^2^ values across all genomic windows were averaged in 100 bp nonoverlapping bins to estimate the decay rate *λ* and visualize the overall LD decay by fitting a loess curve (bins of 1 and 10 bp yielded similar results; Fig. S2). LD decay rates (*λ*) within populations were compared across ploidal levels using *t*‐tests, whereas the overall LD was estimated by averaging *r* ^2^ values between 2500 and 50 000 bp and compared using Wilcoxon rank‐sum tests.

### Genome scans of adaptation to elevation in diploids and tetraploids

Patterns of differentiation and nucleotide diversity along chromosomes were used to identify loci potentially underlying adaptation by comparing low‐ and high‐elevation populations within both diploids and tetraploids, as widely applied in diploids and also proven effective in mixed‐ploid and autopolyploid systems (Yant *et al*., 2013; Arnold *et al*., 2016). To ensure robust estimates based on enough SNPs (Fig. S3), genome scans were carried out in 50‐kbp nonoverlapping windows using the ScanTools pipeline (https://github.com/pmonnahan/ScanTools). Only windows with at least 1000 sites showing < 30% missing data on a per‐population basis were considered to estimate the relative differentiation rho (Ronfort *et al*., 1998) and the absolute divergence estimator *d*_XY_ (Cruickshank & Hahn, 2014). As loci at which gene flow is reduced due to selection expectedly show both extremely high relative differentiation (rho, as described in the previous section) and absolute divergence (*d*_XY_; Cruickshank & Hahn, 2014) between populations contrasted by elevation, windows putatively under selection were identified as showing outlier values exceeding 1.5 times the interquartile range above the third quartile of the genome‐wide values. Such consistent definition of outlier windows supported comparisons between ploidal levels being independent of the total number of analyzed windows. Although rho slightly inflates intermediate values as compared to *F*_ST_, both metrics were highly correlated (Fig. S4), and rho was privileged to promote comparability of differentiation between diploids and tetraploids.

Loci potentially driven by a recent selective sweep were identified in windows showing high differentiation relative to their level of diversity. Under the expected proportionality between levels of polymorphism and divergence, a recent selective sweep produces low polymorphism while the divergence is not particularly low (i.e. excess divergence relative to diversity, compared with the genome‐wide relationship; Hahn, 2018). Following Yant *et al*. (2013), corresponding windows were identified by modeling the relationship between within‐population *π* diversity and allelic frequency differences, using major axis linear regression in the R package lmodel2. Windows with a signature of selective sweep were identified as those showing outlier residuals as estimated by the Euclidean distance to the fitted regression line that were below 1.5 times the interquartile range below the first quartile of genome‐wide values (Fig. S5). Although such a genome scan is expectedly less sensitive to the level of gene flow, it likely highlights loci being adaptively fixed in a population independently of elevation. Comparisons across ploidal levels of the total number of analyzed windows in each pair and of the number and proportion of windows identified by genome scans as putatively under selection were performed using Welch's *t*‐test and Wilcoxon rank‐sum tests.

### Candidate adaptive genes

Genes included within outlier windows for both rho and *d*_XY_, or exhibiting a signature of sweep were identified based on the annotation of the diploid *B. laevigata* genome (Beringer *et al*., 2024), using the R package plyranges (Lee *et al*., 2019) and prioritized by selecting only those genes containing at least one SNP with a rho value exceeding the corresponding rho threshold from the 50 kbp window analysis. Candidate genes were tested for enrichment of Gene Ontology (GO) biological terms using the R package topgo v.2.48.0 (Alexa *et al*., 2006) and genes from the *B. laevigata* assembly overlapping with analyzed windows as universe set. A minimum node size of 10 and the ‘weight01’ algorithm were used to favor terms being most significant, reduce the impact of nonindependent comparisons and discard any gene significantly enriched in descendent GO terms.

### Transplantation experiment across common gardens contrasted by elevation

To generate experimental families, seeds from different individuals collected in a subset of five populations (i.e. DVB, DAG, DAS, TNA, and TFU; Fig. 1) that differed in ploidal level (diploid and tetraploid) and elevation of origin (i.e. low and high elevation) in a factorial design were grown in a common garden at the University of Bern (Switzerland) for 2 yr, to standardize maternal effects, before being crossed. Within each population, reciprocal hand pollination of parental plants were performed in a pollinator‐free glasshouse and produced three to six reciprocal half‐sib seed families with at least 15 samples per crossing direction, accounting for possible maternal effects. Seeds were germinated and grown for 4 wk under standardized conditions (16 h : 8 h, 22–26°C : 16–18°C, light : dark, 65% relative air humidity, 20–40 kLux) to produce a total of 536 seedlings with four to six true leaves that were allocated and transplanted into common gardens.

Common gardens were established at 585 m asl (Ostermundigen, GPS: 46°57′59.3″N 7°29′14.3″E), 1236 m asl (Pont de Nant, GPS: 46°15′00.9″N 7°06′36.1″E) and 1967 m asl (Schynige Platte, GPS: 46°39′10.2″N 7°54′43.0″E). At each site, temperature and relative air humidity at plant height were monitored every hour throughout the experiment using four temperature loggers and two humidity loggers (DS1925L‐F5, Thermochron, and iButton DS1923‐F5, Hygrochron, respectively; Maxim Integrated Products, Wilmington, MA, USA), to assess daily, monthly and annual minimums, averages and maximums as well as growing degree days (GDD) estimated as the cumulative heat for each day above 4°C (McMaster & Wilhelm, 1997).

A total of 185, 166, and 185 seedlings evenly distributed among families were transplanted at 585, 1236, and 1967 m asl, respectively, in 1.3 l (13 × 13 × 10 cm) black plastic pots, containing standardized soil (50% field soil, 40% peat, 10% sand), and placed on top of a *c*. 3 cm sand layer covered by a black PVC foil to reduce growth of weeds. All reciprocal families with at least 10 individuals were randomly arranged at each field site and further randomized during monthly monitoring. The experiment lasted two full growing seasons (768 d).

### Phenotypic evidence of adaptation to elevation

Vegetative phenotypes were monitored monthly and included plant height (from the base of the stem to the highest point a rosette leaf reaches), rosette area (a rectangular area with sides connecting the farthest leaves), leaf number, and area of the longest leaf. At the end of the experiment, specific leaf area (SLA) was estimated by collecting two to three leaves per individual that were (1) photographed at a standardized distance to estimate their area using an imagej macro (Schneider *et al*., 2012), and (2) dried at 60°C for 48 h for weighting using an analytical balance (Denver Instrument SI‐64 Summit Series). The above ground biomass was separated from roots that were cleaned, and both were dried at 60°C for 48 h for weighting using a top‐loading precision balance (Kern PCB 6000).

Fitness‐related phenotypes were regularly assessed through the experiment. Individuals were monitored as alive or dead, enabling estimation of a survival rate per family and elevation. Fecundity was estimated by harvesting single‐seed fruits at all field sites in 2020 and 2021, after having enclosed open‐pollinated inflorescences within mesh bags allowing fruit ripening without dispersal. Fruits were oven‐dried at 60°C for 48 h and weighed to estimate the total reproductive output per plant. Ten fruits per plant and year were weighed to get the average fruit weight and estimate the number of fruits from the total reproductive output. Fitness was estimated as the family survival rate multiplied by the total number of fruits produced by this family and further normalized according to the largest fitness value.

Phenotypic differences between diploids and tetraploids from different origins were evaluated with linear mixed effect models using Satterthwaite's method in the R package ‘lmertest’ (Kuznetsova *et al*., 2017) and *post hoc* pairwise comparisons of estimated marginal means using the ‘emmeans’ package (https://cran.r‐project.org/web/packages/emmeans). The ploidy level (PL), population of origin (O), and common garden of growth at different elevation (EL), as well as their interactions, were considered as fixed effects, whereas reciprocal seed families nested within populations were used as a random effect.

## Results

### Diversity and linkage disequilibrium

The sequencing produced a median number of 48 802 302 and 104 693 657 reads per diploid and per tetraploid sample, respectively. The initial data set contained 147 020 323 genotyped sites out of which 15 463 240 (10.5%) were SNPs (Table S1). Considering only sites with a minimal depth of 4 for diploids and 8 for tetraploids, the mean individual depth ranged between 9.6 and 13.3 for diploid and 14.9 to 26.3 for tetraploid populations (Table S2).

To assess whether tetraploids of *B. laevigata* display higher genetic variation than diploids, as expected under relaxed purifying selection and higher mutational rate, the *π* nucleotide diversity and Watterson's *θ*_W_ were estimated at synonymous four‐fold (4dg) and nonsynonymous zero‐fold degenerate (0dg) sites in each field population (Table 1). Diploid and tetraploid populations showed comparable levels of genome‐wide diversity, with neither *π* (Welch's *t*‐test: *t* = −1.421, df = 9.897, *P* = 0.186), nor *θ*_W_ (Welch's *t*‐test: *t* = 1.368, df = 9.802, *P* = 0.202) significantly differing between ploidal levels at 4dg sites (Fig. 1b). Accordingly, nonsignificantly different Tajima's *D* (Wilcoxon rank‐sum test: *W* = 18, *P* = 1) confirmed comparable demographic trajectories of diploids and tetraploids of *B. laevigata*. Coherent with the accumulation of nonsynonymous variants following WGD, tetraploid populations showed significantly higher genomic variation at nonsynonymous 0dg sites, with *π*_0dg_ increased by 15.9% (Welch's *t*‐test: *t* = −2.539, df = 9.830, *P* = 0.030) and *θ*_0dg_ by 24.2% (Welch's *t*‐test: *t* = −2.622, df = 9.993, *P* = 0.0255), resulting in significantly higher ratios of *π*_0dg_/*π*_4dg_ (+4.3%, Welch's *t*‐test: *t* = −2.392, df = 9.588, *P* = 0.039) and *θ*_0dg_/*θ*_4dg_ (+10.4%, Welch's *t*‐test: *t* = −5.692, df = 9.379, *P* < 0.001) compared to diploids.

**Table 1:** Populations of *Biscutella laevigata* sampled across ploidal levels and elevation of origin, with their geographic coordinates and genetic diversity at putatively synonymous and nonsynonymous sites.

Pop	PL	O	Lon.	Lat.	*π*_4dg_	*π*_0dg_	*π*_0dg_/*π*_4dg_	*θ*_W 4dg_	*θ*_W 0dg_	*θ*_W 0dg_/*θ*_W 4dg_	TajD_4dg
DVB	2*x*	720	8.9833	48.0513	0.0096	0.0028	0.2947	0.0091	0.0027	0.3002	0.3007
DKS	2*x*	380	15.4472	48.293	0.0081	0.0024	0.2988	0.0078	0.0024	0.3048	0.2773
DAG	2*x*	400	16.1775	47.9717	0.0112	0.0033	0.2933	0.0116	0.0036	0.3094	−0.1967
DBM	2*x*	1880	5.2786	44.1741	0.0119	0.0033	0.2804	0.0125	0.0037	0.2937	−0.2836
DPR	2*x*	1800	11.1475	45.6965	0.0093	0.0029	0.3114	0.0095	0.0031	0.3258	−0.1171
DAS	2*x*	1760	15.6092	47.6968	0.0100	0.0029	0.2921	0.0103	0.0031	0.3053	−0.1377
TFU	4*x*	460	7.0704	46.1208	0.0091	0.0029	0.3145	0.0089	0.0030	0.3404	0.1175
TBU	4*x*	1200	11.1165	47.3394	0.0107	0.0033	0.3103	0.0109	0.0037	0.3389	−0.0755
TPR	4*x*	880	13.5723	46.4655	0.0112	0.0035	0.3141	0.0119	0.0042	0.3492	−0.2772
TNA	4*x*	2000	6.9795	46.4342	0.0118	0.0035	0.2996	0.0122	0.0040	0.3285	−0.1182
TDS	4*x*	2360	9.824	46.8339	0.0109	0.0034	0.3127	0.0112	0.0038	0.3443	−0.1030
TRA	4*x*	1700	13.6171	47.4487	0.0128	0.0038	0.2955	0.0133	0.0044	0.3287	−0.1573

The subset of populations (Pop) used for transplantation across elevation are underlined. Ploidal levels (PL) are abbreviated as 2*x* for diploids and 4*x* for tetraploids. The elevation of origin (O) is reported in m asl, Geographic coordinates are reported as longitude (Lon.) and latitude (Lat.). Genetic diversity was characterized using *π* and theta Watterson's (*θ*_W_) at putatively synonymous (4dg) and nonsynonymous (0dg) sites.

Consistent with previous studies in *B. laevigata*, population tree and migration events inferred using TreeMix showed interploidal admixture (Fig. 1a) and supported pairs of populations contrasted by elevation in both diploids (DAG‐DAS, DKS‐DPR, DVB‐DBM) and tetraploids (TFU‐TNA, TBU‐TDS, and TPR‐TRA). As expected from the recent origin of tetraploids, pairwise genetic differentiation rho, which accounts for the expectedly slower neutral divergence of tetraploids and thus allows comparisons across ploidal levels, was significantly higher among diploid than among tetraploid populations when considering all pairwise comparisons within each ploidal level (Welch's *t*‐test: *t* = 2.591, df = 10, *P* = 0.027; Table S3) despite a large overlap in rho values between diploids and tetraploid (0.113 to 0.373 in diploids; 0.180–0. 309 in tetraploids). However, among the three low‐ vs high‐elevation pairs used in genome scans the genetic differentiation between ploidal levels did not differ significantly (Welch's *t*‐test: *t* = 0.703, df = 2.317, *P* = 0.546; Fig. 1b).

The rate of LD decay in diploids vs tetraploids highlighted no significant difference (Welch's *t*‐test: *t* = −1.248, df = 9.466, *P* = 0.242), indicating similar distances to half‐decay and hence recombination rates in populations of both ploidal levels. By contrast, mean *r* ^2^ after the decay revealed higher overall LD in diploids than in tetraploids (Wilcoxon rank‐sum test: *W* = 36, *P* = 0.002; Fig. 1c), matching the higher genetic differentiation and isolation of diploid populations at the periphery of the Alps at a biogeographic scale.

### Genomic signatures of adaptation to elevation in diploids and tetraploids

Genome scans of low‐ vs high‐elevation population pairs within each ploidal level included between 11 591 and 13 412 genomic windows of 50 kbp per population pair, encompassing 91% to 96% of annotated gene models (Table S4). The number of windows analyzed did not significantly differ between diploids and tetraploids (Welch's *t*‐test: *t* = 0.270, df = 3.280, *P* = 0.803). Loci at which gene flow is reduced due to selection were identified as expectedly showing both high differentiation (rho) and absolute divergence (*d*_XY_). In diploids, only the geographically close pair of populations with the lowest genome‐wide differentiation (DAG‐DAS) showed outlier rho and *d*_XY_ values, detected in 0.44% of the analyzed windows, mainly in central segments of chromosomes that also show high repeat content and gentle LD decay (i.e. *λ* close to 0; Fig. 2a). By contrast, the two other pairs of diploid populations showed no rho outlier window and, consequently, no outlier window for both rho and *d*_XY_ (Fig. S6). The three pairs of tetraploid populations contrasted by elevation showed 0.04% to 0.10% of outlier windows indicative of multiple candidate adaptive loci, mainly across distal chromosome segments showing high gene density and steep LD decay (i.e. higher absolute value of *λ*; Fig. S6). Overall, proportions of such outlier windows did not significantly differ between ploidal levels (Wilcoxon rank‐sum test: *W* = 3, *P* = 0.658; Fig. 2b).

**Fig. 2:**
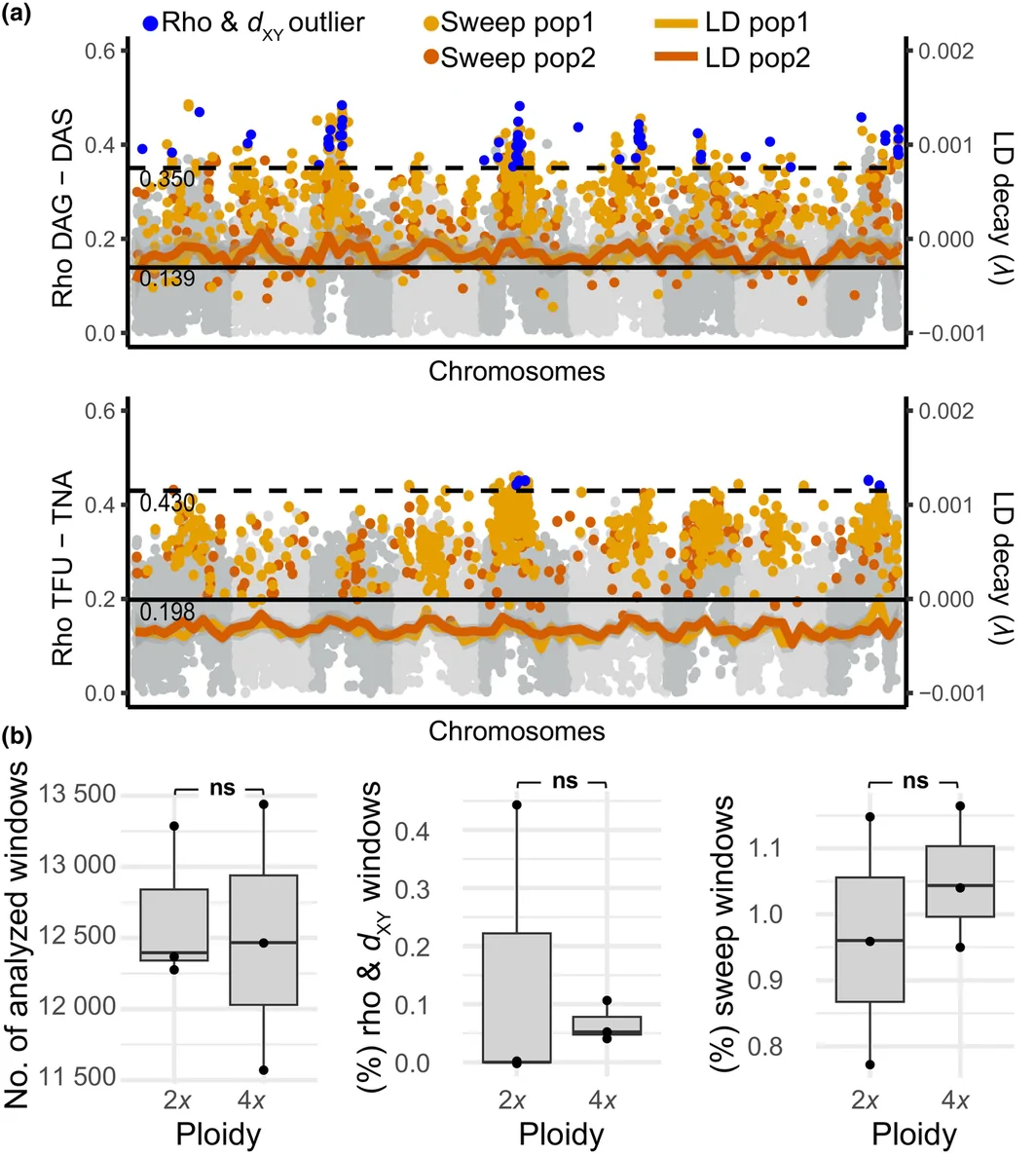
Genome scans identifying signature of selection between pairs of diploid (2*x*) and tetraploid (4*x*) populations of *Biscutella laevigata* contrasted by elevation. (a) Differentiation (rho) along chromosomes of 2*x* (top; DAG vs DAS) and 4*x* (below; TFU vs TNA) populations, with outlier windows for both relative differentiation (rho) and absolute divergence (*d*_XY_) highlighted in blue. Loci putatively driven by a selective sweep are shown as windows showing an outlier differentiation relative to their level of diversity in the population at low (yellow) or high (orange) elevation. Lines show the linkage disequilibrium decay (*λ*) in corresponding populations. All pairs of 2*x* and 4*x* populations are shown in Supporting Information Fig. S2. (b) Total number of scanned windows analyzed among pairs of populations (left), and their proportions being either outlier for both rho and *d*_XY_ (centre), or showing a signature of sweep (right). Significance assessed using Welch's *t*‐tests (number of analyzed windows and proportion of sweep windows) and Wilcoxon rank‐sum test (proportion of rho and *d*_XY_ windows), with *P*‐values considered as nonsignificant (ns) when > 0.05.

The proportion of windows indicative of recent selective sweep did not significantly differ between ploidal levels (*t* = −0.716, df = 3.171, *P* = 0.523; Fig. 2b) and differences in the proportions of windows showing a signature of sweep at low vs high elevation were significant in neither diploid (Welch's *t*‐test: *t* = 0.214, df = 2.784, *P* = 0.846) nor tetraploid populations (Welch's *t*‐test: *t* = −0.505, df = 2.821, *P* = 0.650), supporting similarly selective environments that have shaped genetic differentiation in both ploidal levels.

In diploids, the 13 candidate adaptive genes identified as rho and *d*_XY_ outliers (i.e. 13 in DAG‐DAS, 0 in DVB‐DBM, and 0 in DKS‐DPR; Table S5) were enriched for processes related to primary metabolism (lysine biosynthesis), protein trafficking, and stress responses (including regulation of salicylic acid‐mediated signaling, anthocyanin biosynthesis, and response to toxic compounds; Table S6), consistent with divergence along elevational gradients. In tetraploids, the two enriched processes involving the higher number of genes among the 36 rho and *d*_XY_ outliers candidate (i.e. 3 in TFU‐TNA, 28 in TBU‐TDS, and 5 in TPR‐TRA; Table S7) were regulation of flower development and heterochromatin formation, with further enrichments in developmental transitions and reproduction (vegetative‐to‐reproductive phase change, pollen maturation), gene regulation (DNA methylation–dependent heterochromatin formation, mRNA stabilization), metabolic functions (thalianol and flavonoid metabolism, acetyl‐CoA biosynthesis, and glycolysis), and stress responses (nitrate and sodium signaling, desiccation tolerance, and stomatal regulation) again pointing to pathways potentially underlying adaptation to elevation (Table S8).

By contrast, candidate genes detected under sweep signatures may reflect both adaptation to elevation and local adaptation unrelated to elevational divergence. In diploids, the 85 sweep candidates (Table S5) were enriched in diverse responses to environmental factors (Table S6). Beyond responses to toxic substances, anthocyanin biosynthesis, and systemic resistance, several loci were linked to temperature stress (e.g. heat stress transcription factors) and flowering time (e.g. Frigida). In tetraploids, the 236 sweep candidate genes (Table S7) were enriched in signal transduction, defense response (salicylic acid‐mediated signaling), reproduction and floral development (floral organ formation, asymmetric cell division), and gene expression regulation (cytoplasmic translation, transcription and translation initiation, RNA processing), pointing to a broader spectrum of local adaptive processes (Table S8).

Genome scans comparing all diploids to all tetraploids similarly identified candidate genes underlying adaptation to WGD, highlighting several cellular processes but no gene related to meiosis as detailed in Notes S1.

### Phenotypic evidence of adaptation to elevation in diploids vs tetraploids

Seeds produced from the five populations were transplanted in three common gardens across elevation (i.e. at 585, 1236 and 1967 m asl). As expected, annual mean temperatures measured at lower elevation were higher than at other sites (Wilcoxon rank sum test *P*‐value < 0.001; Fig. S7), with the GDD showing much higher cumulative heat for days above 4°C and confirming almost continuous growth at low elevation (summed up annual GDD = 70 627) as compared to the medium and high elevation sites showing lower GDD (38 175 and 38 523, respectively). Consistent with a WGD‐induced gigas effect, phenotypes of transplanted plants monitored over two growing seasons indicated larger leaf and rosette area in tetraploids, independently of the environment. In addition, all traits except the root/shoot biomass ratio, which was strongly affected by the elevation of growth, showed significant interactions between elevations of origin and of growth in linear mixed effect models, and hence pointed to significant phenotypic differentiation across elevation in both ploidal levels (Table 2). However, as expected with phenotypic plasticity largely unaltered following WGD, most phenotypes (i.e. all except number of leaves and leaf area) showed nonsignificant interactions between the ploidal level and the elevation of growth, supporting similar reaction norms and phenotypic plasticity in response to the environment between diploids and tetraploids (Fig. S8). Some phenotypes showed significant differences in specific environments only (i.e. larger root/shoot biomass ratio at high elevation) or no differences between ploidal levels (i.e. number of leaves and SLA; Fig. 3a), indicating that neither growth rate nor resource allocation has been dramatically altered following WGD.

**Table 2:** Linear mixed‐effects models of phenotypes among individuals of *Biscutella laevigata* transplanted across elevation, testing the effect of ploidy level (PL: 2*x* vs 4*x*), population origin (O: lowland vs alpine), elevation of growth in common gardens (EL: 585, 1236, and 1967 m), and their interactions, with *F*‐statistics, their significance (***, *P* < 0.001; **, *P* < 0.01; *, *P* < 0.05; ns, *P* > 0.05), and the denominator degrees of freedom (df) reported for each term.

Phenotypic trait		Population origin (O)	Elevation level (EL)	Ploidy level (PL)	O × EL	O × PL	EL × PL	O × EL × PL	*R* ^2^_m_	*R* ^2^_c_
Number of leaves		28.2*** (df = 15.9)	43.9*** (df = 451.4)	0.3 (df = 15.9)	15.7*** (df = 451.4)	0.4 (df = 15.9)	5.3** (df = 451.4)	3.7* (df = 451.4)	0.40	0.54
Leaf area (mm^2^)		6.83* (df = 19.02)	17.40*** (df = 457.36)	103.07*** (df = 19.02)	7.71*** (df = 457.36)	30.15*** (df = 19.02)	3.56* (df = 457.36)	1.38 (df = 457.36)	0.45	0.49
Rosette area (cm^2^)		5.0* (df = 18.2)	2.1 (df = 454.4)	50.5*** (df = 18.2)	12.1*** (df = 454.4)	7.0* (df = 18.2)	1.3 (df = 454.4)	5.7** (df = 454.4)	0.36	0.44
Plant height (mm)		11.6** (df = 18.5)	36.1*** (df = 445.5)	12.7** (df = 18.5)	12.7*** (df = 445.5)	10.7** (df = 18.5)	1.0 (df = 445.5)	3.2* (df = 445.5)	0.36	0.44
Specific leaf area (mm^2^ mg^−1^)		5.4* (df = 20.2)	150.1*** (df = 261.8)	0.1 (df = 20.2)	6.1** (df = 261.8)	0.1 (df = 20.2)	1.2 (df = 261.8)	0.2 (df = 261.8)	0.59	0.63
Root : shoot biomass ratio (Box‐Cox transformed)		4.7* (df = 19.0)	30.2*** (df = 282.4)	3.0 (df = 19.0)	0.7 (df = 282.4)	0.6 (df = 19.0)	0.3 (df = 282.4)	2.0 (df = 282.4)	0.21	0.22
Fitness index		27.1*** (df = 17.3)	70.7*** (df = 84.2)	6.0* (df = 17.3)	9.3*** (df = 84.2)	3.7 (df = 17.3)	18.1*** (df = 84.2)	0.7 (df = 84.2)	0.73	0.75
∆Fitness		9.1** (df = 16.7)	–	13.5** (df = 16.7)	–	0.2 (df = 16.7)	–	–	0.53	0.83
Fecundity	2020	45.0*** (df = 18.7)	98.0*** (df = 456.6)	14.0** (df = 18.7)	5.4** (df = 456.6)	0.0 (df = 18.7)	24.6*** (df = 456.6)	1.5 (df = 456.6)	0.52	0.54
	2021	16.2*** (df = 20.6)	23.2*** (df = 432.7)	0.2 (df = 20.6)	13.6*** (df = 432.7)	0.7 (df = 20.6)	0.6 (df = 432.7)	0.8 (df = 432.7)	0.26	0.29
Survival rate	2020	0.0 (df = 16.1)	8.7*** (df = 82.3)	0.5 (df = 16.1)	11.8*** (df = 82.3)	17.2** (df = 16.1)	8.7*** (df = 82.3)	5.5** (df = 82.3)	0.47	0.59
	2021	1.1 (df = 14.7)	5.6*** (df = 81.4)	0.3 (df = 14.7)	5.6** (df = 81.4)	9.9** (df = 14.7)	4.2* (df = 81.4)	5.1** (df = 81.4)	0.32	0.43

*R* ^2^_m_, marginal pseudo‐*R* ^2^ coefficients for fixed effects, *R* ^2^_c_, conditional *R* ^2^ for both random and fixed effects. Significant terms are shown in bold.

**Fig. 3:**
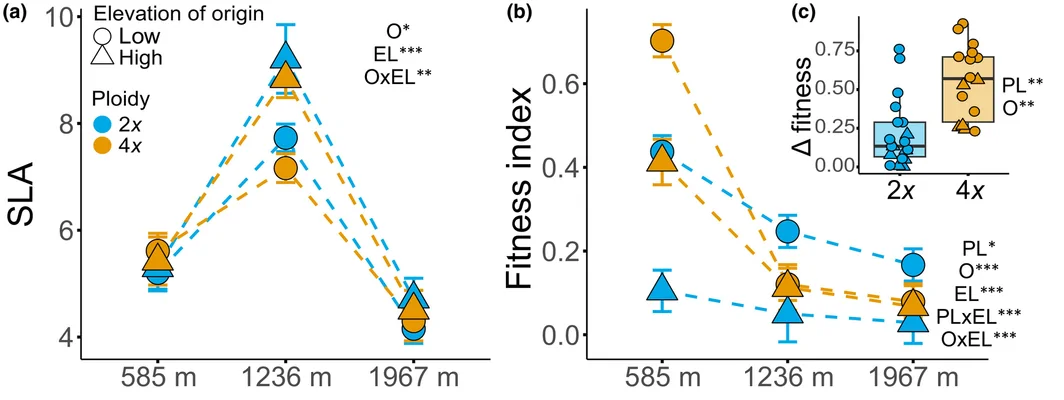
Phenotypes of diploids (2*x*) and tetraploids (4*x*) of *Biscutella laevigata* from low and high elevation transplanted across elevation in gardens at 585, 1236 and 1967 m above sea level (asl). (a) Specific leaf area (SLA) and (b) fitness index using linear mixed models plotted with estimated marginal means ±SE plotted across elevation and significant fixed effects shown as ‘Population origin’ (O), ‘Elevation level’ (EL), ‘Ploidy level’ (PL) and their interactions. SLA is mostly controlled by the environment, whereas the significant OxEL indicates that the population origin affects the reaction norm, in contrast to ploidal levels that show no differences in response to environment. The full set of traits analyzed are presented in Table 2 and shown in Supporting Information Fig. S4. The fitness index (survival multiplied by reproductive output) highlighted adaptation to elevation in tetraploids, that outperformed all populations at the low‐elevation site (585 m), whereas fitness differences between ploidal levels became nonsignificant at high elevation. The significant PLxEL interaction indicates that ploidy levels differ in their phenotypic plasticity. This is further highlighted by (c) showing ∆ fitness (the difference between minimum and maximum fitness within each family), which was significantly higher in tetraploids than in diploids. In both ploidal levels, populations originating from high elevation further showed greater fitness homeostasis. Significance levels: ***, *P* < 0.001; **, *P* < 0.01; *, *P* < 0.05.

The fitness index integrating survival and reproductive output highlighted different adaptive responses to elevation in diploids and tetraploids as indicated by the significant interaction between ploidal level and elevation of growth (Table 2). Tetraploids significantly outperformed diploids at low elevation, as likely driven by a strong advantage of lowland tetraploids. However, fitness differences between ploidal levels and elevation of origin were no longer significant when plants were grown at high elevation (Fig. 3b). Consequently, diploids showed greater fitness homeostasis across elevations, whereas tetraploids showed considerable fitness variation across elevation, supporting genetically based adaptation in response to the environment. The elevation of origin also significantly affected fitness homeostasis, with alpine populations displaying greater fitness homeostasis across both ploidal levels (Table 2; Fig. 3c). Such variation in fitness depending on the environment of growth was largely inherent to the reduced survival of tetraploids from low elevation when grown at high elevation (Fig. S9). As this further emphasizes that these tetraploids were maladapted to high elevation and strongly responded to selection in the course of the experiment, the fecundity of the 461 plants having survived to reproduction (out of the 536 initially transplanted) was significantly higher in tetraploids than in diploids in the first year of monitoring (Table 2; Fig. S10).

## Discussion

As expected from the evolutionary history of the strictly outcrossing *B. laevigata*, diploid populations, which began diverging in spatial isolation since the onset of the last ice age (> 50 000 generations ago), generally show higher genetic differentiation and overall LD than tetraploids that originated from a WGD event *c*. 24 000 to 7000 generations ago and subsequently expanded postglacially across the Alps with ongoing gene flow among populations (Parisod, 2022; Grünig *et al*., 2024). Despite their contrasted histories, diploids and tetraploids did not differ in genetic differentiation among our focal low‐ vs high‐elevation population pairs. Likewise, Tajima's D supported similar demographic footprints for both ploidal levels, which also showed no difference in genome‐wide diversity at synonymous sites (*π*_4dg_ and *θ*_4dg_). Tetraploids have hence accumulated considerable putatively neutral genetic variation since their origin, although below the expected effect of a doubled effective population size following WGD that has been predicted by simulations to be reached only after half a million of generations (Vlček *et al*., 2025). By contrast, both metrics of diversity at nonsynonymous sites (i.e. *π*_0dg_ and *θ*_0dg_) were significantly higher in tetraploids than in diploids, as expected with an increased masking of (partially recessive) deleterious alleles under tetrasomic inheritance following WGD. This aligns well with the accumulation of nonsynonymous variants reported in *Arabidopsis arenosa* following WGD (Monnahan *et al*., 2019). In contrast to *A. arenosa* (Arnold *et al*., 2016), interspecific gene flow can be ruled out in *B. laevigata*, although the role of adaptive inter‐ploidy gene flow and increased mutational input in tetraploids remain satisfactorily delineated in neither case.

How ploidal levels of *B. laevigata* responded to selection was examined through both indirect genome scans and experimental transplantation of pairs of diploid and tetraploid populations across contrasted elevations. Using conservative reverse genetics of adaptation approaches (Bomblies & Peichel, 2022), we identified multiple loci putatively under selection in each ploidal level, independently of the proportion of the genome being scanned. Furthermore, as LD decay was similar between ploidal levels, these footprints of selection by elevation appear independent of linked selection. Overall, the proportion of candidate outliers for either extreme differentiation of divergent alleles in the face of gene flow (i.e. outliers for both rho and *d*_XY_, Cruickshank & Hahn, 2014) did not differ between diploids and tetraploids. However, in diploids such clear signatures of adaptation to elevation assuming equilibrium between gene flow and selection were detected only between nearby DAG‐DAS populations, whereas in tetraploids all three population pairs showed candidate adaptive loci with credible functional annotations related to elevation. This pattern is consistent with higher levels of gene flow in tetraploids, which can enhance the detectability of multilocus adaptation in genome scans, and indicates that selection is an effective driver of variation in response to environmental changes following WGD. Finally, diploids and tetraploids showed similar proportions of loci showing evidence of selective sweep, which may be consistent with quicker sorting of variation in tetraploids following the WGD‐induced increased effective population size (Otto & Whitton, 2000).

Provided the limited phenotypic impact of WGD *per se* (Grünig *et al*., 2026), direct estimates from fitness‐related phenotypes fully support that tetraploids of *B. laevigata* have effectively responded to selection, refuting long‐held predictions that WGD is an adaptive dead‐end. As expected from classical experiments (Petit *et al*., 1999; Bretagnolle & Thompson, 2001), most phenotypes showed high plasticity and similar reaction norms in both ploidal levels of *B. laevigata*, which is further consistent with a negligible impact of WGD on phenotypic plasticity (Grünig *et al*., 2026). However, diploids and tetraploids differed regarding the fitness impact of such plastic phenotypes. Beyond genetically based phenotypic differentiation shown by both ploidal levels, phenotypic plasticity among diploids from low vs high elevation promoted a relatively constant fitness (i.e. homeostasis) across elevation, indicating general‐purpose genotypes possibly suffering from higher inbreeding depression than tetraploids (Clo & Kolář, 2022) and relying on adaptive phenotypic plasticity to thrive in heterogeneous environments (Grünig *et al*., 2024), as commonly reported in plants (Palacio‐López *et al*., 2015). By contrast, tetraploids of *B. laevigata* showed higher fitness variation across environments that supports genetically based adaptation to elevation following WGD. Accordingly, low‐elevation tetraploids were shown to thrive in their habitat but hardly survive at high elevation, suggesting limited adaptive phenotypic plasticity as compared to diploids that showed far less extreme fitness consequences of environmental changes. Both indirect and direct evidence of adaptation across elevation in *B. laevigata* hence markedly contrasted with predictions that WGD hinders genetic and phenotypic adaptation and rather point to strong and rapid post‐WGD responses to selection in tetraploids.

Having postglacially tracked their ecological niche under pervasive purifying selection in the face of local extinction and ongoing fragmentation of their distribution range (Rosche *et al*., 2022), diploid populations were here shown to exhibit limited adaptation to elevation, but seemingly optimized their phenotypic reaction norms to maintain fitness across a large range of the growing environments. Contrastingly, tetraploid populations adapted genetically across heterogenous environments. Such adaptive potential may reflect the accumulation of variation under relaxed purifying selection in tetraploids, suggesting that WGD has promoted the adaptive sorting of genetic variants, matching evidence from experimental evolution of polyploid yeasts supporting rapid adaptation to new habitats (Selmecki *et al*., 2015). Both the origin and the fitness‐effect of identified adaptive variants will have to be assessed in future studies to understand the genetic architecture of adapted traits along ecological gradients confounded with elevation (e.g. temperature and herbivory) and to decipher how tetraploids of *B. laevigata* favored polygenic adaptation to a larger extent than spatially isolated diploids. More generally, most plants having undergone multiple WGD‐dysploidy cycles (Huang *et al*., 2026), it looks necessary to further assess the balance between costs and benefits from natural selection in response to endogenous challenges (i.e. adaptation to WGD) and external opportunities (i.e. adaptation through WGD) to better appreciate the adaptive consequences of recurrently duplicated genes (Van de Peer *et al*., 2017).

## Competing interests

None declared.

## Author contributions

SG, MB and CP designed the research and interpreted and wrote the manuscript. SG collected and analyzed the genomic data. MB collected and analyzed the phenotypic data.

## Disclaimer

The New Phytologist Foundation remains neutral with regard to jurisdictional claims in maps and in any institutional affiliations.

## Supporting information


**Fig. S1** TreeMix model likelihood.
**Fig. S2** Decay of linkage disequilibrium.
**Fig. S3** Number of SNPs per genomic window.
**Fig. S4** Spearman correlations between rho and FST metrics.
**Fig. S5** Distribution of differentiation and diversity proxies.
**Fig. S6** Genome scans in population pairs from low vs high elevation in *Biscutella laevigata*.
**Fig. S7** Design of the transplant experiment in *Biscutella laevigata*.
**Fig. S8** Phenotypes analysis in the transplant experiment.
**Fig. S9** Survival rate in the transplant experiment.
**Fig. S10** Fecundity in the transplant experiment.
**Notes S1** Adaptation to whole‐genome duplication (WGD) in *Biscutella laevigata*.
**Table S1** Genotyping of the 96 samples of *Biscutella laevigata*.
**Table S2** Genotyping in the 12 populations of *Biscutella laevigata*.
**Table S3** Differentiation at synonymous sites between pairs of populations.
**Table S4** Summary of genome scans between pairs of populations.
**Table S5** Candidate genes underlying adaptation to elevation in diploids.
**Table S6** Gene Ontology terms significantly enriched in diploids.
**Table S7** Candidate genes underlying adaptation to elevation in tetraploids.
**Table S8** Gene Ontology terms significantly enriched in tetraploids.
Please note: Wiley is not responsible for the content or functionality of any Supporting Information supplied by the authors. Any queries (other than missing material) should be directed to the *New Phytologist* Central Office.

## Data Availability

Sequencing data are available at: https://www.ebi.ac.uk/ena/browser/view/PRJEB48987.
