# Supplementary material for: Whole‐genome duplication promotes phenotypic and genetic adaptation to elevation
Source: New Phytol. 2026 Sep 21;252(4):1620–31. doi: 10.1111/nph.71572 (PMC13669793; doi:10.1111/nph.71572)
Supplement: Supplementary file 1 [file NPH-252-1620-s001.pdf]

New Phytologist

***New Phytologist* Supporting Information**

Article title: Whole-genome duplication promotes phenotypic and genetic adaptation to elevation
Authors: Sandra Grünig, Marc Beringer & Christian Parisod
Article acceptance date: 20 August 2026

The following Supporting Information is available for this article:

Fig. S1 – TreeMix model likelihood 2
Fig. S2 – Decay of linkage disequilibrium 3
Fig. S3 – Number of SNPs per genomic window 4
Fig. S4 – Spearman correlations of rho and F_{ST} metrics 5
Fig. S5 – Distribution of differentiation and diversity proxies 6
Fig. S6 – Genome scans in population pairs from low vs. high elevation 7
Fig. S7 – Design of the transplant experiment 8
Fig. S8 – Phenotypes analysis in the transplant experiment 9
Fig. S9 – Survival rate in the transplant experiment 10
Fig. S10 – Fecundity in the transplant experiment 11
Table S1 – Genotyping of the 96 samples of Biscutella laevigata 12
Table S2 – Genotyping in the twelve populations of Biscutella laevigata 15
Table S3 – Differentiation at synonymous sites between pairs of populations 16
Table S4 – Summary of genome scans between pairs of populations 17
Table S5 – Candidate genes underlying adaptation to elevation in diploids 18
Table S6 – Gene ontology terms significantly enriched in diploids 22
Table S7 – Candidate genes underlying adaptation to elevation in tetraploids 23
Table S8 - Gene ontology terms significantly enriched in tetraploids 52
Notes S1: Adaptation to whole-genome duplication (WGD) 54

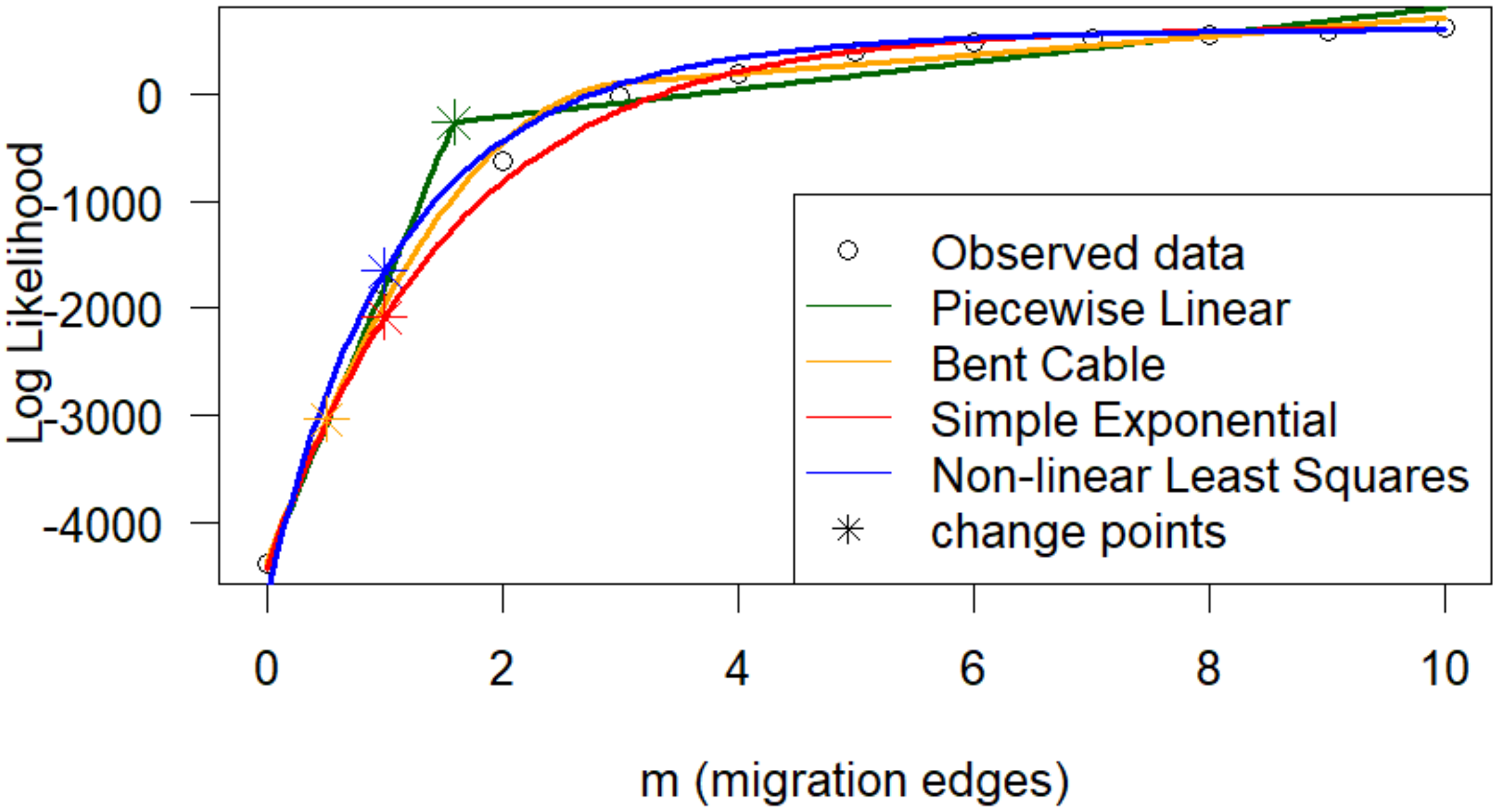

Fig. S1 – TreeMix model likelihood

TreeMix model likelihood across models with 0–10 migration edges. The model with three migration edges was selected for downstream analyses.

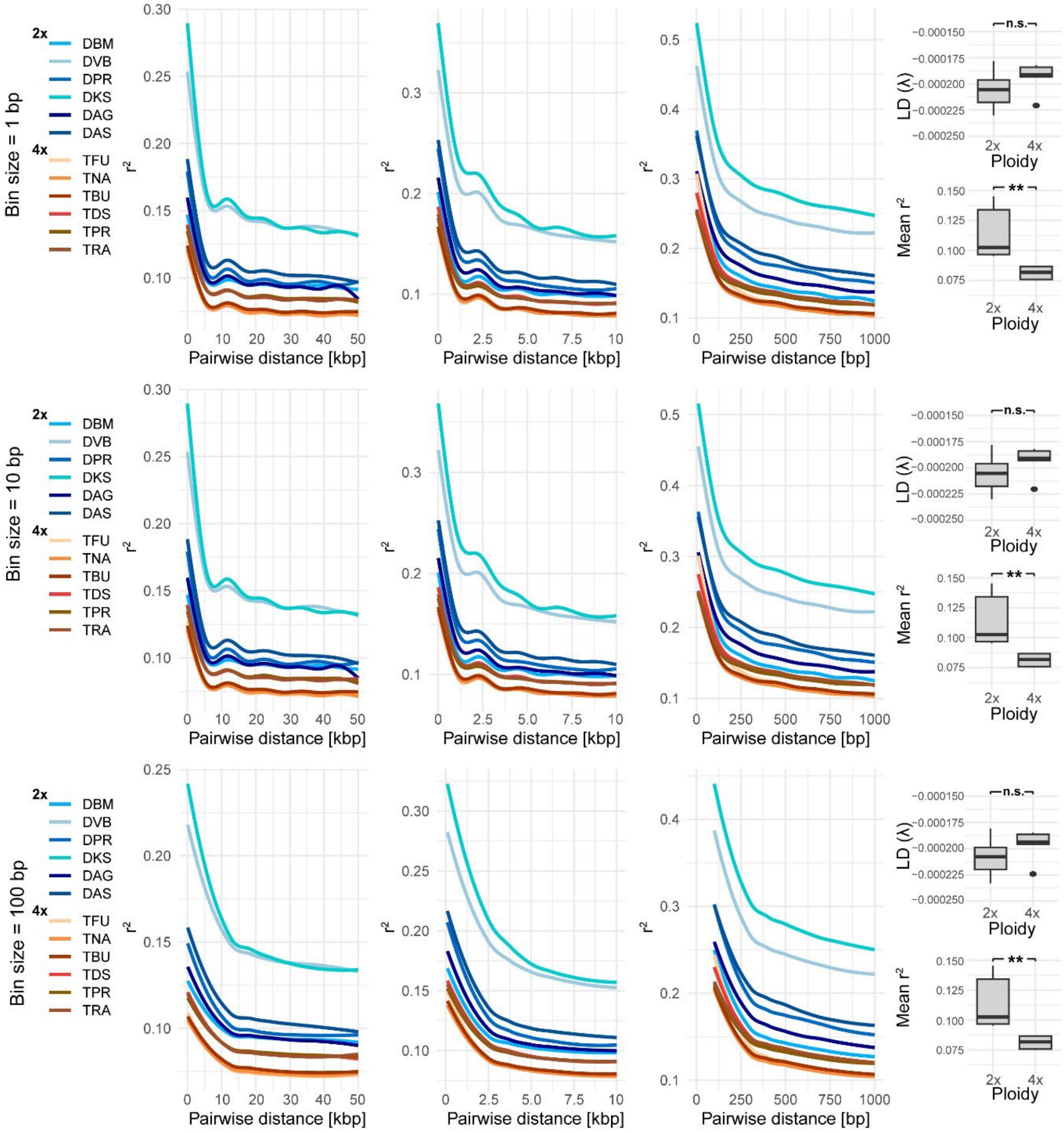

Fig. S2 – Decay of linkage disequilibrium

Decay of linkage disequilibrium in *Biscutella laevigata* approximated as Pearson genotypic correlations (r^2) as a function of distance between sites within each population. r^2 values were averaged in non-overlapping bins of 1 bp (top), 10 bp (middle), or 100 bp (bottom) to estimate an overall decay rate λ per population. LD decay rates λ were compared across ploidal levels using t-test, whereas mean r^2 values after decay (i.e. between 2,500 and 50,000 bp) were compared using Wilcoxon rank-sum tests. Significance levels: **$p < 0.01$, n.s. $p > 0.05$

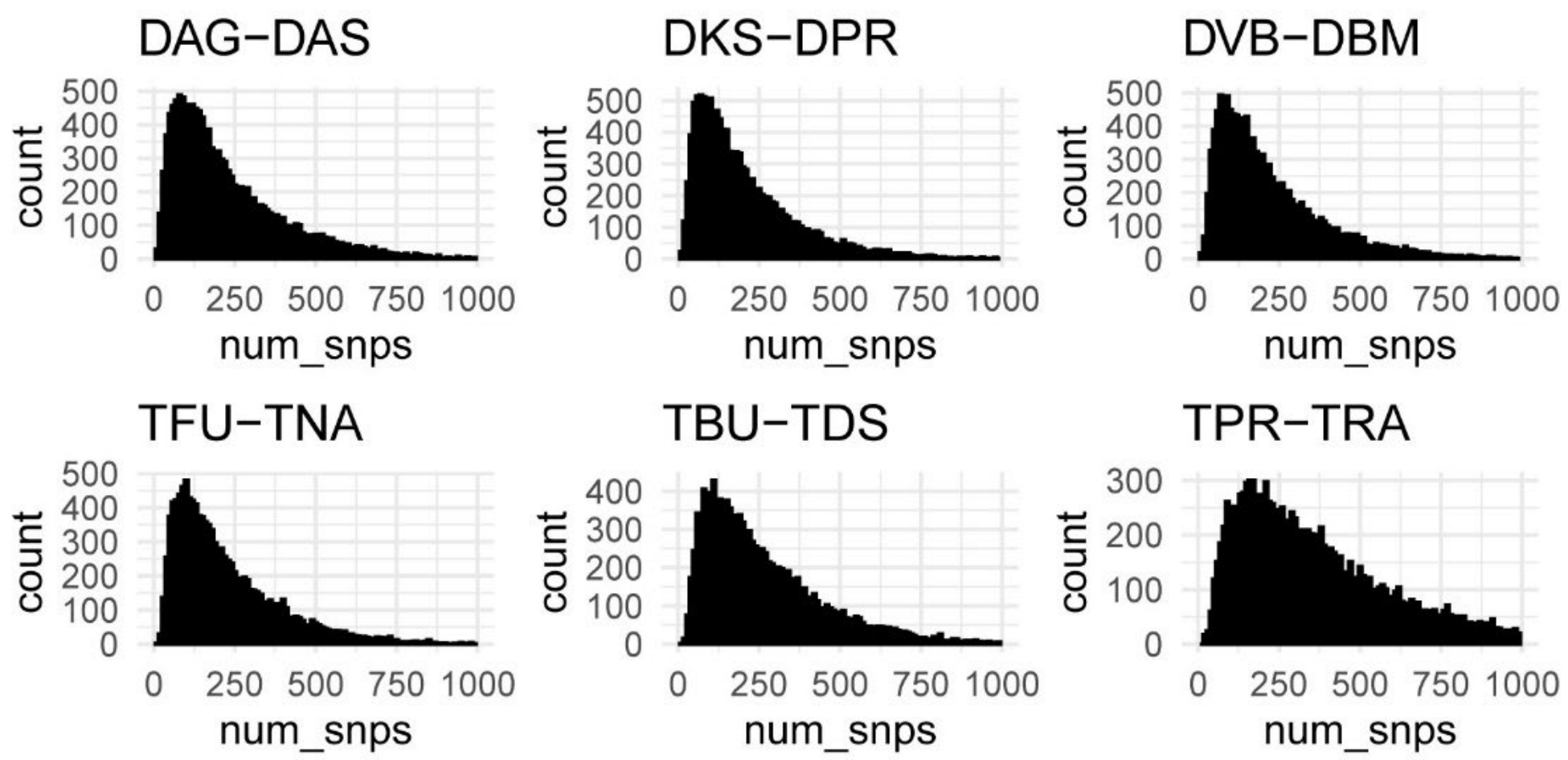

Fig. S3 – Number of SNPs per genomic window

Histograms of the number of SNPs (num_snps) per 50 kbp genomic window in pairs of low vs high elevation populations within each ploidal level of *Biscutella laevigata*. Populations are coded as in Table 1.

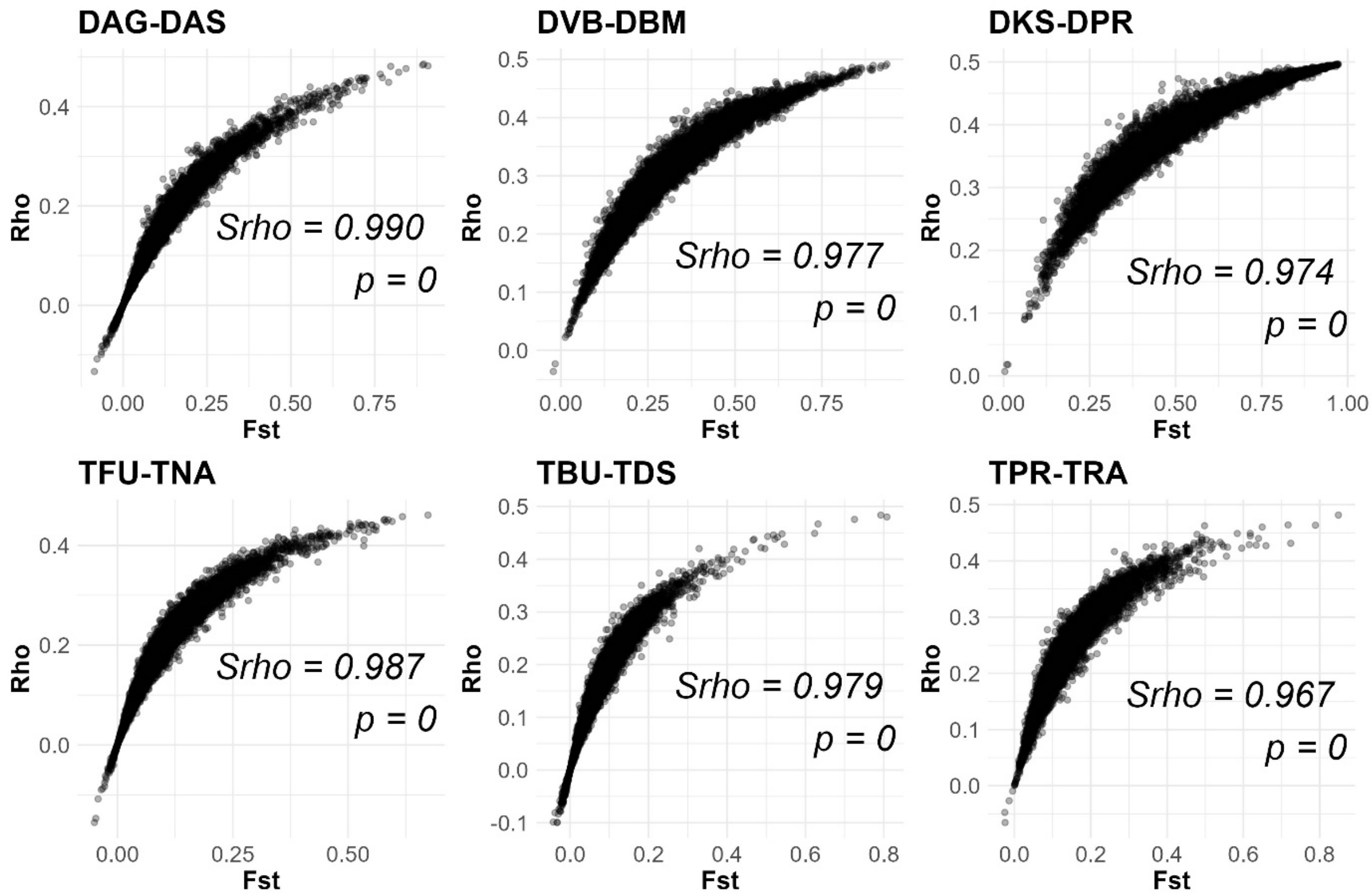

Fig. S4 – Spearman correlations of rho and F_{ST} metrics

Spearman correlations between genetic differentiation metrics F_{ST} and rho across genomic windows of paired low- vs. high-elevation populations of diploid and tetraploid of *Biscutella laevigata*. For each pair, Spearman correlation coefficients (reported as Srho to avoid confusion with the rho differentiation metric) and associated significance values are shown; p = 0 indicates p-values < 10^{-16}.

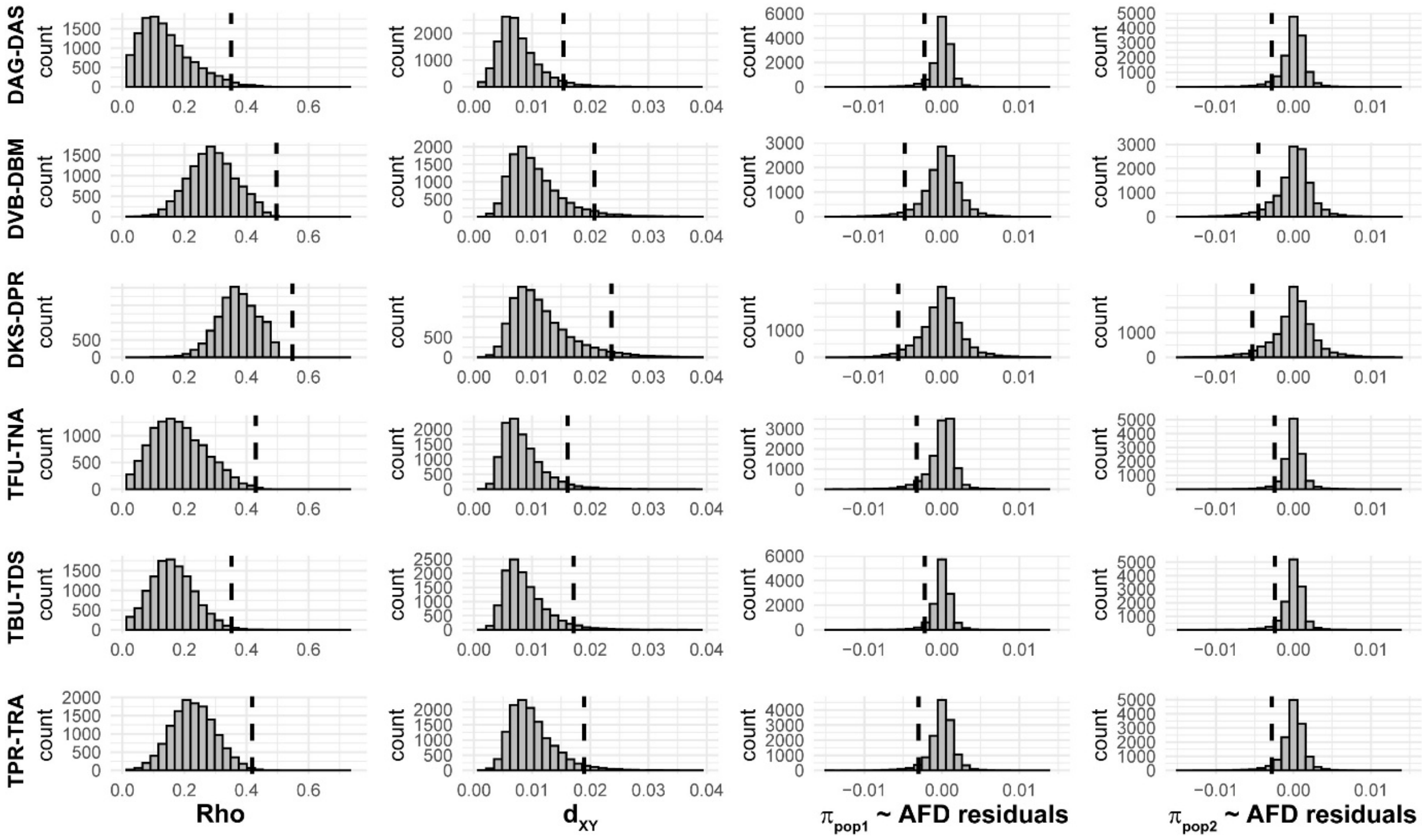

Fig. S5 – Distribution of differentiation and diversity proxies

For each pair of low vs high elevation populations within ploidal level of *Biscutella laevigata*, histograms of rho, d_{XY}, residuals of the π_{pop1}~AFD, and residuals of the π_{pop2}~AFD. AFD is the allelic frequency difference and dashed line show thresholds defining outliers. Populations are coded as in Table 1.

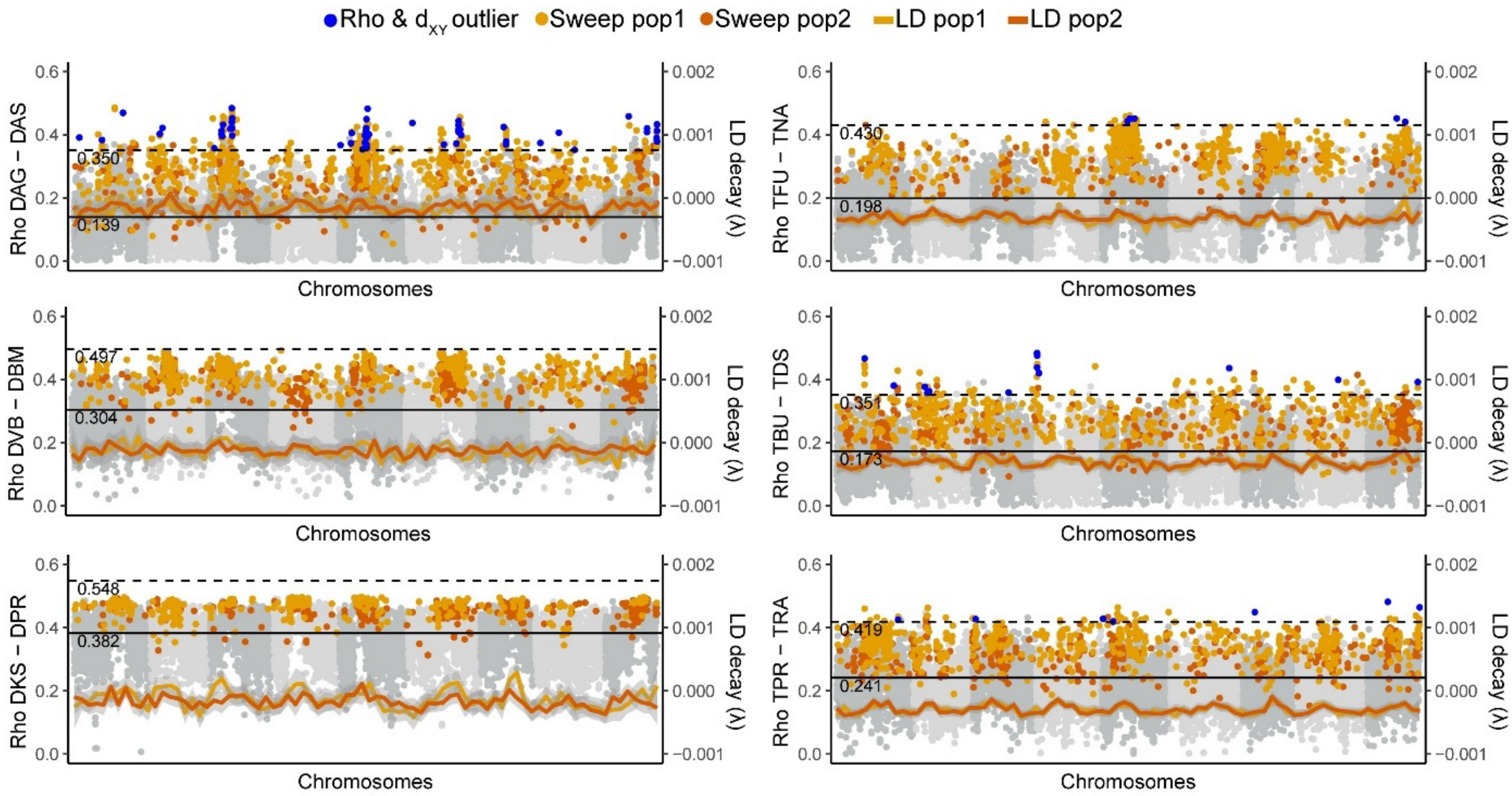

Fig. S6 – Genome scans in population pairs from low vs. high elevation in *Biscutella laevigata*

Genome scans of diploid (left) and tetraploid (right) population pairs from low vs. high elevation. The solid black line shows the genome-wide rho value and the dashed line shows the rho threshold used to define outliers (i.e. 1.5 times the interquartile range above the third quartile of the genome-wide values). Candidate windows are highlighted according to the top panel. Yellow and orange lines show the linkage disequilibrium (LD) decay (λ).

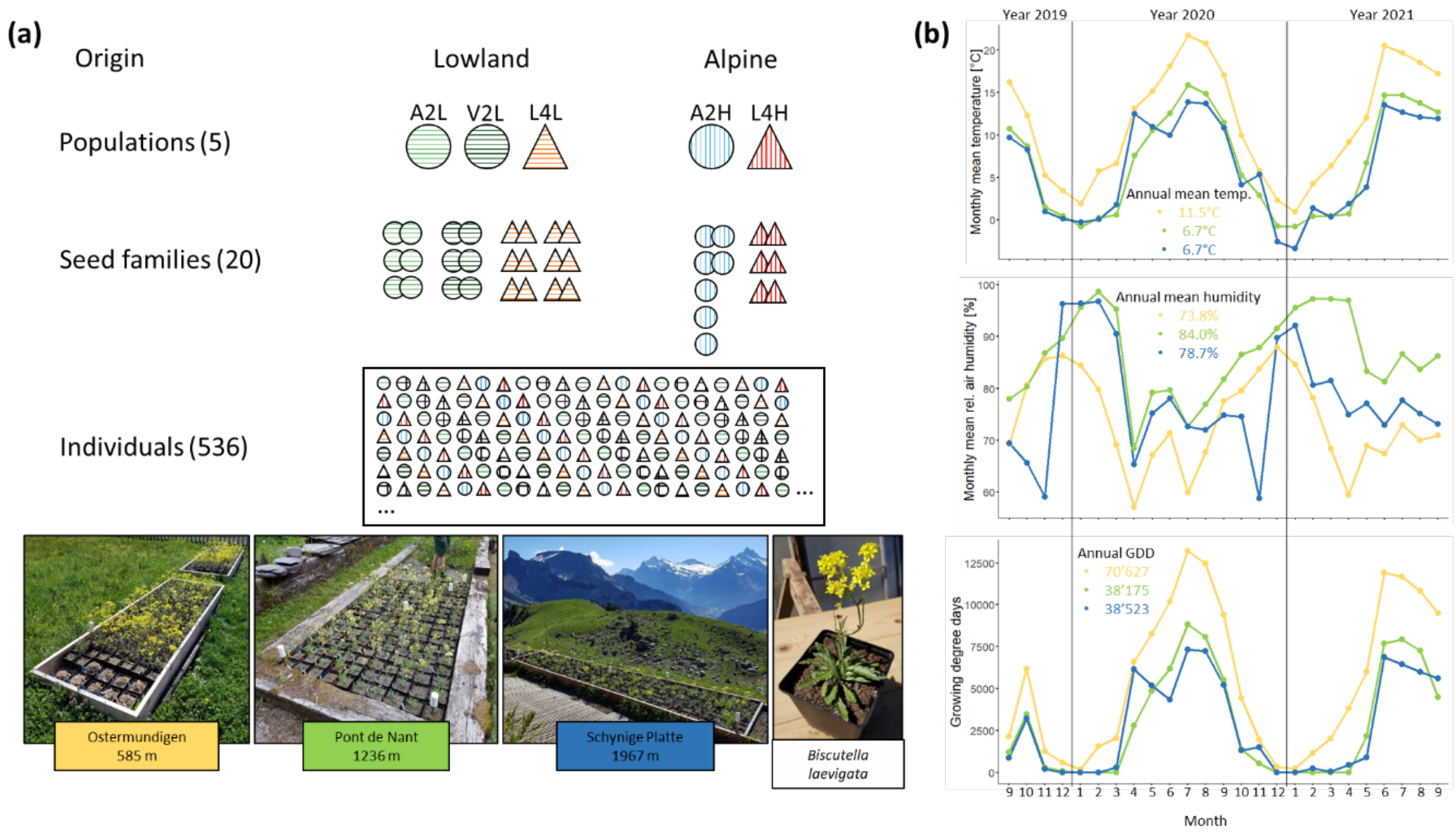

Fig. S7 – Design of the transplant experiment in *Biscutella laevigata*

Design of the transplant experiment into multiple common gardens across three elevation levels. (a) Three to six individuals of diploid (circles) and tetraploid (triangles) populations from low elevation (horizontal line pattern) and high elevation (vertical line pattern) were reciprocally crossed to generate reciprocal seed families for transplantation. Families consisted of ca. 30 individuals each and were distributed equally among the three elevation levels. A total of 536 individuals were transplanted across the three elevation levels, in a plot layout to maximize mixing of different populations and families. (b) Environmental data collected throughout the 768 days of the experiment, showing monthly mean temperature and relative air humidity, as well as the sum of growing degree days (GDD, i.e. cumulative heat > 4°C) per month in Ostermundigen at 585 m a.s.l. (yellow line), Pont de Nant at 1236 m a.s.l. (green) and Schynige Platte at 1967 m a.s.l. (blue).

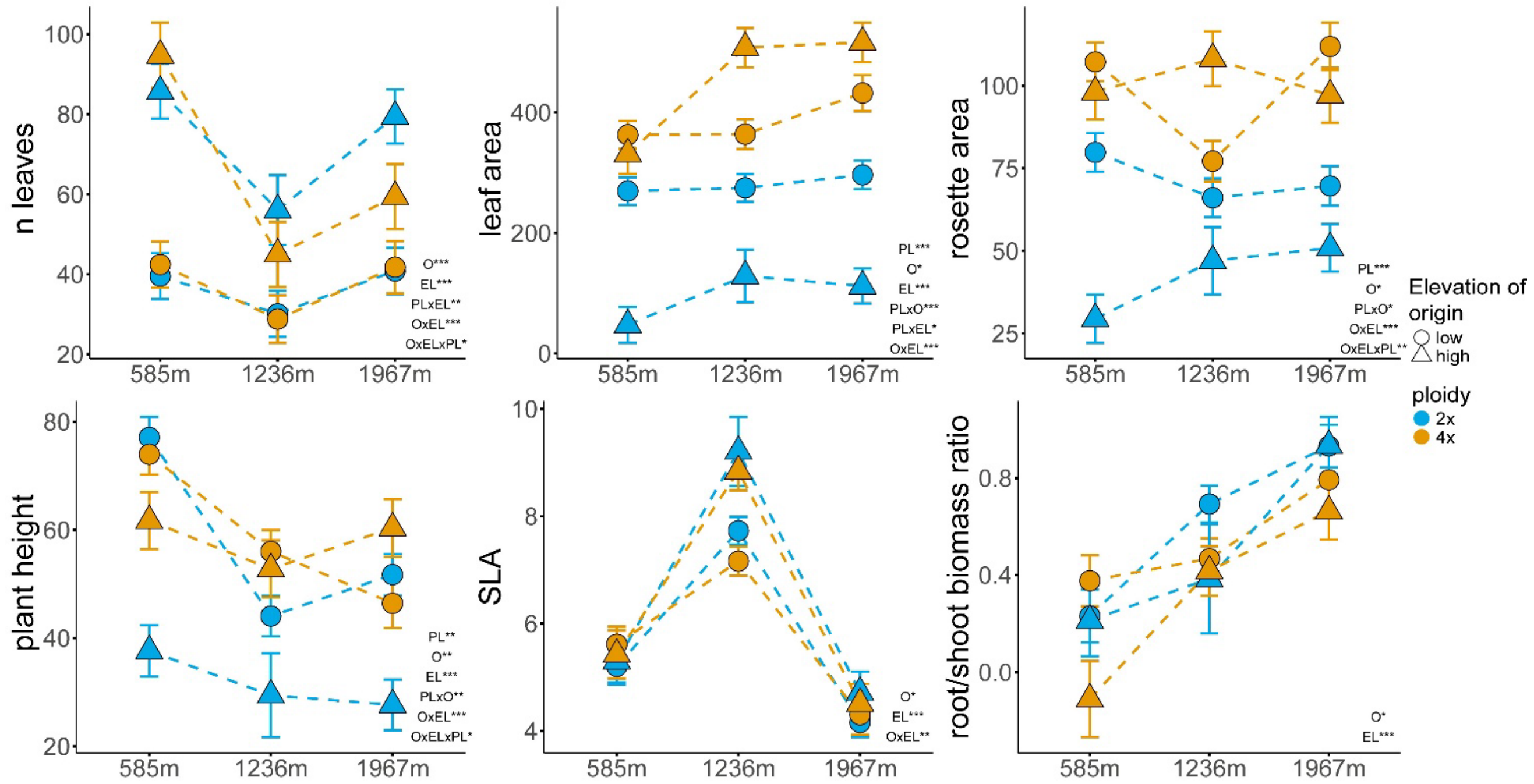

Fig. S8 – Phenotypes analysis in the transplant experiment

Phenotypes of diploids (2x) and tetraploids (4x) of *Biscutella laevigata* transplanted at three sites across elevation. Linear mixed models (Table 2), with estimated marginal means ±SE plotted across elevation and significant fixed effects shown as “Population origin” (O), “Elevation level” (EL), “Ploidy level” (PL) and their interactions. Plants from both ploidal levels differ in most phenotypes, with tetraploids showing larger leaves, larger rosettes and larger plant height across elevation. In contrast, number of leaves, specific leaf area, root and shoot biomass, as well as the root/shoot biomass ratio are mostly controlled by the environment and show no, or only marginal differences between ploidal levels. Significance levels: ***, $P < 0.001$; **, $P < 0.01$; *, $P < 0.05$.

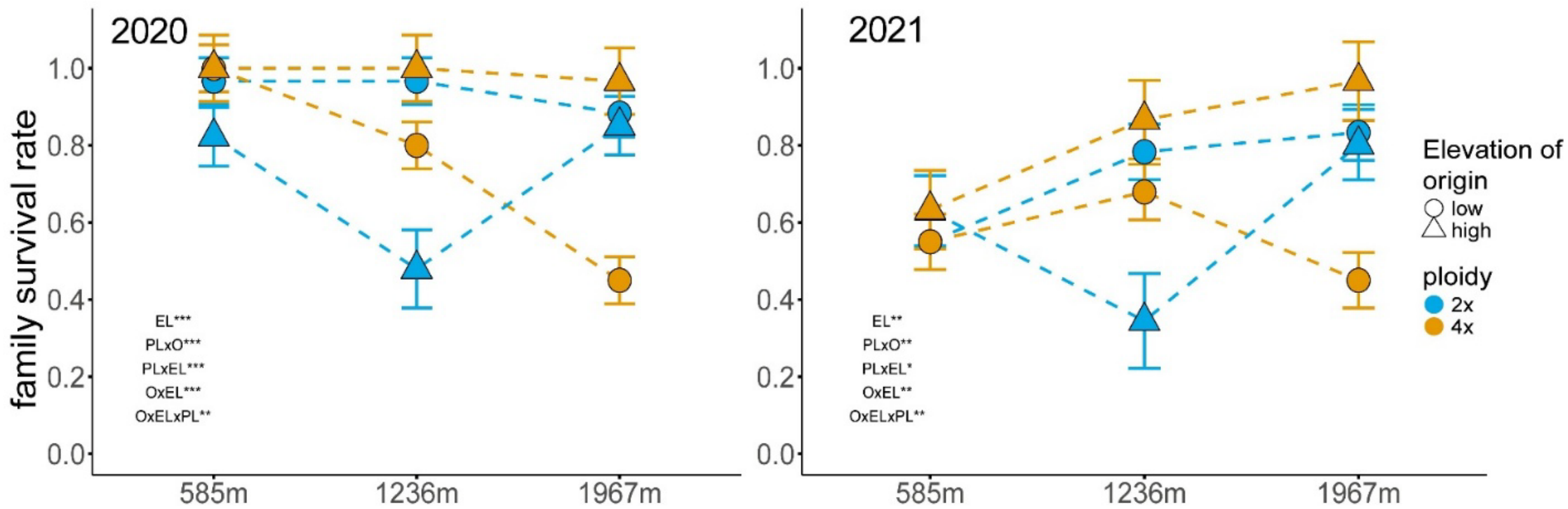

Fig. S9 – Survival rate in the transplant experiment
Survival rate of diploid (2x) and tetraploid (4x) of *Biscutella laevigata* from low and high elevation transplanted into three common gardens across elevation. Survival rate was quantified as the proportion of surviving plants per seed family during each growing season (i.e. 2020 and 2021). From 536 transplanted individuals, 461 plants survived the year 2020, while 360 individuals were still alive by the end of the experiment in September 2021. Significant fixed effects are depicted in the plots with the abbreviations for "Population origin" (O), "Elevation of growth" (EL), "Ploidy level" (PL) and their interactions. Estimated marginal means ±SE are plotted. Significance levels: ***, P < 0.001; **, P < 0.01; *, P < 0.05; ns, P > 0.05

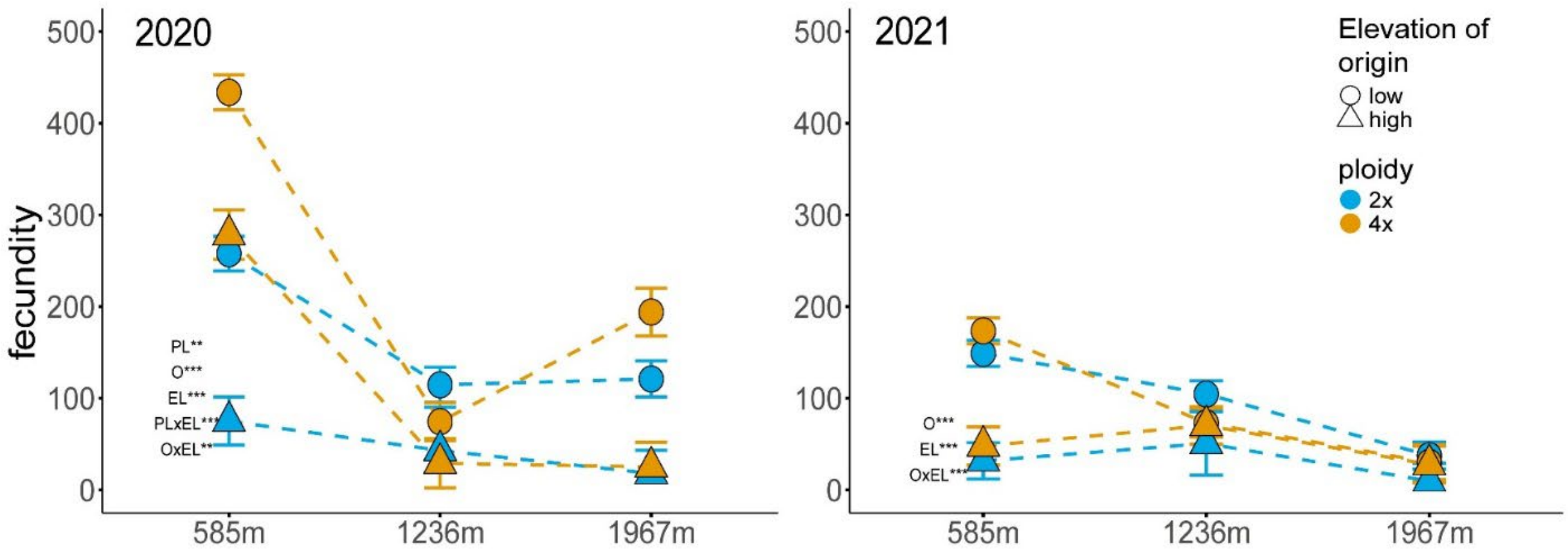

Fig. S10 – Fecundity in the transplant experiment
Fecundity of diploid (2x) and tetraploid (4x) of *Biscutella laevigata* from low and high elevation transplanted into three common gardens across elevation. Fecundity was estimated as the number of single-seed fruit per plant. Significant fixed effects are depicted in the plots with the abbreviations for "Population origin" (O), "Elevation of growth" (EL), "Ploidy level" (PL) and their interactions. Estimated marginal means ±SE are plotted. Significance levels: ***, P < 0.001; **, P < 0.01; *, P < 0.05; ns, P > 0.05

Table S1 – Genotyping of the 96 samples of *Biscutella laevigata*

Genotyping of the 96 samples of *Biscutella laevigata*, showing total number of reads across the corresponding .fq.gz files (total_reads), mean depth across all sites in the initial joint VCF file (DP0), mean depth at sites with DP>0 (DP1), mean depth considering sites with depth ≥ 4 in diploids and ≥ 8 in tetraploids (DPFilt), number of genotyped sites in the initial joint VCF file (NSITES.GENO), number of SNPs among NSITES.GENO (NSNP.GENO), number of sites with depth >0 (NSITES.DP1), and the number of sites with depth ≥ 4 in diploids and ≥ 8 in tetraploids (NSITES.DPFILT).

Sample-Alias_EMBL	Population	total_reads	DP0	DP1	DPFilt	NSITES.GENO	NSNP.GENO	NSITES.DP1	NSITES.DPFilt
A2-GX	DAG	115707972	15,76	20,67	22,32	147020323	15463240	112079174	103009226
A2-GA5	DAG	50124030	6,55	8,75	10,31	147020323	15463240	110029810	89380762
A2-GB13	DAG	40892934	5,46	7,45	9,03	147020323	15463240	107747273	83684373
A2-GB14	DAG	38937666	5,17	7,11	8,70	147020323	15463240	107018006	81606032
A2-GB16	DAG	45750146	5,84	7,90	9,46	147020323	15463240	108618481	86007598
A2-GB17	DAG	53125300	6,84	9,15	10,70	147020323	15463240	109920612	90490602
A2-GB9	DAG	44331958	5,65	7,76	9,32	147020323	15463240	107119637	84315706
A2-GC18	DAG	35592902	4,60	6,38	7,99	147020323	15463240	106150796	77510249
A2-Sch-12	DAS	62480394	8,12	10,45	11,98	147020323	15463240	114292149	96840783
A2-Sch-16	DAS	50676216	5,76	7,60	9,23	147020323	15463240	111319230	86361296
A2-Sch-17	DAS	84899636	10,48	13,28	14,86	147020323	15463240	116032885	101918222
A2-Sch-19	DAS	45873634	5,68	7,55	9,13	147020323	15463240	110666077	86102414
A2-Sch-21	DAS	44372802	5,73	7,57	9,09	147020323	15463240	111213676	87448042
A2-Sch-5	DAS	56271452	7,42	9,46	10,92	147020323	15463240	115342387	96587898
A2-Sch-8	DAS	35223032	4,79	6,47	8,07	147020323	15463240	108826523	80071020
A2-Sch-X	DAS	881466746	107,87	110,73	111,67	147020323	15463240	143225487	141992352
B2-MV1	DBM	23800938	2,78	4,47	6,41	147020323	15463240	91446767	51435321
B2-MV10	DBM	48386520	5,51	8,03	9,73	147020323	15463240	100803645	78791990
B2-MV18	DBM	58680942	6,68	9,51	11,25	147020323	15463240	103246709	83968560
B2-MV2	DBM	35729674	4,12	6,21	7,96	147020323	15463240	97509059	69097170
B2-MV20	DBM	71428206	7,73	10,85	12,66	147020323	15463240	104736287	87062721
B2-MV3	DBM	23431248	2,73	4,40	6,32	147020323	15463240	91286313	50931297
B2-MV6	DBM	64662580	7,01	9,94	11,73	147020323	15463240	103649953	84775351
B2-MV9	DBM	57144192	6,38	8,94	10,82	147020323	15463240	104979559	82879034
RCB-X	DPR	127088864	15,58	21,52	23,45	147020323	15463240	106430098	96894317
RCB-A16	DPR	44035878	5,14	7,48	9,16	147020323	15463240	101068390	77576521
RCB-B15	DPR	54075942	5,82	8,34	10,05	147020323	15463240	102570761	80964595
RCB-B17	DPR	52344302	6,12	8,70	10,40	147020323	15463240	103397991	82690393
RCB-B18	DPR	37295426	4,19	6,26	7,98	147020323	15463240	98289506	70235429
RCB-B19	DPR	72788126	7,29	10,25	12,01	147020323	15463240	104641044	86416120

RCB-C19	DPR	42923362	4,72	6,89	8,60	147020323	15463240	100578339	74710504
RCB-C8	DPR	60189384	6,68	9,45	11,18	147020323	15463240	103997413	84590445
K2-s2-X	DKS	129459362	17,00	23,73	25,66	147020323	15463240	105318824	96783065
K2-s2-12	DKS	37451190	4,77	7,06	8,64	147020323	15463240	99444755	75905848
K2-s2-3	DKS	74220150	9,03	12,60	14,35	147020323	15463240	105390681	90602441
K2-s2-4	DKS	36163638	4,44	6,59	8,22	147020323	15463240	99006243	73321177
K2-s2-9	DKS	53540346	6,32	9,09	10,72	147020323	15463240	102230024	83287668
K2-s2-A12	DKS	65890694	8,22	11,52	13,21	147020323	15463240	104839147	89154339
K2-s2-A17	DKS	64747136	7,51	10,65	12,34	147020323	15463240	103674155	86872149
K2-s2-A5	DKS	71034658	8,52	11,93	13,65	147020323	15463240	105038133	89625621
V2-bX	DVB	121908006	16,06	22,17	24,07	147020323	15463240	106482119	97354636
V2-b11	DVB	43326016	5,28	7,63	9,25	147020323	15463240	101765989	79168772
V2-b18	DVB	47099600	6,01	8,59	10,22	147020323	15463240	102880045	82655429
V2-b19	DVB	35317758	4,24	6,26	7,94	147020323	15463240	99607416	71445863
V2-b20	DVB	49218084	5,45	7,85	9,51	147020323	15463240	102072326	79629973
V2-b3	DVB	42163644	5,14	7,51	9,12	147020323	15463240	100667023	78020716
V2-bA4	DVB	46018318	5,81	8,32	9,94	147020323	15463240	102670394	81855747
V2-bA6	DVB	47830176	6,02	8,56	10,19	147020323	15463240	103368688	82997048
BUC-10	TBU	48644416	2,60	4,00	9,67	147020323	15463240	95601254	9455427
BUC-15	TBU	69167118	7,22	9,54	13,63	147020323	15463240	111258210	65087048
BUC-16	TBU	82988618	9,12	11,81	15,94	147020323	15463240	113570419	75190790
BUC-18	TBU	117267494	13,30	16,66	21,07	147020323	15463240	117403967	87550078
BUC-3	TBU	107428838	11,92	15,04	19,41	147020323	15463240	116562712	84129068
BUC-5	TBU	115678226	12,70	15,95	20,35	147020323	15463240	117082056	86088479
BUC-7	TBU	103922996	10,97	13,97	18,31	147020323	15463240	115443810	81184162
BUC-9	TBU	73074188	3,97	5,63	10,34	147020323	15463240	103706746	28802751
FUL-1	TFU	123200438	14,12	18,30	22,82	147020323	15463240	113441924	86698954
FUL-10	TFU	91714696	5,65	7,92	11,98	147020323	15463240	104919995	52415435
FUL-11	TFU	103537254	12,35	16,04	20,39	147020323	15463240	113184592	83793025
FUL-14	TFU	106337136	9,81	12,94	17,11	147020323	15463240	111494675	76904013
FUL-3	TFU	78131628	4,69	6,78	11,16	147020323	15463240	101708574	40069207
FUL-4	TFU	67135824	7,74	10,46	14,44	147020323	15463240	108711034	68396980
FUL-7	TFU	53356282	4,78	6,82	11,13	147020323	15463240	103053244	41585766
FUL-9	TFU	51010196	2,92	4,53	9,84	147020323	15463240	94908872	14203864
NAY-Aa3	TNA	100725430	11,92	14,93	19,26	147020323	15463240	117441471	84671689
NAY-Cb4	TNA	105231972	12,40	15,54	19,88	147020323	15463240	117342733	85706272
NAY-Da2	TNA	87257320	4,97	6,82	11,29	147020323	15463240	107065330	42600478
NAY-Fa18	TNA	76213676	4,34	6,07	10,64	147020323	15463240	105185014	34179867
NAY-Ha7	TNA	78440018	8,80	11,23	15,36	147020323	15463240	115179725	74186749

NAY-Ic2	TNA	117674574	13,69	17,05	21,50	147020323	15463240	118085666	88495430
NAY-Lc5	TNA	120882422	12,29	15,35	19,82	147020323	15463240	117720892	84981583
NAY-Z19	TNA	73201418	8,53	10,87	15,02	147020323	15463240	115473798	72870125
PRE-10	TPR	95913632	5,82	7,75	11,99	147020323	15463240	110386301	52626475
PRE-11	TPR	145327070	16,18	19,79	24,36	147020323	15463240	120218372	93577393
PRE-2	TPR	104155342	12,63	15,70	19,99	147020323	15463240	118276142	87034078
PRE-4	TPR	206363492	22,81	27,37	32,25	147020323	15463240	122498762	101593525
PRE-5	TPR	224680392	26,11	31,25	36,27	147020323	15463240	122825146	103926708
PRE-7	TPR	65024136	7,32	9,49	13,57	147020323	15463240	113409942	66046855
PRE-8	TPR	158685218	18,78	22,80	27,47	147020323	15463240	121070888	97297068
PRE-9	TPR	309903894	33,14	39,12	44,37	147020323	15463240	124541547	108500933
RAM-1	TRA	86700274	9,93	12,25	16,40	147020323	15463240	119108342	79915380
RAM-11	TRA	170579058	18,12	21,47	25,98	147020323	15463240	124071427	98808412
RAM-15	TRA	118246456	13,00	15,65	19,95	147020323	15463240	122135185	89608880
RAM-16	TRA	176335948	18,93	22,35	26,89	147020323	15463240	124489783	100043903
RAM-18	TRA	144850262	17,18	20,35	24,80	147020323	15463240	124120690	97733382
RAM-20	TRA	153027098	16,43	19,65	24,06	147020323	15463240	122920122	96169582
RAM-3	TRA	115637898	13,56	16,31	20,62	147020323	15463240	122227596	90880648
RAM-7	TRA	103672954	11,23	13,68	17,96	147020323	15463240	120670410	84182692
s3-11	TDS	163256620	19,08	23,57	28,37	147020323	15463240	119013537	95877568
s3-12	TDS	67872164	3,81	5,49	10,29	147020323	15463240	102047112	26437750
s3-13	TDS	60628396	3,51	5,12	10,08	147020323	15463240	100837336	21798340
s3-3	TDS	127959172	14,73	18,53	23,08	147020323	15463240	116914460	89445182
s3-5	TDS	146549540	16,95	21,17	25,92	147020323	15463240	117719731	92554964
s3-6	TDS	178805742	20,71	25,44	30,43	147020323	15463240	119683401	97353241
s3-7	TDS	231311084	24,94	30,48	35,62	147020323	15463240	120281200	100979641
s3-8	TDS	85314788	4,79	6,67	11,12	147020323	15463240	105723835	40704276

Table S2 – Genotyping in the twelve populations of *Biscutella laevigata*

Genotyping in the twelve populations of *Biscutella laevigata*, showing the averaged depth per population (DP) after filtering for a minimal genotype depth of 4 for diploids and 8 for tetraploids and the number of corresponding sites (Sites) with less than 30% of missing data.

Pop	Ploidy	Elevation	DP	Sites
DVB	2x	Low	11.3	90850295
DKS	2x	Low	13.3	95883964
DAG	2x	Low	11.0	95229693
DBM	2x	High	9.6	83490238
DPR	2x	High	11.6	91050620
DAS	2x	High	10.5	104382090
TFU	4x	Low	14.9	68187254
TBU	4x	Low	16.1	78794295
TPR	4x	Low	26.3	102187609
TNA	4x	High	16.6	81864129
TDS	4x	High	21.9	89588269
TRA	4x	High	22.1	102172830

Table S3 – Differentiation at synonymous sites between pairs of populations

Differentiation at synonymous 4dg sites between population pairs (Pop1-Pop2) within diploid and tetraploid *Biscutella laevigata*. Three pairs of low- vs. high-elevation populations per ploidal level (marked with *) were used in genome scans to investigate adaptation to elevation between ploidal levels (i.e. Ploidy, 2x = diploid and 4x = tetraploid populations).

**Pop1**	**Pop2**	**Ploidy**	**Elevation**[†]	**Rho**	**F_{ST}**	**d_{XY}**	**AFD**[¶]
DAG*	DAS*	2x	delta	0.1129	0.0737	0.0117	0.0071
DAG	DBM	2x	delta	0.2287	0.1865	0.0145	0.0109
DAG	DPR	2x	delta	0.2941	0.2778	0.0145	0.0120
DKS	DAS	2x	delta	0.3250	0.3294	0.0138	0.0114
DKS	DBM	2x	delta	0.3254	0.3295	0.0151	0.0128
DKS*	DPR*	2x	delta	0.3733	0.4341	0.0157	0.0140
DVB	DAS	2x	delta	0.3047	0.2925	0.0141	0.0115
DVB*	DBM*	2x	delta	0.2899	0.2671	0.0149	0.0120
DVB	DPR	2x	delta	0.3544	0.3884	0.0157	0.0138
DAG	DKS	2x	low	0.2908	0.2706	0.0135	0.0106
DAG	DVB	2x	low	0.2748	0.2458	0.0140	0.0109
DVB	DKS	2x	low	0.3029	0.2839	0.0126	0.0099
DAS	DBM	2x	high	0.2509	0.2136	0.0142	0.0110
DAS	DPR	2x	high	0.3053	0.2954	0.0140	0.0117
DBM	DPR	2x	high	0.2897	0.2684	0.0147	0.0121
TBU*	TDS*	4x	delta	0.1795	0.0791	0.0119	0.0068
TBU	TNA	4x	delta	0.2034	0.0991	0.0128	0.0080
TBU	TRA	4x	delta	0.2589	0.1409	0.0139	0.0095
TFU	TDS	4x	delta	0.2395	0.1222	0.0115	0.0075
TFU*	TNA*	4x	delta	0.1824	0.0837	0.0116	0.0068
TFU	TRA	4x	delta	0.3086	0.1960	0.0139	0.0105
TDS	TPR	4x	delta	0.2607	0.1403	0.0131	0.0088
TNA	TPR	4x	delta	0.2500	0.1352	0.0135	0.0092
TPR*	TRA*	4x	delta	0.2459	0.1268	0.0140	0.0091
TBU	TPR	4x	low	0.2396	0.1238	0.0127	0.0083
TFU	TBU	4x	low	0.2427	0.1284	0.0115	0.0077
TFU	TPR	4x	low	0.2997	0.1851	0.0126	0.0093
TDS	TRA	4x	high	0.2714	0.1507	0.0142	0.0099
TNA	TDS	4x	high	0.2075	0.1000	0.0129	0.0081
TNA	TRA	4x	high	0.2177	0.1070	0.0140	0.0088

[†] delta indicates pairs in which Pop1 is at low elevation and Pop2 at high elevation, low indicates pairs in which both populations are at low elevation, and high indicates pairs in which both populations are at high elevation

[¶]Allelic frequency difference (AFD)

Table S4 – Summary of genome scans between pairs of populations

Number of genomic windows (W) and included genes (G) analyzed and presenting signatures of adaptation to elevation in pairs of diploid and tetraploid *Biscutella laevigata* populations. Candidate loci were identified as Rho and D_{XY} outliers (above 3rd quartile + 1.5 interquartile range of genome-wide values) or as selective sweeps through pi~AFD residuals (below 1st quartile – 1.5 interquartile range of genome-wide values). Genes overlapping with candidate loci were prioritized using per-SNP scans and keeping only candidate genes (CG) including at least one outlier SNP (SNPs)

Pair of populations				Analyzed				Rho outlier		Rho and d_{XY} outlier			Sweep low[#]			Sweep high[#]			Sweep both[#]			Enriched GO terms[∇]
low	high	Ploidy	Rho	W	G	%G*	SNPs	W	SNPs	W	G	CG	W	G	CG	W	G	CG	W	G	CG	
DAG	DAS	2x	0.139	13285	40037	95,1	3086440	259	101275	59	30	13	58	65	30	37	61	24	58	33	15	<u>Rho and d_{XY} outliers</u><br>Metabolism; protein processing; stress and defense responses
DKS	DPR	2x	0.382	12396	39420	93,6	2622522	0	0	0	0	0	33	20	0	45	14	0	18	9	0	
DVB	DBM	2x	0,304	12286	39191	93,1	2777999	0	27993	0	0	0	46	39	0	42	10	0	30	13	4	<u>Sweeps</u><br>anthocyanin-containing compound biosynthesis; defense response; lysine biosynthesis; response to toxic substance
TFU	TNA	4x	0.198	11591	38207	90,7	2674821	25	43735	5	4	3	28	25	10	37	67	34	45	22	13	<u>Rho and d_{XY} outliers</u><br>Development and reproduction, stress and signaling responses, metabolism, gene regulation, mitochondrial respiration
TBU	TDS	4x	0.173	12467	39183	93,0	3368484	95	184583	13	39	28	56	93	47	51	47	19	38	94	53	
TPR	TRA	4x	0.241	13412	40327	95,8	5208073	45	147715	7	8	5	53	56	30	58	55	27	29	25	10	<u>Sweeps</u><br>ion transport, signal transduction, gene expression regulation, meiotic gene conversion, defense response, negative regulation of shoot apical meristem development, floral organ formation, negative regulation of reciprocal meiotic recombination, NADH metabolic process

* proportion of annotated gene models overlapping with analyzed windows

[¶]Loci identified as outlier for both rho and d_{XY}, supporting reduced gene flow by selection

[#]Loci showing excessive differentiation relative to their diversity, supporting a selecting sweep in the low elevation, the high elevation population, or both (shared loci are not included in population-specific counts).

[∇]Gene Ontology (GO) terms being significantly enriched, see Table S6 and Table S8 for exhaustive lists

Table S5 – Candidate genes underlying adaptation to elevation in diploids

Candidate genes underlying adaptation to elevation in diploid populations of *Biscutella laevigata* and their putative functions based on orthologs in *Arabidopsis thaliana*.

Gene	Pair and type	Ortholog	Description TAIR10
BLAEV0051342	DAG-DAS_sweep_pop2only	AT1G07220	O-glucosyltransferase rumi-like protein (DUF821);(source:Araport11)
BLAEV0033852	DAG-DAS_sweep_pop2only	AT1G21190	Small nuclear ribonucleoprotein family protein;(source:Araport11)
BLAEV0033853	DAG-DAS_sweep_pop2only	AT1G21200	sequence-specific DNA binding transcription factor;(source:Araport11)
BLAEV0033856	DAG-DAS_sweep_pop2only	AT1G21250	Encodes a cell wall-associated kinase that interacts with AtGRP3 and may function as a signaling receptor of extracellular matrix component such as oligogalacturonides. The mRNA is cell-to-cell mobile.
BLAEV0033858	DAG-DAS_sweep_pop2only	AT1G21310	Encodes extensin 3.
BLAEV0034480	DAG-DAS_rhodxy, DAG-DAS_sweep_pop2only	AT1G28160	Encodes a member of the ERF (ethylene response factor) subfamily B-1 of ERF/AP2 transcription factor family. The protein contains one AP2 domain. There are 15 members in this subfamily including ATERF-3; ATERF-4, ATERF-7, and leafy petiole.
BLAEV0029132	DAG-DAS_sweep_pop2only	AT1G28480	Encodes GRX480; a member of the glutaredoxin family that regulates protein redox state. GRX480 interacts with TGA factors and suppresses JA-responsive PDF1.2 transcription. GRX480 transcription is SA-inducible and requires NPR1. Maybe involved in SA/JA cross-talk. It has also been shown to interact with the transcription factor TGA2 and suppress ORA59 promoter activity.
BLAEV0028389	DAG-DAS_sweep_pop1only	AT1G54780	Encodes a thylakoid lumen protein regulating photosystem II repair cycle. Has acid phosphatase activity. The mRNA is cell-to-cell mobile.
BLAEV0028388	DAG-DAS_sweep_pop1only	AT1G54790	GDSL-motif esterase/acyltransferase/lipase. Enzyme group with broad substrate specificity that may catalyze acyltransfer or hydrolase reactions with lipid and non-lipid substrates.
BLAEV0028471	DAG-DAS_rhodxy	AT1G65020	plasma protein;(source:Araport11)
BLAEV0014062	DVB-DBM_sweep_pop1only	AT2G17710	Big1;(source:Araport11)
BLAEV0044439	DAG-DAS_sweep_both	AT2G17850	Rhodanese/Cell cycle control phosphatase superfamily protein;(source:Araport11)
BLAEV0044249	DAG-DAS_sweep_pop2only	AT2G19570	Encodes a cytidine deaminase that deaminates cytidine and deoxycytidine and is competitively inhibited by cytosine-containing compounds.
BLAEV0004140	DAG-DAS_sweep_pop2only	AT2G27170	Encodes a member of the Arabidopsis cohesin complex that is essential for viability and sister chromatid alignment.
BLAEV0004200	DVB-DBM_sweep_pop1only	AT2G27580	Regulated by heat shock.
BLAEV0004357	DVB-DBM_sweep_both	AT2G28830	Encodes a U-box E3 ubiquitin ligase involved in ubiquitination of pattern recognition receptor FLS2.pub12/pub13 double mutants enhanced chitin-induced ROS production and callose deposition suggesting they function redundantly to negatively regulate immune response to fungal elicitor.
BLAEV0003869	DAG-DAS_sweep_pop1only	AT2G28960	Leucine-rich repeat protein kinase family protein;(source:Araport11)
BLAEV0002502	DAG-DAS_sweep_pop2only	AT2G39630	Encodes a putative dolichyl-phosphate β-glucosyltransferase.

BLAEV0006481	DAG-DAS_sweep_pop2only	AT3G23740	hypothetical protein;(source:Araport11)
BLAEV0013573	DAG-DAS_sweep_pop2only	AT3G29670	Encodes a malonyltransferase that may play a role in phenolic xenobiotic detoxification. The mRNA is cell-to-cell mobile.
BLAEV0049915	DVB-DBM_sweep_pop1only	AT3G59410	Encodes an eIF2alpha kinase that can bind uncharged tRNA via its C-terminus and can phosphorylate both eIF2alpha homologues in Arabidopsis.
BLAEV0015578	DAG-DAS_sweep_pop1only	AT4G00650	Encodes a major determinant of natural variation in Arabidopsis flowering time. Dominant alleles of FRI confer a vernalization requirement causing plants to overwinter vegetatively. Many early flowering accessions carry loss-of-function fri alleles .Twenty distinct haplotypes that contain non-functional FRI alleles have been identified and the distribution analyzed in over 190 accessions. The common lab strains- Col and Ler each carry loss of function mutations in FRI.
BLAEV0043882	DVB-DBM_sweep_both	AT4G13010	Oxidoreductase; zinc-binding dehydrogenase family protein;(source:Araport11)
BLAEV0043884	DVB-DBM_sweep_pop1only	AT4G13070	RNA-binding CRS1 / YhbY (CRM) domain protein;(source:Araport11)
BLAEV0043892	DVB-DBM_sweep_pop1only	AT4G13075	Rapid alkalinization factor (RALF) family protein. Member of a diversely expressed predicted peptide family showing sequence similarity to tobacco Rapid Alkalinization Factor (RALF); and is believed to play an essential role in the physiology of Arabidopsis. Consists of a single exon and is characterized by a conserved C-terminal motif and N-terminal signal peptide.
BLAEV0021215	DAG-DAS_sweep_both	AT4G13480	Member of the R2R3 factor gene family.
BLAEV0031255	DAG-DAS_sweep_pop1only	AT4G13980	member of Heat Stress Transcription Factor (Hsf) family
BLAEV0031253	DAG-DAS_sweep_pop1only	AT4G14010	Member of a diversely expressed predicted peptide family showing sequence similarity to tobacco Rapid Alkalinization Factor (RALF); and is believed to play an essential role in the physiology of Arabidopsis. Consists of a single exon and is characterized by a conserved C-terminal motif and N-terminal signal peptide.
BLAEV0021201	DAG-DAS_sweep_both	AT5G05340	Encodes a protein with sequence similarity to peroxidases that is involved in lignin biosynthesis. Loss of function mutations show abnormal development of xylem fibers and reduced levels of lignin biosynthetic enxymes.
BLAEV0015041	DAG-DAS_sweep_pop1only	AT5G07870	HXXXD-type acyl-transferase family protein;(source:Araport11)
BLAEV0017250	DAG-DAS_sweep_pop1only	AT5G20380	Encodes an inorganic phosphate transporter (PHT4;5).
BLAEV0010926	DAG-DAS_sweep_pop1only	AT5G20940	Glycosyl hydrolase family protein;(source:Araport11)
BLAEV0017091	DAG-DAS_sweep_pop2only	AT5G22110	Encodes a protein with similarity to DNA polymerase epsilon subunit B an essential gene that is required for DNA replication. Homozygous mutants are embryo lethal. Expressed in meristematic ; rapidly dividing regions.
BLAEV0043780	DVB-DBM_sweep_both	AT5G36120	One of four Arabidopsis homologs of bacterial ymlg proteins.
BLAEV0015579	DAG-DAS_sweep_pop1only	AT5G51090	hypothetical protein;(source:Araport11)
BLAEV0030885	DVB-DBM_sweep_both	AT5G51950	Glucose-methanol-choline (GMC) oxidoreductase family protein;(source:Araport11)

BLAEV0015753	DVB-DBM_sweep_pop1only	AT5G52240	Encodes a protein with similarity to progesterone-binding proteins in animals. Has been shown to bind steroids in vitro. Expressed in aerial portions of the plant excluding mature flowers and siliques. Antisense experiments suggest a role in inhibition of hypocotyl cell elongation. Expression is suppressed light grown seedlings transferred to the dark. The mRNA is cell-to-cell mobile.
BLAEV0015859	DAG-DAS_sweep_pop1only	AT5G53200	Encodes a R3MYB transcription inhibitor that regulates trichome patterning. Mutants produce trichome clusters whereas other transcriptional inhibitors involved in this patterning are involved in trichome density regulation. Natural hypofunctional alleles producing trichome development in fruits have been found.
BLAEV0015868	DAG-DAS_sweep_pop1only, DVB-DBM_sweep_pop2only	AT5G53280	An integral outer envelope membrane protein (as its homolog PDV2); component of the plastid division machinery. Similar to ARC5, PDV1 localized to a discontinuous ring at the division site in wild-type plants. PDV1 and PDV2 are required for localization of ARC5 at the chloroplast division site. Topological analysis showed that the large N-terminal region of PDV1 upstream of the transmembrane helix bearing a putative coiled-coil domain is exposed to the cytosol. Mutation of the conserved PDV1 C-terminal Gly residue did not block PDV1 insertion into the outer envelope membrane but did abolish its localization to the division site. The mRNA is cell-to-cell mobile.
BLAEV0037245	DAG-DAS_sweep_pop1only	AT5G64780	holocarboxylase synthetase;(source:Araport11)
BLAEV0000660	DAG-DAS_rhodxy, DAG-DAS_sweep_both		
BLAEV0003868	DAG-DAS_sweep_pop1only		
BLAEV0004284	DAG-DAS_rhodxy, DAG-DAS_sweep_both		
BLAEV0004582	DAG-DAS_sweep_pop1only		
BLAEV0004583	DAG-DAS_sweep_pop1only		
BLAEV0006153	DAG-DAS_rhodxy, DAG-DAS_sweep_both		
BLAEV0006154	DAG-DAS_rhodxy, DAG-DAS_sweep_both		
BLAEV0006155	DAG-DAS_rhodxy, DAG-DAS_sweep_both		
BLAEV0012613	DAG-DAS_sweep_pop1only		
BLAEV0013574	DAG-DAS_rhodxy, DAG-DAS_sweep_both		
BLAEV0013576	DAG-DAS_rhodxy, DAG-DAS_sweep_both		
BLAEV0014053	DAG-DAS_sweep_pop1only		
BLAEV0014054	DAG-DAS_sweep_pop1only		
BLAEV0015032	DAG-DAS_rhodxy, DAG-DAS_sweep_both		
BLAEV0015270	DVB-DBM_sweep_pop2only		

BLAEV0015519	DAG-DAS_sweep_pop2only
BLAEV0015542	DAG-DAS_sweep_pop1only
BLAEV0017090	DAG-DAS_sweep_pop2only
BLAEV0017092	DAG-DAS_sweep_pop2only
BLAEV0020891	DAG-DAS_sweep_pop1only
BLAEV0021209	DAG-DAS_rhodxy, DAG-DAS_sweep_pop2only
BLAEV0021211	DAG-DAS_rhodxy, DAG-DAS_sweep_both
BLAEV0021274	DAG-DAS_sweep_pop1only
BLAEV0021515	DAG-DAS_sweep_pop2only
BLAEV0021683	DVB-DBM_sweep_pop1only
BLAEV0031254	DAG-DAS_sweep_pop1only
BLAEV0033851	DAG-DAS_sweep_pop2only
BLAEV0033854	DAG-DAS_sweep_pop2only
BLAEV0036417	DVB-DBM_sweep_pop1only
BLAEV0037242	DAG-DAS_sweep_pop1only
BLAEV0037255	DAG-DAS_sweep_pop1only
BLAEV0037256	DAG-DAS_sweep_pop1only
BLAEV0039815	DAG-DAS_sweep_pop2only
BLAEV0039816	DAG-DAS_sweep_pop2only
BLAEV0040068	DAG-DAS_sweep_both
BLAEV0047118	DAG-DAS_sweep_pop1only
BLAEV0047120	DAG-DAS_sweep_pop1only
BLAEV0049911	DVB-DBM_sweep_pop1only
BLAEV0051652	DAG-DAS_sweep_both
BLAEV0051953	DAG-DAS_sweep_pop2only
BLAEV0051954	DAG-DAS_sweep_pop2only
BLAEV0015442	DAG-DAS_sweep_pop1only
BLAEV0000662	DAG-DAS_rhodxy, DAG-DAS_sweep_both
BLAEV0037253	DAG-DAS_sweep_pop1only

Table S6 – Gene ontology terms significantly enriched in diploids
Gene ontology terms significantly enriched in diploids of *Biscutella laevigata*.

Type	GO.ID	Term	Annotated	Significant	Expected	topgoFisher
Rho and d_{XY}	GO:0009089	lysine biosynthetic process via diaminopimelate	22	3	0.01	3.10E-08
	GO:0009718	anthocyanin-containing compound biosynthetic process	87	2	0.03	0.0003
	GO:0032527	protein exit from endoplasmic reticulum	12	1	0	0.0036
	GO:2000031	regulation of salicylic acid mediated signaling pathway	28	1	0.01	0.0084
	GO:0009636	response to toxic substance	558	2	0.17	0.0116
sweep	GO:0009089	lysine biosynthetic process via diaminopimelate	22	3	0.04	1.10E-05
	GO:0009636	response to toxic substance	558	5	1.11	5.30E-05
	GO:0009718	anthocyanin-containing compound biosynthetic process	87	3	0.17	0.00071
	GO:0009972	cytidine deamination	2	1	0	0.00397
	GO:0052696	flavonoid glucuronidation	3	1	0.01	0.00595
	GO:0009862	systemic acquired resistance, salicylic acid mediated signaling pathway	197	3	0.39	0.00717
	GO:0010321	regulation of vegetative phase change	5	1	0.01	0.0099
	GO:0032922	circadian regulation of gene expression	6	1	0.01	0.01186
	GO:0042276	error-prone translesion synthesis	7	1	0.01	0.01383
	GO:0071722	detoxification of arsenic-containing substance	8	1	0.02	0.01579
	GO:0010190	cytochrome b6f complex assembly	11	1	0.02	0.02165
	GO:0007267	cell-cell signaling	194	3	0.39	0.02232
	GO:0006521	regulation of cellular amino acid metabolic process	12	1	0.02	0.02359
	GO:0032527	protein exit from endoplasmic reticulum	12	1	0.02	0.02359
	GO:0019722	calcium-mediated signaling	122	2	0.24	0.02466
	GO:0010206	photosystem II repair	13	1	0.03	0.02553
	GO:0010430	fatty acid omega-oxidation	14	1	0.03	0.02747
	GO:0048629	trichome patterning	17	1	0.03	0.03326
	GO:0030154	cell differentiation	1622	7	3.22	0.03468
	GO:0010310	regulation of hydrogen peroxide metabolic process	150	2	0.3	0.03604
	GO:0030308	negative regulation of cell growth	20	1	0.04	0.03901
	GO:0019348	dolichol metabolic process	22	1	0.04	0.04283
	GO:0015706	nitrate transmembrane transport	170	2	0.34	0.04517
	GO:0043069	negative regulation of programmed cell death	170	2	0.34	0.04517
	GO:0006664	glycolipid metabolic process	213	2	0.42	0.04624

Table S7 – Candidate genes underlying adaptation to elevation in tetraploids

Candidate genes underlying adaptation to elevation in tetraploid populations of *Biscutella laevigata* and their putative functions based on orthologs in *Arabidopsis thaliana*.

Gene	Pair and type	Ortholog	Description TAIR10
BLAEV0009078	TPR-TDS_sweep_pop2only	AT1G05120	One of two paralogs of yeast RAD1; the other being At1g02670. Both are expressed throughout plant growth with RAD16 as the dominant transcript. Mutants show increased sensitivity to UV.
BLAEV0051464	TPR-TDS_sweep_pop1only	AT1G05240	Class III peroxidase; controls together with PRX44 and PRX37 extensin-mediated cell wall properties during polar expansion of root hair cells.
BLAEV0051459	TBU-TDS_sweep_both, TPR-TDS_sweep_pop1only	AT1G05260	Encodes a cold-inducible cationic peroxidase that is involved in the stress response. In response to low temperature; RCI3 transcripts accumulate in the aerial part and in roots of etiolated seedlings but only in roots of light-grown seedlings. The mRNA is cell-to-cell mobile.
BLAEV0004449	TPR-TNA_sweep_pop2only	AT1G07400	HSP20-like chaperones superfamily protein;(source:Araport11)
BLAEV0028258	TFU-TNA_sweep_pop2only	AT1G09590	Translation protein SH3-like family protein;(source:Araport11)
BLAEV0034857	TBU-TRA_sweep_pop1only	AT1G15550	Involved in later steps of the gibberellic acid biosynthetic pathway. Activated by AGAMOUS in a cal-1; ap1-1 background. Deletion of 208 bp from -1016 to -809 (Δ-808) resulted in loss of GA-negative feedback (this sequence, which contains a 43-bp sequence GNFEI, was shown to be sufficient for GA-negative feedback).
BLAEV0035184	TBU-TRA_sweep_pop1only	AT1G18485	Pentatricopeptide repeat (PPR) superfamily protein;(source:Araport11)
BLAEV0029450	TBU-TDS_sweep_pop2only, TBU-TNA_sweep_pop2only	AT1G19835	TCS1 encodes a coiled-coil domain protein that binds to microtubules and co-localizes with the cortical microtubules. Mutants have defects in trichome branching and hypocotyl elongation. TCS1 interacts with ZWI and appears to be involved in microtubule assembly.
BLAEV0029381	TPR-TRA_sweep_pop1only	AT1G26160	Metal-dependent phosphohydrolase;(source:Araport11)
BLAEV0029928	TFU-TDS_sweep_both	AT1G26780	Encodes LOF1 (LATERAL ORGAN FUSION1); a MYB-domain transcription factor expressed in organ boundaries. Functions in boundary specification, meristem initiation and maintenance, and organ patterning. Also see LOF2 (At1g69560).
BLAEV0029922	TFU-TDS_sweep_pop2only	AT1G26790	Dof-type zinc finger DNA-binding family protein;(source:Araport11)
BLAEV0028907	TBU-TDS_sweep_pop1only	AT1G30135	jasmonate-zim-domain protein 8;(source:Araport11)
BLAEV0033489	TBU-TDS_sweep_both, TBU-TRA_sweep_pop1only, TBU-TPR_rhodxy, TBU-TPR_sweep_both	AT1G32540	Encodes a protein with 3 plant-specific zinc finger domains that acts as a positive regulator of cell death.
BLAEV0033486	TBU-TDS_sweep_both, TBU-TRA_sweep_pop1only, TBU-TPR_rhodxy, TBU-TPR_sweep_both	AT1G32550	Encodes FdC2; a ferredoxin protein capable of alternative electron partitioning. FdC1 level increases in conditions of acceptor limitation at PSI.
BLAEV0033433	TPR-TRA_sweep_pop2only	AT1G33080	MATE efflux family protein;(source:Araport11)
BLAEV0033434	TPR-TRA_sweep_pop2only	AT1G33120	Ribosomal protein L6 family;(source:Araport11)
BLAEV0028601	TBU-TNA_sweep_pop2only, TPR-TNA_rhodxy, TPR-TNA_sweep_both	AT1G33800	Encodes a glucuronoxylan(GX)-speciﬁc 4-O-methyltransferase responsible for methylating GlcA residues in GX. Reduced methylation of GX ingxmt1-1 plants is correlated with altered lignin composition. The mRNA is cell-to-cell mobile.
BLAEV0041479	TDS-TRA_sweep_pop1only	AT1G44000	STAY-GREEN-like protein;(source:Araport11)

BLAEV0041477	TDS-TRA_sweep_pop1only	AT1G44110	Cyclin A1;(source:Araport11)
BLAEV0041291	TPR-TDS_rhodxy, TPR-TDS_sweep_pop2only, TNA-TDS_sweep_pop2only, TDS-TRA_sweep_both	AT1G48370	Arabidopsis thaliana metal-nicotianamine transporter YSL4
BLAEV0041290	TPR-TDS_rhodxy, TPR-TDS_sweep_pop2only, TDS-TRA_sweep_both	AT1G48380	Encodes a novel nuclear protein required for root hair initiation and ploidy-dependent cell growth. Its sequence has similarity to the C-terminal domain of mammalian DNA topo IIalpha. Shows in vitro DNA binding activity and is likely to be part of the topo VI complex by binding to subunit A.
BLAEV0041032	TBU-TPR_sweep_pop1only	AT1G49730	Protein kinase superfamily protein;(source:Araport11)
BLAEV0040992	TPR-TRA_rhodxy, TPR-TRA_sweep_both	AT1G50030	TOR is a protein kinase; conserved across eukaryotes (called mTOR in mammals), that monitors nutrient availability to coordinate metabolism (PMID: 32649861). TOR is a member of the phosphatidylinositol 3-kinase-related kinases (PIKKs) and forms a stable complex (TOR complex 1, or TORC1) with RAPTOR (At3g08850, At5g01770, PMID: 16377759) and LST8 (At3g18140, At2g22040, PMID: 22307851). TOR is expressed throughout Arabidopsis development (PMID: 32188942, PMID: 32051250) and is required to complete embryogenesis (PMID: 11983923). Diverse nutrients, hormones, and environmental cues can activate or suppress TOR activity (depending on the physiological and developmental context), including sugars (PMID: 23542588), amino acids (PMID: 33831352, PMID: 35327579), nucleotides (PMID: 33793860), auxins (PMID: 28246118), and light (PMID: 28223530). When conditions are favorable, TOR promotes growth by activating cell division (PMID: 23542588), cell expansion (PMID: 33288701), ribosome biogenesis (PMID: 33054972), protein synthesis (PMID: 33054972), and nucleotide synthesis (PMID: 33793860), among other processes.
BLAEV0040982	TPR-TNA_sweep_pop2only	AT1G50140	P-loop containing nucleoside triphosphate hydrolases superfamily protein;(source:Araport11)
BLAEV0040924	TPR-TNA_sweep_pop2only	AT1G50690	Cystatin/monellin superfamily protein;(source:Araport11)
BLAEV0040923	TPR-TNA_sweep_pop2only	AT1G50690	Cystatin/monellin superfamily protein;(source:Araport11)
BLAEV0028341	TNA-TRA_sweep_pop2only	AT1G56210	Heavy metal transport/detoxification superfamily protein;(source:Araport11)
BLAEV0028261	TFU-TNA_sweep_pop2only	AT1G57650	ATP binding protein;(source:Araport11)
BLAEV0028232	TBU-TNA_sweep_pop1only	AT1G58025	A specific subunit of MINU1/2-associated SWI/SNF (MAS) complexes (PMID:36189880 and PMID:36471048).
BLAEV0027910	TBU-TDS_sweep_pop1only	AT1G58350	Putative serine esterase family protein;(source:Araport11)
BLAEV0027871	TFU-TNA_sweep_pop2only	AT1G59610	A high molecular weight GTPase whose GTP-binding domain shows a low homology to those of other plant dynamin-like proteins.Contains a pleckstrin homologous domain. DRP2B and DRP1A participate together in clathrin-coated vesicle formation during endocytosis. The mRNA is cell-to-cell mobile.
BLAEV0027859	TBU-TRA_sweep_both, TPR-TRA_sweep_pop2only, TDS-TRA_sweep_pop2only	AT1G59620	Encodes CW9.
BLAEV0027869	TFU-TNA_sweep_pop2only	AT1G59640	A basic helix-loop-helix encoding gene (BIGPETAL; BPE) involved in the control of petal size. BPE is expressed via two mRNAs derived from an alternative splicing event. The BPEub (AT1G59640.1)transcript is expressed ubiquitously, whereas the BPEp (AT1G59640.2) transcript is preferentially expressed in petals. Plants that lack the petal-expressed variant BPEp have larger petals as a result of increased cell size. BPEp is positively regulated downstream of APETALA3, PISTILLATA, APETALA1 and PISTILLATA3 and is negatively regulated downstream of AGAMOUS.

BLAEV0027865	TFU-TRA_sweep_pop2only, TBU-TRA_sweep_pop2only	AT1G59660	Encodes a protein with similarity to mammalian nucleoporin Nup98.Its expression is upregulated in mutants that are NUP deficient. Nucleoportin which redundantly inhibits flowering together with Nup98a through multiple pathways including clock; photoperiod, and age pathways. Gates flowering in a CONSTANS (CO)-independent mode and bypasses the CO checkpoint in photoperiodic signaling and integrated signals from multiple pathways to directly target FLOWERING LOCUS T (FT) for flowering control.
BLAEV0027856	TBU-TRA_sweep_both, TPR-TRA_sweep_pop2only, TDS-TRA_sweep_pop2only	AT1G59700	Encodes glutathione transferase belonging to the tau class of GSTs. Naming convention according to Wagner et al. (2002). The mRNA is cell-to-cell mobile.
BLAEV0027855	TPR-TRA_sweep_pop2only, TDS-TRA_sweep_pop2only	AT1G59710	actin cross-linking protein (DUF569);(source:Araport11)
BLAEV0027854	TPR-TRA_sweep_pop2only	AT1G59725	DNAJ heat shock family protein;(source:Araport11)
BLAEV0027975	TBU-TDS_sweep_pop1only, TBU-TNA_sweep_both, TFU-TBU_sweep_pop2only	AT1G61390	S-locus lectin protein kinase family protein;(source:Araport11)
BLAEV0021617	TBU-TDS_rhodxy	AT1G62305	Core-2/I-branching beta-1;6-N-acetylglucosaminyltransferase family protein;(source:Araport11)
BLAEV0023982	TBU-TDS_sweep_pop1only	AT1G69640	Encodes one of the two redundant sphingoid base hydroxylases (SBH). Involved in sphingolipid trihydroxy long-chain base (4-hydroxysphinganine) biosynthesis. Double mutants of SBHs were dwarfed and not able to progress from vegetative to reproductive growth.
BLAEV0026419	TPR-TNA_sweep_pop2only	AT1G71270	Encodes a homolog of the yeast Vps52p/SAC2. Involved in pollen tube germination and growth. Located in multiple endomembrane organelles including the golgi. The yeast protein has been shown to be located at the late Golgi and to function in a complex involved in retrograde trafficking of vesicles between the early endosomal compartment and the trans-Golgi network.
BLAEV0026418	TPR-TNA_sweep_pop2only	AT1G71310	A protein coding gene with unknown function. The 5 UTR and the first two exons and introns of this gene overlap with a RNA coding gene TER1. TER1 (GenBank accession no. HQ401284) encodes a putative template sequence (5′-CTAAACCCTA-3′) corresponding to 1.5 copies of the Arabidopsis telomere repeat (PNAS 2011; 108:73-78).
BLAEV0014853	TBU-TPR_sweep_pop1only	AT1G73700	MATE efflux family protein;(source:Araport11)
BLAEV0022185	TBU-TNA_sweep_pop1only	AT1G78080	Encodes a member of the DREB subfamily A-6 of ERF/AP2 transcription factor family (RAP2.4). The protein contains one AP2 domain. Role in mediating light and ethylene signaling. The mRNA is cell-to-cell mobile.
BLAEV0027415	TBU-TDS_sweep_pop2only	AT1G78320	Encodes glutathione transferase belonging to the tau class of GSTs. Naming convention according to Wagner et al. (2002).
BLAEV0027493	TPR-TRA_rhodxy, TPR-TRA_sweep_both, TPR-TDS_rhodxy, TPR-TDS_sweep_both, TBU-TPR_rhodxy, TBU-TPR_sweep_both	AT1G79400	member of Putative Na+/H+ antiporter family
BLAEV0022567	TBU-TPR_sweep_pop1only	AT1G79810	Dominant suppressor of det1 phenotypes. Encodes a peroxisomal protein essential for Arabidopsis growth. Inserted directly from the cytosol into peroxisomes.
BLAEV0022566	TBU-TPR_sweep_pop1only	AT1G79820	Major facilitator superfamily protein;(source:Araport11)

BLAEV0022564	TBU-TPR_sweep_pop1only	AT1G79830	This gene is predicted to encode a protein that functions as a Golgi apparatus structural component known as a golgin in mammals and yeast. A fluorescently-tagged version of GC5 co-localizes with Golgi markers; and this localization appears to be replicated using the C-terminal (139 aa) portion of the protein. The C-terminal portion of the protein can also specifically interact with two members of the Rab family of GTPases (RabH1b and RabH1c).
BLAEV0012758	TBU-TDS_sweep_pop1only	AT2G01470	Sec12p-like protein (GTP exchange protein) that functionally complements yeast sec12 null mutant. Protein is localized to the ER.
BLAEV0043922	TBU-TDS_sweep_pop1only, TBU-TNA_sweep_pop1only, TBU-TNA_sweep_both, TFU-TBU_sweep_pop2only	AT2G15620	Involved in the second step of nitrate assimilation. Its expression is induced by nitrate. The mRNA is cell-to-cell mobile.
BLAEV0043921	TBU-TDS_sweep_pop1only, TBU-TNA_sweep_pop1only, TFU-TBU_sweep_pop2only	AT2G15630	Pentatricopeptide repeat (PPR) superfamily protein;(source:Araport11)
BLAEV0043919	TBU-TDS_rhodxy, TBU-TDS_sweep_pop1only, TBU-TNA_rhodxy, TBU-TNA_sweep_both, TFU-TBU_rhodxy, TFU-TBU_sweep_both	AT2G15680	Encodes a calmodulin-like protein.
BLAEV0043918	TBU-TNA_sweep_pop1only, TFU-TBU_sweep_pop2only	AT2G15695	peptide methionine sulfoxide reductase (Protein of unknown function DUF829; transmembrane 53);(source:Araport11)
BLAEV0043982	TBU-TDS_rhodxy, TBU-TDS_sweep_both	AT2G16365	PCH1 binds and stabilizes the active (Pfr) form of phytochrome B and is involved in the formation of photobodies in the nucleus. PCH1 is expressed in evenings and is associated to the evening complex through binding to phyB; and represses hypocotyl elongation and growth. Using mass spec, the existence of the At2g16365.2 isoform has been verified, however here is no evidence that any of the other three variants are present. Atg2G16365.2 will be assigned PCH1; exon 4 and 5 in the other variants are actually another gene of the F-box/DUF295 family with gene name FDA10.
BLAEV0013733	TNA-TRA_sweep_pop1only	AT2G19730	Ribosomal L28e protein family;(source:Araport11)
BLAEV0013732	TNA-TRA_sweep_pop1only	AT2G19740	Ribosomal protein L31e family protein;(source:Araport11)
BLAEV0044174	TBU-TNA_sweep_both, TNA-TDS_sweep_pop1only	AT2G20560	DNAJ heat shock family protein;(source:Araport11)
BLAEV0044171	TFU-TDS_sweep_both	AT2G20570	Encodes GLK1; Golden2-like 1, one of a pair of partially redundant nuclear transcription factors that regulate chloroplast development in a cell-autonomous manner. GLK2, Golden2-like 2, is encoded by At5g44190. GLK1 and GLK2 regulate the expression of the photosynthetic apparatus. GLK1 is also a member of the GARP transcription factor family. Promotes anthocyanin biosynthesis via an MBW complex-dependent pathway.
BLAEV0044167	TBU-TDS_sweep_pop1only	AT2G20680	Encodes a mannanase belonging to clade 1 of the GH5 7 phylogenetic tree that exhibits high substrate affinity and catalytic efficiency on mannan substrates with main chains containing both glucose and mannose units such as konjac glucomannan and spruce galactoglucomannan. It is likely a glycoprotein.
BLAEV0044159	TFU-TDS_sweep_pop2only, TNA-TDS_sweep_pop2only	AT2G20900	diacylglycerol kinase 5;(source:Araport11)

BLAEV0020827	TBU-TPR_sweep_pop1only	AT2G21500	RING/U-box superfamily protein;(source:Araport11)
BLAEV0025520	TFU-TDS_sweep_pop2only	AT2G22720	SPT2 chromatin protein;(source:Araport11)
BLAEV0020904	TBU-TNA_sweep_pop2only, TNA-TDS_sweep_pop1only	AT2G23200	Protein kinase superfamily protein;(source:Araport11)
BLAEV0004142	TFU-TBU_sweep_pop2only	AT2G27210	Protein phosphatase which promotes stomatal ACD by establishing kinase-based signalling asymmetry in the two daughter cells.
BLAEV0004181	TNA-TRA_sweep_pop1only	AT2G27490	AT2G27490 encodes dephospho-CoA kinase. The molecular function was shown to phosphorylate the ribosyl moiety forming CoA.
BLAEV0004218	TBU-TNA_rhodxy, TBU-TNA_sweep_both, TFU-TBU_rhodxy, TFU-TBU_sweep_both, TNA-TRA_sweep_both	AT2G27740	RAB6-interacting golgin (DUF662);(source:Araport11)
BLAEV0003925	TBU-TNA_sweep_pop1only	AT2G28640	A member of EXO70 gene family; putative exocyst subunits, conserved in land plants. Arabidopsis thaliana contains 23 putative EXO70 genes, which can be classified into eight clusters on the phylogenetic tree.
BLAEV0004901	TBU-TRA_sweep_pop1only	AT2G33100	encodes a gene similar to cellulose synthase
BLAEV0004897	TBU-TRA_sweep_pop1only	AT2G33150	Encodes an organellar (peroxisome; glyoxysome) 3-ketoacyl-CoA thiolase, involved in fatty acid b-oxidation during germination and subsequent seedling growth. Mutants have defects in glyoxysomal fatty acid beta-oxidation. EC2.3.1.16 thiolase.
BLAEV0005582	TBU-TDS_sweep_both, TBU-TNA_rhodxy, TBU-TNA_sweep_both, TFU-TBU_rhodxy, TFU-TBU_sweep_both, TBU-TPR_sweep_pop1only	AT2G37540	Putative aldehyde reductase which converts aldehydes in to 1-alcohols. Part of a novel 1-alcohol biosynthesis pathway in which VLC acyl-CoAs are first reduced to aldehydes by CER3.
BLAEV0005583	TBU-TDS_sweep_both, TBU-TNA_rhodxy, TBU-TNA_sweep_both, TBU-TRA_rhodxy, TBU-TRA_sweep_both, TFU-TBU_rhodxy, TFU-TBU_sweep_both, TBU-TPR_sweep_pop1only	AT2G37550	A member of ARF GAP domain (AGD); A thaliana has 15 members, grouped into four classes.
BLAEV0002663	TFU-TBU_sweep_pop2only	AT2G38380	Peroxidase superfamily protein;(source:Araport11)
BLAEV0015380	TFU-TNA_sweep_pop1only, TBU-TNA_sweep_pop1only	AT2G43520	Encodes putative trypsin inhibitor protein which may function in defense against herbivory. Member of the defensin-like (DEFL) family.
BLAEV0002081	TBU-TDS_sweep_both	AT2G44770	Acts upstream of aperture proteins; their expression levels influence the number of aperture domains that form on the surface of developing pollen grains.
BLAEV0002080	TBU-TDS_sweep_both	AT2G44790	Encodes a uclacyanin; a protein precursor that is closely related to precursors of stellacyanins and a blue copper protein from pea pods.
BLAEV0002079	TBU-TDS_sweep_both	AT2G44800	2-oxoglutarate (2OG) and Fe(II)-dependent oxygenase superfamily protein;(source:Araport11)

BLAEV0013713	TPR-TRA_sweep_pop1only	AT2G45760	encodes a protein that is similar to BONZAI1-binding protein BAP1.
BLAEV0026597	TBU-TRA_sweep_pop1only	AT3G08930	LMBR1-like membrane protein;(source:Araport11)
BLAEV0026594	TBU-TRA_sweep_pop1only	AT3G08950	Encodes HCC1; homologue of the copper chaperone SCO1 (synthesis of cytochrome c oxidase 1) from the yeast Saccharomyces cerevisiae. SCO1 encodes a mitochondrial protein that is essential for the correct assembly of complex IV in the respiratory chain. HCC1 is localized in the mitochondrion. A chimeric yeast Sco1-Arabidopsis HCC1 protein complements yeast Sco1 activity. Embryos of hcc1 mutants became arrested at various developmental stages, mostly at the heart stage.
BLAEV0018278	TFU-TDS_sweep_pop2only	AT3G11080	receptor like protein 35;(source:Araport11)
BLAEV0007743	TBU-TNA_sweep_pop1only	AT3G14620	putative cytochrome P450 The mRNA is cell-to-cell mobile.
BLAEV0007739	TBU-TNA_sweep_pop1only, TFU-TBU_sweep_pop2only	AT3G14690	putative cytochrome P450The mRNA is cell-to-cell mobile.
BLAEV0042148	TFU-TDS_sweep_pop2only	AT3G17180	serine carboxypeptidase-like 33;(source:Araport11)
BLAEV0042138	TFU-TDS_sweep_pop2only	AT3G17210	Encodes a heat stable protein with antimicrobial and antifungal activity.
BLAEV0007344	TBU-TPR_sweep_pop1only	AT3G17770	Dihydroxyacetone kinase;(source:Araport11)
BLAEV0007340	TBU-TPR_sweep_pop1only	AT3G17790	Expression is upregulated in the shoot of cax1/cax3 mutant and is responsive to phosphate (Pi) and not phosphite (Phi) in roots and shoots.
BLAEV0042190	TPR-TNA_sweep_pop2only, TBU-TPR_sweep_pop1only	AT3G18000	Encodes a N-methyltransferase-like protein. Double mutants of NMT1 and NMT3 are defective in leaf; root, flower, seed, and pollen development.
BLAEV0007220	TBU-TDS_sweep_pop1only	AT3G18524	Encodes a DNA mismatch repair homolog of human MutS gene; MSH6. MSH2 is involved in maintaining genome stability and repressing recombination of mismatched heteroduplexes.There are four MutS genes in Arabidopsis, MSH2, MSH3, MSH6, and MSH7, which all act as heterodimers and bind to 51-mer duplexes. MSH2 has different binding specificity to different mismatches in combination with MSH3, MSH6, or MSH7.
BLAEV0041998	TFU-TDS_sweep_pop2only, TBU-TDS_sweep_pop2only	AT3G18680	Encodes a functional UMP Kinase located in the plastid that binds to group II intron plastid transcription products. Mutants show decreased accumulation of target transcripts/proteins.
BLAEV0041997	TBU-TDS_sweep_pop2only	AT3G18690	Encodes a nuclear-localized member of a plant specific gene family involved in mediating responses to pathogens. Interacts with WRKY transcriptional regulators.
BLAEV0006925	TFU-TNA_sweep_pop1only	AT3G20410	calmodulin-domain protein kinase CDPK isoform 9 (CPK9)
BLAEV0006924	TFU-TNA_sweep_pop1only	AT3G20420	double-stranded RNA binding / ribonuclease III. Required for 3' external transcribed spacer (ETS) cleavage of the pre-rRNA in vivo. Localizes in the nucleus and cytoplasm.
BLAEV0006923	TFU-TNA_sweep_pop1only	AT3G20430	phosphorylated adapter RNA export-like protein;(source:Araport11)
BLAEV0006922	TFU-TNA_sweep_pop1only	AT3G20440	Encodes BE1; a putative glycoside hydrolase. Involved in organogenesis and somatic embryogenesis by regulating carbohydrate metabolism. Mutation in BE1 has pleotrophic effect on the whole plant development.
BLAEV0031634	TFU-TBU_sweep_pop2only, TBU-TPR_sweep_pop1only	AT3G23610	Encodes a dual specificity protein phosphatase whose activity is modulated by CaM binding.
BLAEV0006403	TPR-TDS_sweep_pop2only	AT3G24150	hypothetical protein;(source:Araport11)
BLAEV0031514	TFU-TDS_sweep_pop1only	AT3G25050	Encodes an endotransglucosylase that cleaves the beta-1;4-glucosidic linkage in amorphous cellulose and ligates the nascent reducing end to a non-reducing terminus of either cellulosic or xyloglucan oligosaccharide. Higher expression in flowers and in response to IAA treatment.

BLAEV0031501	TFU-TRA_sweep_pop1only, TFU-TBU_sweep_pop1only	AT3G25170	Member of a diversely expressed predicted peptide family showing sequence similarity to tobacco Rapid Alkalinization Factor (RALF); and is believed to play an essential role in the physiology of Arabidopsis. Consists of a single exon and is characterized by a conserved C-terminal motif and N-terminal signal peptide.
BLAEV0031492	TFU-TRA_sweep_pop1only	AT3G25290	Cytochrome b561 and DOMON domain (CYBDOM) protein involved in iron homeostasis.
BLAEV0024962	TBU-TRA_sweep_pop1only	AT3G25860	Encodes the dihydrolipoamide S-acetyltransferase subunit of the plastid Pyruvate Dehydrogenase Complex (E2). Mutant has embryo defect.
BLAEV0041550	TNA-TRA_sweep_pop1only	AT3G44620	protein-tyrosine phosphatase;(source:Araport11)
BLAEV0039994	TFU-TNA_sweep_pop2only, TFU-TDS_sweep_pop2only	AT3G45190	SIT4 phosphatase-associated family protein;(source:Araport11)
BLAEV0030024	TBU-TDS_sweep_both, TBU-TNA_sweep_both, TFU-TBU_sweep_both	AT3G46730	NB-ARC domain-containing disease resistance protein;(source:Araport11)
BLAEV0051949	TPR-TRA_rhodxy, TPR-TRA_sweep_both, TPR-TNA_rhodxy, TPR-TNA_sweep_both	AT3G47170	Encodes enzymes that can efficiently convert putrescine and caffeoyl-CoA to di-caffeoyl putrescine. Has a preference for caffeoyl CoA and putrescine.
BLAEV0051998	TBU-TDS_rhodxy, TBU-TDS_sweep_both, TBU-TNA_rhodxy, TBU-TNA_sweep_pop2only, TPR-TNA_sweep_pop2only	AT3G47710	BNQ3 belongs to a family of atypical non-DNA binding basic helix-loop-helix (bHLH) proteins that heterodimerize with and negatively regulate bHLH transcription factors. Directly and negatively regulated by AP3 and PI in petals. Required for appropriate regulation of flowering time.May also have a role in regulating light responses.
BLAEV0052128	TPR-TNA_sweep_pop2only	AT3G48750	A-type cyclin-dependent kinase. Together with its specific inhibitor; the Kip-related protein, KRP2 they regulate the mitosis-to-endocycle transition during leaf development. Dominant negative mutations abolish cell division. Loss of function phenotype has reduced fertility with failure to transmit via pollen. Pollen development is arrested at the second mitotic division. Expression is regulated by environmental and chemical signals. Part of the promoter is responsible for expression in trichomes. Functions as a positive regulator of cell proliferation during development of the male gametophyte, embryo and endosperm. Phosphorylation of threonine 161 is required for activation of its associated kinase.
BLAEV0051787	TFU-TNA_sweep_pop2only	AT3G49160	Expression of the gene is downregulated in the presence of paraquat; an inducer of photoxidative stress.
BLAEV0051783	TFU-TNA_sweep_pop2only, TFU-TBU_sweep_pop2only	AT3G49170	Tetratricopeptide repeat (TPR)-like superfamily protein;(source:Araport11)
BLAEV0051784	TFU-TNA_sweep_pop2only, TFU-TBU_sweep_pop2only	AT3G49180	Transducin/WD40 repeat-like superfamily protein;(source:Araport11)
BLAEV0051725	TBU-TDS_sweep_pop2only	AT3G49680	Encodes a chloroplast branched-chain amino acid aminotransferase. Complements the yeast leu/iso-leu/val auxotrophy mutant.
BLAEV0051595	TFU-TBU_sweep_pop1only	AT3G50740	UGT72E1 is an UDPG:coniferyl alcohol glucosyltransferase which specifically glucosylates sinapyl- and coniferyl aldehydes. The enzyme is thought to be involved in lignin metabolism.
BLAEV0032491	TBU-TNA_sweep_pop1only	AT3G55980	CCCH-type zinc finger protein involved in salt stress and immune responses.

BLAEV0032931	TBU-TDS_sweep_pop1only, TNA-TDS_sweep_pop1only	AT3G61050	Encodes a novel transcriptional regulator; a calcium-dependent lipid-binding protein containing a C2 domain, that binds specifically to the promoter of THAS1 (thalianol synthase 1). It can bind ceramide and is involved in drought and salt tolerance.
BLAEV0015322	TFU-TDS_sweep_pop1only, TPR-TDS_sweep_pop1only, TDS-TRA_sweep_pop2only	AT4G00030	Putative fibrillin; located in chloroplast, involved in photosynthesis.
BLAEV0015211	TBU-TDS_sweep_pop1only	AT4G00330	high overall homology to CRCK1
BLAEV0015207	TBU-TDS_sweep_pop1only	AT4G00370	Encodes an inorganic phosphate transporter (PHT4;4) that can transport ascorbate and is located in the chloroplast envelope membrane. It has been shown to play a role in the xanthophyll cycle during photosynthesis and may be required for tolerance to strong light stress.
BLAEV0014722	TBU-TDS_sweep_pop1only	AT4G04180	P-loop containing nucleoside triphosphate hydrolases superfamily protein;(source:Araport11)
BLAEV0000639	TPR-TRA_sweep_pop2only	AT4G05420	Structurally similar to damaged DNA binding proteins. DDB1a is part of a 350 KDa nuclear localized DET1 protein complex. This complex may physically interact with histone tails and while bound to chromatin- repress transcription of genes involved in photomorphogenesis. DDB1a is shown to be RUB-modified.
BLAEV0015325	TPR-TDS_sweep_pop1only, TDS-TRA_sweep_pop2only	AT4G08500	Encodes a member of the A1 subgroup of the MEKK (MAPK/ERK kinase kinase) family. MEKK is another name for Mitogen-Activated Protein Kinase Kinase Kinase (MAPKKK or MAP3K). This subgroup has four members: At4g08500 (MEKK1; also known as ARAKIN, MAP3Kb1, MAPKKK8), At4g08480 (MEKK2, also known as MAP3Kb4, MAPKKK9), At4g08470 (MEKK3, also known as MAP3Kb3, MAPKKK10) and At4g12020 (MEKK4, also known as MAP3Kb5, MAPKKK11, WRKY19). Nomenclatures for mitogen-activated protein kinases are described in Trends in Plant Science 2002, 7(7):301. Mediates cold, salt, cadmium and wounding stress signalling. Phosphorylates MEK1.
BLAEV0001106	TFU-TNA_sweep_pop2only, TBU-TDS_sweep_pop1only, TFU-TBU_sweep_pop2only	AT4G08540	One of a pair of paralogs (the other is AT1G77890)that is a subunit of the lass III phosphatidylinositol 3-kinase (PI3K) complex;but is not essential for PI3P biosynthesis.
BLAEV0001104	TFU-TNA_sweep_pop2only, TBU-TDS_sweep_pop1only	AT4G08550	electron carrier/ protein disulfide oxidoreductase;(source:Araport11)
BLAEV0001102	TFU-TNA_sweep_pop2only, TBU-TDS_sweep_pop1only, TFU-TBU_sweep_pop2only	AT4G08570	Heavy metal transport/detoxification superfamily protein;(source:Araport11)
BLAEV0001103	TBU-TDS_sweep_pop1only, TFU-TBU_sweep_pop2only	AT4G08570	Heavy metal transport/detoxification superfamily protein;(source:Araport11)
BLAEV0001101	TFU-TNA_sweep_pop2only, TBU-TDS_sweep_pop1only, TFU-TBU_sweep_pop2only	AT4G08620	Encodes a high-afnity sulfate transporter. Contains STAS domain. Expressed in roots and guard cells. Up-regulated by sulfur deficiency.
BLAEV0001100	TFU-TNA_sweep_pop2only, TBU-TDS_sweep_pop1only, TFU-TBU_sweep_pop2only	AT4G08670	Bifunctional inhibitor/lipid-transfer protein/seed storage 2S albumin superfamily protein;(source:Araport11)
BLAEV0046613	TBU-TRA_sweep_pop2only, TDS-TRA_sweep_pop2only	AT4G08690	Sec14p-like phosphatidylinositol transfer family protein;(source:Araport11)
BLAEV0015374	TFU-TNA_sweep_both	AT4G09830	nuclear receptor family 2 group C protein;(source:Araport11)

BLAEV0015422	TFU-TNA_sweep_pop1only, TFU-TRA_sweep_pop1only	AT4G10150	RING/U-box superfamily protein;(source:Araport11)
BLAEV0015516	TPR-TRA_sweep_pop2only, TBU-TPR_sweep_pop1only	AT4G10850	Nodulin MtN3 family protein;(source:Araport11)
BLAEV0015050	TFU-TNA_sweep_pop2only	AT4G11120	translation elongation factor Ts (EF-Ts);(source:Araport11)
BLAEV0014968	TPR-TNA_sweep_pop2only	AT4G11860	FAM63A-like protein (DUF544);(source:Araport11)
BLAEV0043884	TBU-TDS_rhodxy, TBU-TDS_sweep_both, TBU-TNA_rhodxy, TBU-TNA_sweep_both	AT4G13070	RNA-binding CRS1 / YhbY (CRM) domain protein;(source:Araport11)
BLAEV0031269	TPR-TDS_sweep_pop1only	AT4G13850	Encodes a glycine-rich RNA-binding protein. Gene expression is induced by cold.
BLAEV0031265	TPR-TNA_sweep_pop2only, TPR-TDS_sweep_both, TBU-TPR_sweep_pop1only, TNA-TRA_sweep_pop1only	AT4G13940	Encodes a S-adenosyl-L-homocysteine hydrolase required for DNA methylation-dependent gene silencing. The mRNA is cell-to-cell mobile.
BLAEV0031234	TPR-TDS_sweep_pop1only	AT4G14210	Encodes phytoene desaturase (phytoene dehydrogenase); an enzyme that catalyzes the desaturation of phytoene to zeta-carotene during carotenoid biosynthesis. Processed protein is localized to the plastid.
BLAEV0038577	TBU-TPR_sweep_pop1only	AT4G14220	encodes a RING-type E3 ubiquitin ligase implicated in gametogenesis. RHF1a can interact with the cell cycle inhibitor ICK4/KRP6 in vitro. It apppears to target ICK4KRP6 for degradation following meiosis in order to allow the mitoses associated with megagametogenesis and microgametogenesis to occur. RHF1a is expressed in the carpels throughout floral development. It is expressed in various tissues of the anthers during the early stages of anther development but not in stage 12 flowers and beyond. The mRNA is cell-to-cell mobile.
BLAEV0031198	TNA-TDS_sweep_pop1only	AT4G14410	Basic helix loop helix transcription factor involved in stress mediated signaling during germination.
BLAEV0031200	TNA-TDS_sweep_pop1only	AT4G14420	HR-like lesion-inducing protein-like protein;(source:Araport11)
BLAEV0031201	TNA-TDS_sweep_pop1only	AT4G14430	Encodes a peroxisomal delta3; delta2-enoyl CoA isomerase, involved in unsaturated fatty acid degradation. This enzyme might also be involved in the conversion of indole-3-butyric acid to indole-3-acetic acid via a beta-oxidation-like pathway.
BLAEV0047188	TPR-TRA_sweep_pop1only	AT4G17880	MYC4 is bHLH transcriptional regulator. It functions as a JAZ-interacting transcription factor that acts together with MYC2 and MYC3 to activate JA-responses. It also functions in blue light mediated secondary cell wall biogenesis via regulation of NST1 expression. MYC4 directly binds to NST1 promoter and activates its expression.
BLAEV0046811	TFU-TDS_sweep_pop2only	AT4G18670	Leucine rich extensin protein involved in cell wall biogenesis and organization. Interacts with several members of the RALF family of ligand peptides.
BLAEV0036736	TFU-TDS_sweep_pop2only	AT4G19830	member of the thylakoid lumen proteome
BLAEV0009076	TPR-TDS_sweep_pop2only	AT4G20720	dentin sialophosphoprotein-like protein;(source:Araport11)
BLAEV0049765	TFU-TDS_rhodxy, TFU-TDS_sweep_both	AT4G23290	Encodes a cysteine-rich receptor-like protein kinase.
BLAEV0049499	TFU-TDS_sweep_pop2only	AT4G25540	encodes a DNA mismatch repair homolog of human MutS gene; MSH6. There are four MutS genes in Arabidopsis, MSH2, MSH3, MSH6, and MSH7, which all act as heterodimers and bind to 51-mer

			duplexes. MSH2*MSH3 heterodimers bound 'insertion-deletion' DNA with three nucleotides (+AAG) or one nucleotide (+T) looped out much better than they bound DNA with a base/base mispair (T/G).
BLAEV0049498	TFU-TDS_sweep_pop2only	AT4G25550	Cleavage/polyadenylation specificity factor; 25kDa subunit;(source:Araport11)
BLAEV0049497	TFU-TDS_sweep_pop2only	AT4G25560	LAF1 is a R2R3-MYB transcription factor and positive regulator of the phyA photoresponse. Interaction of LAF1 with HFR1 stabilize the proteins against ubiquitination by COP1(AT2G32950) and subsequent degrations. Mutants have an elongated hypocotyl specifically under far-red light but retain wild-type responses to other light wavelengths.
BLAEV0049496	TFU-TDS_sweep_pop2only	AT4G25570	Encodes cytochrome b561.
BLAEV0049495	TFU-TDS_sweep_pop2only	AT4G25580	CAP160 protein;(source:Araport11)
BLAEV0015781	TFU-TNA_sweep_pop2only	AT4G25630	encodes a fibrillarin; a key nucleolar protein in eukaryotes which associates with box C/D small nucleolar RNAs (snoRNAs) directing 2'-O-ribose methylation of the rRNA. This gene also encodes a novel box C/D snoRNA, U60.2f in its fifth intron that accumulates in seedlings and that their targeted residue on the 25 S rRNA is methylated. The mRNA is cell-to-cell mobile.
BLAEV0046151	TFU-TNA_sweep_pop1only	AT4G25720	This locus encodes a protein with similarity to gamma-glutamylcyclotransferase that may be involved in catalyzing the formation of pyroglutate residue on proteins that have been post-translationally processed to reveal a glutamine at their N-terminus. Enzymatic assays to test the function of this protein were performed using a truncated form of the protein lacking a signal peptide that is most similar to the AT4G25720.1 protein model.
BLAEV0049300	TFU-TDS_sweep_pop2only	AT4G27270	Quinone reductase family protein;(source:Araport11)
BLAEV0045883	TBU-TPR_sweep_pop1only	AT4G28703	RmlC-like cupins superfamily protein;(source:Araport11)
BLAEV0045881	TBU-TPR_sweep_pop1only	AT4G28706	pfkB-like carbohydrate kinase family protein;(source:Araport11)
BLAEV0014689	TBU-TPR_sweep_pop1only	AT4G29770	Target of trans acting-siR480/255. Testing.
BLAEV0048969	TBU-TDS_sweep_pop1only	AT4G29890	choline monooxygenase; putative (CMO-like);(source:Araport11)
BLAEV0048968	TBU-TDS_sweep_pop1only	AT4G29920	Encodes a member of an eight-gene family (SMAX1 and SMAX1-like) that has weak similarity to AtHSP101; a ClpB chaperonin required for thermotolerance. Loss of function mutants show increased sensitivity to salt stress and drought. RNA G-quadruplex formation suppresses SMXL4/5 translation and restricts phloem differentiation.
BLAEV0048262	TBU-TNA_sweep_pop1only	AT4G35070	SBP (S-ribonuclease binding protein) family protein;(source:Araport11)
BLAEV0048261	TBU-TNA_sweep_pop1only	AT4G35080	Encodes a nuclear-encoded chloroplast protein that plays an important role in vegetative growth; female gametogenesis, and embryogenesis likely by mediating chloroplast integrity and development.
BLAEV0048260	TBU-TNA_sweep_pop1only	AT4G35090	Encodes a peroxisomal catalase; highly expressed in bolts and leaves. mRNA expression patterns show circadian regulation with mRNA levels being high in the subjective early morning. Loss of function mutations have increased H2O2 levels and increased H2O2 sensitivity. Mutants accumulate more toxic ions yet show decreased sensitivity to Li+. This decreased sensitivity is most likely due to an insensitivity to ethylene. Note that in Queval et al. (2007) Plant Journal, 52(4):640, SALK_057998 is named as cat2-1, SALK_076998 is named as cat2-2; in Bueso et al. (2007) Plant Journal, 52(6):1052, SALK_076998 is named as cat2-1. TAIR has adopted the nomenclature consistent with that in Bueso et al. (2007) after consultation with the authors: SALK_076998 (cat2-1), SALK_057998 (cat2-2).
BLAEV0048259	TBU-TNA_sweep_pop1only	AT4G35100	A member of the plasma membrane intrinsic protein PIP. functions as aquaporin. Salt-stress-inducible MIP

BLAEV0048252	TBU-TNA_sweep_pop1only	AT4G35190	Putative lysine decarboxylase family protein;(source:Araport11)
BLAEV0045172	TBU-TPR_sweep_pop1only	AT4G35733	F-box SKIP23-like protein (DUF295);(source:Araport11)
BLAEV0045168	TBU-TPR_sweep_pop1only	AT4G35800	Encodes the unique largest subunit of nuclear DNA-dependent RNA polymerase II; the ortholog of budding yeast RPB1 and a homolog of the E. coli RNA polymerase beta prime subunit.
BLAEV0045167	TBU-TPR_sweep_pop1only	AT4G35830	Encodes an aconitase that can catalyze the conversion of citrate to isocitrate through a cis-aconitate intermediate; indicating that it may participate in the TCA cycle and other primary metabolic pathways. The protein is believed to accumulate in the mitochondria and the cytosol. It affects CSD2 (At2g28190 - a superoxide dismutase) transcript levels and may play a role in the response to oxidative stress. This enzyme can also specifically bind to the 5' UTR of CSD2 in vitro.
BLAEV0047841	TBU-TDS_sweep_both	AT4G39490	member of CYP96A
BLAEV0020592	TBU-TRA_sweep_pop1only, TPR-TRA_sweep_pop1only	AT5G10520	ROP binding protein kinases 1;(source:Araport11)
BLAEV0020589	TBU-TRA_sweep_pop1only	AT5G10560	Glycosyl hydrolase family protein;(source:Araport11)
BLAEV0020360	TPR-TRA_sweep_pop1only	AT5G13480	Encodes a protein with similarity to yeast Pfs2p; an mRNA processing factor. Involved in regulation of flowering time; affects FCA mRNA processing. Homozygous mutants are late flowering, null alleles are embryo lethal.
BLAEV0020359	TPR-TRA_sweep_pop1only	AT5G13500	Hyp O-arabinosyltransferase-like protein;(source:Araport11)
BLAEV0020358	TPR-TRA_sweep_pop1only	AT5G13510	Ribosomal protein L10 family protein;(source:Araport11)
BLAEV0020356	TPR-TRA_sweep_pop1only	AT5G13520	peptidase M1 family protein;(source:Araport11)
BLAEV0020355	TPR-TRA_sweep_pop1only	AT5G13530	Encodes KEEP ON GOING (KEG); a RING E3 ligase involved in abscisic acid signaling. KEG is essential for Arabidopsis growth and development. ABA promotes KEG degradation via the ubiquitin dependent 26S proteasome pathway. Associates with and ubiquitinates MKK4 and MKK5 to regulate plant immunity.
BLAEV0020354	TPR-TRA_sweep_pop1only	AT5G13550	Encodes a sulfate transporter.
BLAEV0020353	TPR-TRA_sweep_pop1only	AT5G13560	structural maintenance of chromosomes protein;(source:Araport11)
BLAEV0017250	TFU-TDS_sweep_pop1only, TPR-TRA_sweep_pop1only	AT5G20380	Encodes an inorganic phosphate transporter (PHT4;5).
BLAEV0010926	TBU-TDS_sweep_pop1only, TBU-TRA_rhodxy, TBU-TRA_sweep_both, TNA-TRA_sweep_pop1only	AT5G20940	Glycosyl hydrolase family protein;(source:Araport11)
BLAEV0030010	TBU-TDS_rhodxy, TBU-TDS_sweep_both, TBU-TNA_sweep_both, TBU-TRA_sweep_pop1only, TFU-TBU_rhodxy, TFU-TBU_sweep_both	AT5G25560	CHY-type/CTCHY-type/RING-type Zinc finger protein;(source:Araport11)
BLAEV0030007	TBU-TNA_sweep_both, TFU-TBU_rhodxy, TFU-TBU_sweep_both	AT5G25570	polyamine-modulated factor 1-binding protein;(source:Araport11)
BLAEV0030012	TBU-TDS_rhodxy, TBU-TDS_sweep_both, TBU-	AT5G25580	hypothetical protein;(source:Araport11)

	TNA_sweep_both, TBU-TRA_sweep_pop1only, TFU-TBU_rhodxy, TFU-TBU_sweep_both		
BLAEV0030013	TBU-TDS_rhodxy, TBU-TDS_sweep_both, TBU-TNA_sweep_both, TFU-TBU_rhodxy, TFU-TBU_sweep_both	AT5G25590	DNA ligase (DUF630 and DUF632);(source:Araport11)
BLAEV0030016	TBU-TDS_rhodxy, TBU-TDS_sweep_both, TBU-TNA_sweep_both, TBU-TRA_sweep_pop1only, TFU-TBU_rhodxy, TFU-TBU_sweep_both	AT5G25610	responsive to dehydration 22 (RD22) mediated by ABA
BLAEV0030020	TBU-TDS_sweep_both, TBU-TNA_sweep_both, TFU-TBU_sweep_both	AT5G25630	Tetratricopeptide repeat (TPR)-like superfamily protein;(source:Araport11)
BLAEV0030021	TBU-TDS_sweep_both, TBU-TNA_sweep_both, TBU-TRA_sweep_pop1only, TFU-TBU_sweep_both	AT5G25752	Chloroplast-localized rhomboid-like protein.
BLAEV0030022	TBU-TDS_sweep_both, TBU-TNA_sweep_both, TBU-TRA_sweep_pop1only, TFU-TBU_sweep_both	AT5G25754	RNA polymerase I-associated factor PAF67;(source:Araport11)
BLAEV0030034	TBU-TDS_rhodxy, TBU-TDS_sweep_both, TBU-TNA_rhodxy, TBU-TNA_sweep_both, TFU-TBU_rhodxy, TFU-TBU_sweep_both	AT5G25760	mutant displays sucrose-dependent seedling development and reduced lateral root production. PEX4 interacts with PEX22 in a yeast two-hybrid. Necessary for peroxisome biogenesis. The PEX4 and PEX22 pair may be important during the remodeling of peroxisome matrix contents as glyoxysomes transition to leaf peroxisomes.
BLAEV0030033	TBU-TDS_rhodxy, TBU-TDS_sweep_both, TBU-TNA_rhodxy, TBU-TNA_sweep_both, TFU-TBU_rhodxy, TFU-TBU_sweep_both	AT5G25770	alpha/beta-Hydrolases superfamily protein;(source:Araport11)
BLAEV0030032	TBU-TDS_rhodxy, TBU-TDS_sweep_both, TBU-TNA_rhodxy, TBU-TNA_sweep_both, TFU-	AT5G25800	Polynucleotidyl transferase; ribonuclease H-like superfamily protein;(source:Araport11)

	TBU_rhodxy, TFU-TBU_sweep_both		
BLAEV0030043	TBU-TDS_sweep_both, TBU-TNA_sweep_both, TFU-TBU_sweep_pop2only, TBU-TPR_sweep_pop1only	AT5G25820	Exostosin family protein;(source:Araport11)
BLAEV0030046	TBU-TDS_sweep_both, TBU-TNA_sweep_both, TFU-TBU_sweep_pop2only, TBU-TPR_sweep_pop1only	AT5G25830	Encodes a member of the GATA factor family of zinc finger transcription factors.
BLAEV0035880	TPR-TRA_sweep_pop1only	AT5G27110	Tetratricopeptide repeat (TPR)-like superfamily protein;(source:Araport11)
BLAEV0030226	TBU-TDS_rhodxy, TBU-TDS_sweep_both, TBU-TNA_rhodxy, TBU-TNA_sweep_pop2only, TFU-TBU_rhodxy	AT5G27760	Hypoxia-responsive family protein;(source:Araport11)
BLAEV0035878	TBU-TRA_sweep_pop1only, TPR-TRA_sweep_pop1only	AT5G27970	ARM repeat superfamily protein;(source:Araport11)
BLAEV0043784	TBU-TDS_sweep_both, TBU-TNA_sweep_pop2only	AT5G36230	ARM repeat superfamily protein;(source:Araport11)
BLAEV0043771	TBU-TDS_sweep_pop1only, TPR-TDS_sweep_pop1only, TNA-TDS_sweep_pop1only	AT5G36940	Encodes a member of the cationic amino acid transporter (CAT) subfamily of amino acid polyamine choline transporters. Does not mediate efficient uptake of basic amino acids in yeast or Xenopus systems but can transport neutral and acidic amino acid analogs.
BLAEV0043746	TPR-TDS_sweep_pop2only	AT5G37310	Endomembrane protein 70 protein family;(source:Araport11)
BLAEV0043623	TFU-TDS_sweep_pop2only	AT5G38780	SABATH methyltransferase.
BLAEV0038278	TPR-TDS_sweep_pop2only, TDS-TRA_sweep_both	AT5G39940	FAD/NAD(P)-binding oxidoreductase family protein;(source:Araport11)
BLAEV0009897	TFU-TNA_sweep_pop2only	AT5G42232	Encodes a defensin-like (DEFL) family protein.
BLAEV0037582	TBU-TDS_sweep_both, TBU-TNA_sweep_both, TFU-TBU_sweep_both, TBU-TPR_sweep_pop1only	AT5G43320	Member of CKL gene family (CKL-C group)
BLAEV0037583	TBU-TDS_sweep_both, TBU-TNA_sweep_both, TFU-TBU_sweep_both, TBU-TPR_sweep_pop1only	AT5G43330	predicted to encode a cytosolic malate dehydrogenase. The mRNA is cell-to-cell mobile.
BLAEV0037589	TBU-TDS_sweep_pop1only	AT5G43350	Encodes an inorganic phosphate transporter Pht1;1. Mutants display enhanced arsenic accumulation. Under high arsenate concentrations; PHT1;1 levels are reduced and it is delocalized from the plasma membrane. Members of the Pht1 family of phosphate transporters include: Pht1;1/At5g43350, Pht1;2/At5g43370, Pht1;3/At5g43360, Pht1;4/At2g38940, Pht1;5/At2g32830, Pht1;6/At5g43340, Pht1;7/At3g54700, Pht1;8/At1g20860, Pht1;9/At1g76430 (Plant Journal 2002, 31:341).PHT1;1 expression is transcriptionally regulated by WRKY6 and by PHR1.

BLAEV0037592	TFU-TDS_rhodxy, TFU-TDS_sweep_pop1only, TBU-TDS_sweep_pop1only, TBU-TNA_sweep_pop1only	AT5G43350	Encodes an inorganic phosphate transporter Pht1;1. Mutants display enhanced arsenic accumulation. Under high arsenate concentrations; PHT1;1 levels are reduced and it is delocalized from the plasma membrane. Members of the Pht1 family of phosphate transporters include: Pht1;1/At5g43350, Pht1;2/At5g43370, Pht1;3/At5g43360, Pht1;4/At2g38940, Pht1;5/At2g32830, Pht1;6/At5g43340, Pht1;7/At3g54700, Pht1;8/At1g20860, Pht1;9/At1g76430 (Plant Journal 2002, 31:341).PHT1;1 expression is transcriptionally regulated by WRKY6 and by PHR1.
BLAEV0037588	TBU-TDS_rhodxy, TBU-TDS_sweep_both, TBU-TNA_rhodxy, TBU-TNA_sweep_both, TPR-TRA_sweep_both, TBU-TPR_sweep_both, TNA-TRA_rhodxy, TNA-TRA_sweep_both, TDS-TRA_rhodxy, TDS-TRA_sweep_both	AT5G43360	Encodes Pht1;3; a member of the Pht1 family of phosphate transporters which include: Pht1;1/At5g43350, Pht1;2/At5g43370, Pht1;3/At5g43360, Pht1;4/At2g38940, Pht1;5/At2g32830, Pht1;6/At5g43340, Pht1;7/At3g54700, Pht1;8/At1g20860, Pht1;9/At1g76430 (Plant Journal 2002, 31:341).
BLAEV0037593	TFU-TDS_rhodxy, TFU-TDS_sweep_pop1only, TBU-TDS_sweep_pop1only, TBU-TNA_sweep_pop1only	AT5G43380	encodes a type I serine/threonine protein phosphatase expressed in expressed in roots; rosettes and flowers.
BLAEV0037633	TBU-TDS_sweep_pop1only	AT5G43830	aluminum induced protein with YGL and LRDR motifs;(source:Araport11)
BLAEV0042555	TPR-TRA_sweep_pop1only	AT5G44200	Encodes a nuclear cap-binding protein that forms a heterodimeric complex with ABH1 (ATCBP80) and is likely to participate in RNA metabolism. Its mRNA is ubiquitously expressed.Loss of function mutations suggest a role in processing of pri-miRNA and mRNA splicing.
BLAEV0042553	TPR-TRA_sweep_pop1only	AT5G44230	Pentatricopeptide repeat (PPR) superfamily protein;(source:Araport11)
BLAEV0037921	TBU-TPR_sweep_pop1only	AT5G45980	Arabidopsis thaliana WOX8 protein. Contains similarity to homeodomain transcription factor. Positively regulates early embryonic growth. Together with CLE8 it forms a signaling module that promotes seed growth and overall seed size.
BLAEV0043327	TBU-TPR_sweep_pop2only	AT5G46220	Encodes a protein with alkaline ceramidase activity that is involved in regulation of turgor during pollen tube growth and silique stomatal movement. Tod1 is expressed specifically in pollen and silique guard cells. Loss of function mutations have reduced fertility due to defects in pollen transmission.
BLAEV0037966	TNA-TDS_sweep_pop1only	AT5G46350	member of WRKY Transcription Factor; Group II-c Increased triacylglycerols (TAGs) after being transiently expressed in Nicotiana benthamiana. Activated by LEC2.
BLAEV0043305	TBU-TDS_sweep_pop2only, TPR-TRA_sweep_both, TPR-TNA_sweep_both, TPR-TDS_sweep_both, TBU-TPR_sweep_pop2only, TNA-TDS_sweep_pop2only, TDS-TRA_sweep_pop1only	AT5G46510	Disease resistance protein (TIR-NBS-LRR class) family;(source:Araport11)
BLAEV0038021	TPR-TRA_sweep_pop1only	AT5G46740	Encodes a ubiquitin-specific protease.

BLAEV0038022	TPR-TRA_sweep_pop1only	AT5G46750	A member of ARF GAP domain (AGD); A thaliana has 15 members, grouped into four classes. The mRNA is cell-to-cell mobile.
BLAEV0038024	TPR-TRA_sweep_pop1only	AT5G46760	MYC3 is a JAZ-interacting transcription factor that act together with MYC2 and MYC4 to activate JA-responses. The mRNA is cell-to-cell mobile.
BLAEV0043491	TBU-TNA_rhodxy, TBU-TNA_sweep_both, TBU-TPR_sweep_pop1only, TNA-TDS_sweep_pop1only	AT5G47720	Encodes a functional acetoacetyl-CoA thiolase that is functionally redundant with AACT2. Loss-of-function mutants show no apparent growth phenotypes.
BLAEV0043497	TBU-TNA_rhodxy, TBU-TNA_sweep_both, TNA-TDS_sweep_pop1only	AT5G47740	Adenine nucleotide alpha hydrolases-like superfamily protein;(source:Araport11)
BLAEV0013526	TBU-TDS_rhodxy, TBU-TDS_sweep_both, TNA-TDS_sweep_both, TDS-TRA_sweep_pop2only	AT5G48000	Encodes a member of the CYP708A family of cytochrome P450 enzymes. THAH appears to add a hydroxyl group to the triterpene thalianol. thah1 mutants have an elevated accumulation of thalianol. thah1-1 mutants have longer roots than wild type plants. Thalian-diol and desaturated thalian-diol are lost from the root extracts of thah1-1 mutants. Overexpression of the sequence from At5g48000.1 rescues the thah1-1 mutant phenotype (Field 2008); it is unknown whether the shorter sequences associated with other gene models would provide functional complementation.
BLAEV0045217	TFU-TDS_rhodxy, TFU-TDS_sweep_pop2only	AT5G48600	member of SMC subfamily
BLAEV0035794	TBU-TNA_sweep_pop2only	AT5G49480	AtCP1 encodes a novel Ca2+-binding protein; which shares sequence similarities with calmodulins. The expression of AtCP1 is induced by NaCl. The mRNA is cell-to-cell mobile.
BLAEV0015534	TBU-TPR_sweep_pop1only	AT5G50390	Pentatricopeptide repeat (PPR-like) superfamily protein;(source:Araport11)
BLAEV0015557	TFU-TRA_sweep_both, TPR-TNA_sweep_pop2only, TNA-TRA_sweep_both, TDS-TRA_sweep_pop2only	AT5G50930	Encodes a protein with similarity to mammalian MHF1 that acts in the same pathway as FANCM to restrain class II meiotic crossing over; and acts with FANCM during meiosis and to repair cross-links. It also assumes an opposing role from FANCM in homologous recombination and only FANCM is essential for replicative repair in the absence of the endonuclease MUS81.
BLAEV0015559	TNA-TRA_sweep_pop1only	AT5G50970	transducin family protein / WD-40 repeat family protein;(source:Araport11)
BLAEV0015705	TFU-TNA_rhodxy, TFU-TNA_sweep_both, TFU-TBU_rhodxy, TFU-TBU_sweep_pop2only	AT5G51720	Encodes a protein with biochemical; structural, and biophysical characteristics of a NEET protein. It plays a key role in plant development, senescence, reactive oxygen homeostasis, and Fe metabolism.
BLAEV0015709	TFU-TNA_sweep_pop2only	AT5G51750	subtilase 1.3;(source:Araport11)
BLAEV0015711	TPR-TNA_sweep_pop2only	AT5G51760	Encodes AHG1 (ABA-hypersensitive germination 1); a putative protein phosphatase 2C (PP2C). Expressed in seeds. AHG1 functions in seed development and germination.
BLAEV0015713	TFU-TNA_sweep_both, TPR-TNA_sweep_both, TNA-TDS_rhodxy, TNA-TDS_sweep_both	AT5G51780	basic helix-loop-helix (bHLH) DNA-binding superfamily protein;(source:Araport11)
BLAEV0015717	TFU-TNA_sweep_pop2only	AT5G51810	Encodes gibberellin 20-oxidase. Involved in gibberellin biosynthesis. Up-regulated by far red light in elongating petioles. Not regulated by a circadian clock. Mutation of GA20ox2 delays flowering.

BLAEV0015718	TFU-TNA_sweep_both, TBU-TNA_sweep_pop1only, TNA-TDS_sweep_pop2only	AT5G51820	Encodes a plastid isoform of the enzyme phosphoglucomutase involved in controlling photosynthetic carbon flow. Effective petiole movement against the direction of the gravity requires functional PGM activity that is required for full development of amyloplasts.
BLAEV0015728	TFU-TDS_sweep_pop1only, TBU-TDS_sweep_pop1only, TBU-TRA_sweep_pop1only, TPR-TNA_rhodxy, TPR-TNA_sweep_both, TFU-TPR_sweep_both, TBU-TPR_rhodxy, TBU-TPR_sweep_both, TNA-TDS_sweep_pop1only, TNA-TRA_sweep_pop1only	AT5G51890	encodes peroxidase involved in the lignification of tracheary elements (TE) in roots
BLAEV0030877	TNA-TDS_sweep_pop1only	AT5G52030	TraB family protein;(source:Araport11)
BLAEV0015730	TFU-TNA_sweep_both	AT5G52070	Agenet domain-containing protein;(source:Araport11)
BLAEV0015732	TPR-TRA_sweep_pop1only	AT5G52120	phloem protein 2-A14;(source:Araport11)
BLAEV0015753	TFU-TNA_sweep_both, TFU-TDS_rhodxy, TFU-TDS_sweep_both, TFU-TBU_rhodxy, TFU-TBU_sweep_both	AT5G52240	Encodes a protein with similarity to progesterone-binding proteins in animals. Has been shown to bind steroids in vitro. Expressed in aerial portions of the plant excluding mature flowers and siliques. Antisense experiments suggest a role in inhibition of hypocotyl cell elongation. Expression is suppressed light grown seedlings transferred to the dark. The mRNA is cell-to-cell mobile.
BLAEV0015765	TPR-TDS_sweep_pop1only	AT5G52380	N/A
BLAEV0015784	TFU-TNA_sweep_both	AT5G52450	MATE efflux family protein;(source:Araport11)
BLAEV0015794	TFU-TNA_sweep_pop2only	AT5G52520	Encodes a chloroplast and mitochondria localized prolyl-tRNA synthetase.
BLAEV0015847	TPR-TRA_sweep_pop2only	AT5G52990	SNARE-like superfamily protein;(source:Araport11)
BLAEV0015845	TPR-TRA_sweep_pop2only	AT5G53010	calcium-transporting ATPase;(source:Araport11)
BLAEV0016031	TPR-TNA_sweep_pop2only	AT5G54310	A member of ARF GAP domain (AGD); A thaliana has 15 members, grouped into four classes. Regulates membrane trafficking and organ separation.
BLAEV0016609	TFU-TDS_sweep_pop2only	AT5G58260	Encodes subunit NDH-N of NAD(P)H:plastoquinone dehydrogenase complex (Ndh complex) present in the thylakoid membrane of chloroplasts. This subunit is thought to be required for Ndh complex assembly.
BLAEV0016613	TFU-TDS_sweep_pop2only	AT5G58270	Encodes a mitochondrial half-molecule ABC transporter; a member of ATM subfamily. Mutants are dwarfed, chlorotic plants with altered leaf morphology. ATM3 transcription is induced by Cd(II) or Pb(II). Involved in heavy metal resistance. Arabidopsis thaliana has three ATM genes, namely ATM1, ATM2 and ATM3. Only ATM3 has an important function for plant growth. Role in Moco biosynthesis.
BLAEV0016732	TBU-TDS_sweep_pop1only	AT5G59260	Concanavalin A-like lectin protein kinase family protein;(source:Araport11)
BLAEV0036288	TNA-TRA_sweep_pop1only	AT5G61350	Encodes a membrane-localized receptor-like kinase that regulates root hair tip growth by maintaining cytoplasmic Ca2+ gradients. Knockouts of CAP1 produced more cytoplasmic NH4+ and ceased growth of root hairs on MS medium except when NH4+ was depleted; NH4+ depletion reestablished the Ca2+ gradient necessary for normal growth. The lower net NH4+ influx across the vacuolar membrane and relatively alkaline cytosolic pH of root hairs in cap1-1 relative to wild type

			implied that mutation of CAP1 results in more NH4+ accumulation in the cytoplasm. Furthermore; CAP1 functionally complemented npr1 kinase yeast mutant defective in high-affinity NH4+ uptake via MEP2, distinguishing CAP1 as a cytosolic modulator of NH4+ level that participates in NH4+ homeostasis-regulated root hair growth by modulating tip-focused cytoplasmic Ca2+ gradients.
BLAEV0038026	TPR-TRA_sweep_pop1only	AT5G61820	P1-interacting host factor required for robust virus infection.
BLAEV0030850	TBU-TRA_sweep_pop1only, TPR-TDS_sweep_pop2only, TBU-TPR_sweep_pop1only	AT5G62600	Encodes a nuclear importer of serine-arginine rich (SR) proteins and is involved in the regulation of splicing of R genes by regulating the import of the SR proteins into the nucleus.
BLAEV0030852	TBU-TRA_sweep_pop1only, TPR-TDS_sweep_pop2only, TBU-TPR_sweep_pop1only	AT5G62610	basic helix-loop-helix (bHLH) DNA-binding superfamily protein;(source:Araport11)
BLAEV0030923	TPR-TRA_sweep_pop1only	AT5G63080	Encodes a HR demethylase that acts as a positive regulator of seed germination in the PHYB-PIL5-SOM pathway.
BLAEV0030987	TNA-TDS_sweep_pop1only	AT5G63670	Transcription elongation factor SPT4 homolog 2. T-DNA mutant is more susceptible to the biotroph Hpa.
BLAEV0042906	TNA-TDS_sweep_pop1only	AT5G65165	One of three isoforms of the iron-sulfur component of the succinate dehydrogenase complex; a component of the mitochondrial respiratory chain complex II. The product of the nuclear encoded gene is imported into the mitochondrion. Transcripts appear during seed maturation, persist through desiccation, are abundant in dry seeds, and markedly decline during germination.
BLAEV0044620	TBU-TNA_rhodxy, TBU-TNA_sweep_both, TPR-TNA_rhodxy, TPR-TNA_sweep_both, TNA-TRA_rhodxy, TNA-TRA_sweep_both	AT5G66620	DA1-related protein 6;(source:Araport11)
BLAEV0000640	TPR-TRA_sweep_pop2only	NA	
BLAEV0000642	TPR-TRA_sweep_pop2only	NA	
BLAEV0000643	TPR-TRA_sweep_pop2only	NA	
BLAEV0000644	TPR-TRA_sweep_pop2only	NA	
BLAEV0001107	TFU-TNA_sweep_pop2only	NA	
BLAEV0002036	TNA-TDS_rhodxy	NA	
BLAEV0002664	TFU-TBU_sweep_pop2only	NA	
BLAEV0002874	TFU-TRA_sweep_both, TBU-TRA_sweep_pop2only, TPR-TRA_sweep_pop2only, TFU-TBU_sweep_both	NA	
BLAEV0002882	TFU-TBU_sweep_both	NA	
BLAEV0003354	TFU-TBU_sweep_pop2only	NA	
BLAEV0003995	TPR-TNA_rhodxy, TPR-TNA_sweep_pop1only, TPR-TDS_sweep_pop1only	NA	

BLAEV0003996	TPR-TNA_rhodxy, TPR-TNA_sweep_pop1only, TPR-TDS_sweep_pop1only	NA
BLAEV0003997	TPR-TNA_rhodxy, TPR-TNA_sweep_pop1only, TPR-TDS_sweep_pop1only	NA
BLAEV0004088	TPR-TNA_sweep_pop1only, TNA-TDS_sweep_pop2only	NA
BLAEV0004089	TPR-TNA_sweep_pop1only, TNA-TDS_sweep_pop2only	NA
BLAEV0004196	TFU-TDS_sweep_pop2only, TFU-TBU_sweep_pop2only	NA
BLAEV0004223	TFU-TBU_sweep_pop2only	NA
BLAEV0004224	TFU-TBU_sweep_pop2only	NA
BLAEV0004448	TPR-TNA_sweep_pop2only	NA
BLAEV0004561	TFU-TNA_sweep_pop2only	NA
BLAEV0004562	TFU-TNA_sweep_pop2only	NA
BLAEV0004563	TFU-TNA_sweep_pop2only	NA
BLAEV0004893	TBU-TDS_sweep_both	NA
BLAEV0004898	TBU-TRA_sweep_pop1only	NA
BLAEV0004899	TBU-TRA_sweep_pop1only	NA
BLAEV0005309	TBU-TDS_sweep_both	NA
BLAEV0006087	TFU-TBU_sweep_pop2only	NA
BLAEV0006434	TPR-TNA_sweep_pop2only	NA
BLAEV0006436	TPR-TNA_sweep_pop2only	NA
BLAEV0006527	TBU-TRA_sweep_pop1only, TBU-TPR_sweep_pop1only	NA
BLAEV0006749	TBU-TRA_sweep_pop1only	NA
BLAEV0007219	TBU-TDS_sweep_pop1only	NA
BLAEV0007740	TBU-TNA_sweep_pop1only, TFU-TBU_sweep_pop2only	NA
BLAEV0007747	TFU-TBU_sweep_pop2only	NA
BLAEV0007922	TNA-TRA_sweep_pop1only	NA
BLAEV0009896	TFU-TNA_sweep_pop2only	NA
BLAEV0010040	TBU-TRA_sweep_pop1only	NA
BLAEV0010042	TBU-TRA_sweep_pop1only	NA
BLAEV0012680	TFU-TNA_sweep_both	NA
BLAEV0013453	TBU-TRA_sweep_pop2only	NA
BLAEV0013518	TBU-TDS_sweep_pop1only	NA
BLAEV0013519	TBU-TDS_sweep_pop1only	NA
BLAEV0013521	TBU-TDS_rhodxy, TBU-TDS_sweep_both, TNA-	NA

	TDS_sweep_both, TDS-TRA_sweep_pop2only	
BLAEV0013524	TBU-TDS_rhodxy, TBU-TDS_sweep_both	NA
BLAEV0013527	TPR-TDS_sweep_pop2only, TNA-TDS_sweep_pop2only, TDS-TRA_sweep_pop1only	NA
BLAEV0013528	TPR-TDS_sweep_pop2only, TNA-TDS_sweep_pop2only, TDS-TRA_sweep_pop1only	NA
BLAEV0013557	TNA-TRA_sweep_pop1only	NA
BLAEV0013714	TPR-TRA_sweep_pop1only	NA
BLAEV0013730	TNA-TRA_sweep_pop1only	NA
BLAEV0013761	TPR-TRA_sweep_pop2only	NA
BLAEV0014517	TPR-TNA_sweep_pop2only	NA
BLAEV0014575	TBU-TDS_sweep_pop1only	NA
BLAEV0014576	TBU-TDS_sweep_pop1only	NA
BLAEV0014690	TBU-TPR_sweep_pop1only	NA
BLAEV0014725	TBU-TDS_sweep_pop1only	NA
BLAEV0014827	TPR-TDS_sweep_pop1only	NA
BLAEV0014850	TBU-TPR_sweep_pop1only	NA
BLAEV0014852	TBU-TPR_sweep_pop1only	NA
BLAEV0014860	TBU-TNA_sweep_pop1only	NA
BLAEV0014861	TBU-TNA_sweep_pop1only	NA
BLAEV0014863	TBU-TNA_sweep_pop1only	NA
BLAEV0014917	TBU-TPR_sweep_pop2only	NA
BLAEV0014934	TBU-TNA_sweep_pop2only, TPR-TNA_sweep_pop2only, TNA-TDS_rhodxy, TNA-TDS_sweep_both	NA
BLAEV0014935	TBU-TNA_sweep_pop2only, TPR-TNA_sweep_pop2only, TNA-TDS_rhodxy, TNA-TDS_sweep_both	NA
BLAEV0014967	TPR-TNA_sweep_pop2only	NA
BLAEV0015012	TPR-TRA_sweep_pop2only	NA
BLAEV0015013	TPR-TRA_sweep_pop2only	NA
BLAEV0015014	TPR-TRA_sweep_pop2only	NA
BLAEV0015016	TFU-TRA_sweep_pop1only, TBU-TRA_sweep_both, TPR-TRA_rhodxy, TPR-	NA

	TRA_sweep_both, TDS-TRA_sweep_both	
BLAEV0015025	TFU-TNA_sweep_pop2only	NA
BLAEV0015032	TPR-TDS_sweep_pop1only	NA
BLAEV0015051	TFU-TNA_sweep_pop2only	NA
BLAEV0015052	TFU-TNA_sweep_pop2only	NA
BLAEV0015210	TBU-TDS_sweep_pop1only	NA
BLAEV0015298	TFU-TNA_sweep_both, TBU-TRA_sweep_both	NA
BLAEV0015320	TPR-TDS_sweep_pop1only	NA
BLAEV0015387	TFU-TDS_sweep_pop1only, TFU-TBU_sweep_pop1only	NA
BLAEV0015418	TFU-TNA_sweep_pop1only	NA
BLAEV0015420	TFU-TNA_sweep_pop1only, TFU-TRA_sweep_pop1only	NA
BLAEV0015424	TFU-TBU_sweep_pop2only	NA
BLAEV0015426	TFU-TBU_sweep_pop2only	NA
BLAEV0015427	TFU-TBU_sweep_pop2only	NA
BLAEV0015526	TFU-TNA_rhodxy, TFU-TNA_sweep_both	NA
BLAEV0015558	TPR-TNA_sweep_pop2only, TNA-TRA_sweep_both	NA
BLAEV0015565	TNA-TRA_rhodxy, TNA-TRA_sweep_both	NA
BLAEV0015653	TFU-TNA_sweep_pop2only	NA
BLAEV0015668	TPR-TNA_sweep_pop1only	NA
BLAEV0015682	TBU-TDS_sweep_pop2only	NA
BLAEV0015704	TFU-TNA_rhodxy, TFU-TNA_sweep_both, TFU-TBU_rhodxy, TFU-TBU_sweep_pop2only	NA
BLAEV0015710	TFU-TNA_sweep_pop2only	NA
BLAEV0015731	TFU-TNA_sweep_both	NA
BLAEV0015782	TFU-TNA_sweep_both	NA
BLAEV0015846	TPR-TRA_sweep_pop2only	NA
BLAEV0016347	TBU-TRA_sweep_pop1only	NA
BLAEV0016611	TFU-TDS_sweep_pop2only	NA
BLAEV0017166	TBU-TDS_sweep_pop1only, TNA-TDS_sweep_pop1only	NA
BLAEV0018279	TFU-TDS_sweep_pop2only	NA
BLAEV0018778	TBU-TDS_sweep_pop1only	NA

BLAEV0020591	TBU-TRA_sweep_pop1only, TPR-TRA_sweep_pop1only	NA
BLAEV0020736	TFU-TDS_sweep_pop2only	NA
BLAEV0020746	TPR-TRA_sweep_both	NA
BLAEV0020747	TPR-TRA_sweep_both	NA
BLAEV0020881	TBU-TRA_sweep_pop2only	NA
BLAEV0020882	TBU-TRA_sweep_pop2only	NA
BLAEV0020891	TPR-TRA_sweep_pop1only	NA
BLAEV0020905	TBU-TNA_sweep_pop2only, TNA-TDS_sweep_pop1only	NA
BLAEV0021231	TBU-TRA_sweep_both	NA
BLAEV0021262	TPR-TRA_sweep_pop2only	NA
BLAEV0021263	TFU-TRA_sweep_pop2only, TPR-TRA_sweep_pop2only	NA
BLAEV0021496	TBU-TRA_sweep_both	NA
BLAEV0021606	TFU-TNA_sweep_pop2only	NA
BLAEV0021619	TBU-TDS_rhodxy	NA
BLAEV0021683	TBU-TPR_sweep_both	NA
BLAEV0022184	TBU-TNA_sweep_pop1only	NA
BLAEV0025000	TBU-TDS_sweep_pop2only, TFU-TBU_sweep_pop2only	NA
BLAEV0027053	TPR-TNA_sweep_both, TNA-TDS_sweep_pop1only	NA
BLAEV0027056	TNA-TRA_sweep_both	NA
BLAEV0027495	TPR-TRA_rhodxy, TPR-TRA_sweep_both	NA
BLAEV0027858	TBU-TRA_sweep_both, TDS-TRA_sweep_pop2only	NA
BLAEV0027974	TBU-TDS_sweep_pop1only, TBU-TNA_sweep_both, TFU-TBU_sweep_pop2only	NA
BLAEV0028214	TBU-TRA_sweep_pop1only	NA
BLAEV0028233	TBU-TNA_sweep_pop1only	NA
BLAEV0028235	TBU-TNA_sweep_pop1only	NA
BLAEV0028259	TFU-TNA_sweep_pop2only	NA
BLAEV0028260	TFU-TNA_sweep_pop2only	NA
BLAEV0028308	TBU-TNA_sweep_pop2only	NA
BLAEV0028748	TBU-TPR_sweep_pop1only	NA
BLAEV0028865	TBU-TNA_sweep_pop1only, TBU-TRA_sweep_pop1only, TPR-TDS_sweep_pop2only, TNA-	NA

	TDS_sweep_pop2only, TDS-TRA_sweep_pop1only	
BLAEV0028872	TBU-TRA_sweep_both, TBU-TPR_sweep_pop1only	NA
BLAEV0029449	TBU-TDS_sweep_pop2only, TBU-TNA_sweep_pop2only	NA
BLAEV0029451	TBU-TDS_sweep_pop2only	NA
BLAEV0029899	TFU-TPR_sweep_both	NA
BLAEV0030009	TBU-TDS_rhodxy, TBU-TDS_sweep_both, TBU-TNA_sweep_both, TFU-TBU_rhodxy, TFU-TBU_sweep_both	NA
BLAEV0030014	TBU-TDS_rhodxy, TBU-TDS_sweep_both, TBU-TNA_sweep_both, TBU-TRA_sweep_pop1only, TFU-TBU_rhodxy, TFU-TBU_sweep_both	NA
BLAEV0030027	TBU-TDS_sweep_both, TBU-TNA_sweep_both, TBU-TRA_sweep_pop1only	NA
BLAEV0030029	TBU-TDS_rhodxy, TBU-TDS_sweep_both, TBU-TNA_rhodxy, TBU-TNA_sweep_both, TFU-TBU_rhodxy, TFU-TBU_sweep_both	NA
BLAEV0030035	TBU-TDS_rhodxy, TBU-TDS_sweep_both, TBU-TNA_rhodxy, TBU-TNA_sweep_both, TFU-TBU_rhodxy, TFU-TBU_sweep_both	NA
BLAEV0030040	TBU-TDS_sweep_both, TBU-TNA_sweep_both	NA
BLAEV0030041	TBU-TNA_sweep_both, TFU-TBU_sweep_pop2only, TBU-TPR_sweep_pop1only	NA
BLAEV0030045	TBU-TDS_sweep_both, TBU-TNA_sweep_both, TFU-TBU_sweep_pop2only, TBU-TPR_sweep_pop1only	NA

BLAEV0030047	TBU-TDS_sweep_both, TBU-TNA_sweep_both, TFU-TBU_sweep_pop2only, TBU-TPR_sweep_pop1only	NA
BLAEV0030223	TBU-TDS_rhodxy, TBU-TDS_sweep_both, TBU-TNA_rhodxy, TBU-TNA_sweep_pop2only, TFU-TBU_rhodxy	NA
BLAEV0030225	TBU-TDS_rhodxy, TBU-TDS_sweep_both, TBU-TNA_rhodxy, TBU-TNA_sweep_pop2only, TFU-TBU_rhodxy	NA
BLAEV0030237	TBU-TNA_sweep_pop1only	NA
BLAEV0030293	TBU-TPR_sweep_pop1only	NA
BLAEV0030410	TPR-TRA_sweep_pop2only	NA
BLAEV0030851	TBU-TRA_sweep_pop1only, TPR-TDS_sweep_pop2only, TBU-TPR_sweep_pop1only	NA
BLAEV0030878	TNA-TDS_sweep_pop1only	NA
BLAEV0031187	TFU-TDS_sweep_both, TPR-TDS_sweep_pop1only, TNA-TDS_sweep_pop1only	NA
BLAEV0031188	TFU-TDS_sweep_both, TNA-TDS_sweep_pop1only	NA
BLAEV0031189	TFU-TDS_sweep_both, TPR-TDS_sweep_pop1only, TNA-TDS_sweep_pop1only	NA
BLAEV0031190	TFU-TDS_sweep_both, TPR-TDS_sweep_pop1only, TNA-TDS_sweep_pop1only	NA
BLAEV0031196	TFU-TDS_sweep_both, TBU-TNA_sweep_pop2only, TPR-TDS_sweep_pop1only, TNA-TDS_sweep_both	NA
BLAEV0031204	TFU-TDS_rhodxy, TFU-TDS_sweep_both, TBU-TNA_sweep_pop2only, TPR-TDS_sweep_both, TNA-TDS_rhodxy, TNA-TDS_sweep_both, TNA-TRA_sweep_pop1only	NA

BLAEV0031211	TFU-TDS_sweep_pop2only	NA
BLAEV0031235	TPR-TDS_sweep_pop1only	NA
BLAEV0031236	TPR-TDS_sweep_pop1only	NA
BLAEV0031266	TPR-TNA_sweep_pop2only, TPR-TDS_sweep_both, TBU-TPR_sweep_pop1only, TNA-TRA_sweep_pop1only	NA
BLAEV0031281	TFU-TDS_sweep_both, TBU-TNA_sweep_pop2only, TFU-TBU_sweep_pop1only	NA
BLAEV0031296	TFU-TDS_rhodxy, TFU-TDS_sweep_pop1only, TFU-TDS_sweep_both, TFU-TRA_sweep_both, TPR-TNA_sweep_pop2only, TFU-TBU_sweep_pop1only, TNA-TDS_sweep_pop1only, TNA-TRA_rhodxy, TNA-TRA_sweep_both	NA
BLAEV0031312	TFU-TDS_sweep_both	NA
BLAEV0031382	TFU-TDS_sweep_pop1only	NA
BLAEV0031420	TFU-TDS_sweep_pop2only	NA
BLAEV0031500	TFU-TRA_sweep_pop1only, TFU-TBU_sweep_pop1only	NA
BLAEV0031559	TBU-TNA_sweep_pop1only	NA
BLAEV0031560	TBU-TNA_sweep_pop1only	NA
BLAEV0032494	TPR-TNA_sweep_pop1only	NA
BLAEV0032540	TBU-TRA_sweep_pop1only, TNA-TRA_sweep_pop1only	NA
BLAEV0032929	TNA-TDS_sweep_pop1only	NA
BLAEV0032932	TBU-TDS_sweep_pop1only	NA
BLAEV0032933	TBU-TDS_sweep_pop1only, TNA-TDS_sweep_pop1only	NA
BLAEV0033436	TPR-TRA_sweep_pop2only	NA
BLAEV0033437	TPR-TRA_sweep_pop2only	NA
BLAEV0033483	TBU-TRA_sweep_pop1only, TBU-TPR_rhodxy, TBU-TPR_sweep_both	NA
BLAEV0034503	TPR-TNA_sweep_pop2only	NA
BLAEV0034505	TPR-TNA_sweep_pop2only	NA
BLAEV0034853	TBU-TRA_sweep_pop1only	NA

BLAEV0035877	TBU-TRA_sweep_pop1only, TPR-TRA_sweep_pop1only	NA
BLAEV0035879	TBU-TRA_sweep_pop1only, TPR-TRA_sweep_pop1only	NA
BLAEV0036282	TNA-TRA_sweep_pop1only	NA
BLAEV0036284	TNA-TRA_sweep_pop1only	NA
BLAEV0036737	TFU-TDS_sweep_pop2only	NA
BLAEV0037293	TBU-TDS_sweep_pop2only	NA
BLAEV0037382	TBU-TRA_sweep_both, TDS-TRA_sweep_pop2only	NA
BLAEV0037385	TPR-TRA_sweep_pop2only	NA
BLAEV0037572	TBU-TDS_sweep_pop2only	NA
BLAEV0037584	TBU-TDS_sweep_both, TBU-TNA_sweep_both, TFU-TBU_sweep_both, TBU-TPR_sweep_pop1only	NA
BLAEV0037590	TBU-TDS_sweep_pop1only	NA
BLAEV0037854	TPR-TRA_sweep_both, TPR-TNA_sweep_pop2only, TBU-TPR_sweep_both	NA
BLAEV0038576	TPR-TNA_sweep_both, TBU-TPR_sweep_both	NA
BLAEV0038578	TBU-TPR_sweep_pop1only	NA
BLAEV0038579	TPR-TRA_sweep_pop1only, TPR-TNA_rhodxy, TPR-TNA_sweep_both, TPR-TDS_rhodxy, TPR-TDS_sweep_both, TBU-TPR_rhodxy, TBU-TPR_sweep_both	NA
BLAEV0038618	TBU-TPR_sweep_pop2only	NA
BLAEV0041371	TPR-TRA_sweep_pop2only	NA
BLAEV0041476	TDS-TRA_sweep_pop1only	NA
BLAEV0041515	TPR-TDS_sweep_pop2only, TDS-TRA_sweep_pop1only	NA
BLAEV0041533	TPR-TDS_sweep_pop2only, TDS-TRA_sweep_pop1only	NA
BLAEV0041535	TFU-TRA_sweep_pop1only, TPR-TDS_sweep_pop2only, TDS-TRA_sweep_pop1only	NA
BLAEV0041538	TPR-TDS_sweep_pop2only, TDS-TRA_sweep_pop1only	NA

BLAEV0041539	TPR-TDS_sweep_pop2only, TDS-TRA_sweep_pop1only	NA
BLAEV0041543	TPR-TDS_sweep_pop2only, TDS-TRA_rhodxy, TDS-TRA_sweep_pop1only	NA
BLAEV0041545	TBU-TDS_sweep_pop2only, TPR-TDS_sweep_pop2only	NA
BLAEV0041561	TPR-TDS_sweep_pop2only, TDS-TRA_sweep_pop1only	NA
BLAEV0041796	TBU-TRA_sweep_pop1only	NA
BLAEV0041993	TFU-TDS_sweep_pop2only, TBU-TDS_sweep_pop2only	NA
BLAEV0042143	TFU-TDS_sweep_pop2only	NA
BLAEV0042375	TFU-TNA_sweep_pop2only, TFU-TDS_sweep_pop2only	NA
BLAEV0042420	TFU-TDS_sweep_both, TFU-TRA_sweep_pop1only, TNA-TDS_sweep_pop1only	NA
BLAEV0042421	TFU-TDS_sweep_both, TPR-TNA_sweep_pop2only, TNA-TDS_sweep_pop1only	NA
BLAEV0042450	TFU-TNA_sweep_pop1only	NA
BLAEV0042451	TFU-TPR_sweep_both	NA
BLAEV0042554	TPR-TRA_sweep_pop1only	NA
BLAEV0043312	TFU-TDS_rhodxy, TFU-TDS_sweep_pop2only, TBU-TDS_sweep_pop2only	NA
BLAEV0043313	TFU-TDS_rhodxy, TFU-TDS_sweep_pop2only, TBU-TDS_sweep_pop2only	NA
BLAEV0043496	TBU-TNA_rhodxy, TBU-TNA_sweep_both, TNA-TDS_sweep_pop1only	NA
BLAEV0043498	TBU-TNA_rhodxy, TBU-TNA_sweep_both	NA
BLAEV0043499	TBU-TNA_rhodxy, TBU-TNA_sweep_both, TNA-TDS_sweep_pop1only	NA
BLAEV0043500	TBU-TNA_rhodxy, TBU-TNA_sweep_both, TNA-TDS_sweep_pop1only	NA

BLAEV0043506	TBU-TNA_sweep_both, TBU-TPR_sweep_pop2only, TNA-TRA_sweep_pop1only	NA
BLAEV0043613	TBU-TDS_sweep_pop2only	NA
BLAEV0043744	TPR-TDS_sweep_pop2only	NA
BLAEV0043949	TBU-TNA_rhodxy, TBU-TNA_sweep_both, TFU-TBU_sweep_pop2only	NA
BLAEV0043983	TBU-TDS_rhodxy, TBU-TDS_sweep_both	NA
BLAEV0043984	TBU-TDS_rhodxy, TBU-TDS_sweep_both	NA
BLAEV0043991	TFU-TBU_sweep_both	NA
BLAEV0044158	TBU-TNA_sweep_both	NA
BLAEV0044173	TBU-TNA_sweep_both, TNA-TDS_sweep_pop1only	NA
BLAEV0044321	TPR-TRA_sweep_pop1only	NA
BLAEV0044375	TBU-TRA_sweep_both, TBU-TPR_sweep_pop1only	NA
BLAEV0044377	TBU-TDS_sweep_pop1only, TBU-TNA_sweep_pop1only	NA
BLAEV0044378	TBU-TDS_sweep_pop1only, TBU-TNA_sweep_pop1only	NA
BLAEV0044379	TBU-TDS_sweep_pop1only, TBU-TNA_sweep_pop1only	NA
BLAEV0044381	TFU-TBU_rhodxy, TFU-TBU_sweep_pop1only, TNA-TRA_rhodxy, TNA-TRA_sweep_pop1only	NA
BLAEV0044382	TBU-TDS_sweep_both, TBU-TNA_rhodxy, TBU-TNA_sweep_both, TPR-TNA_sweep_both, TFU-TBU_rhodxy, TFU-TBU_sweep_both, TNA-TRA_sweep_both	NA
BLAEV0044385	TBU-TDS_sweep_pop2only, TBU-TRA_sweep_pop2only	NA
BLAEV0044399	TFU-TDS_sweep_pop2only	NA
BLAEV0044400	TFU-TDS_sweep_pop2only	NA
BLAEV0044621	TBU-TNA_rhodxy, TBU-TNA_sweep_both, TPR-	NA

	TNA_rhodxy, TPR-TNA_sweep_both, TNA-TRA_rhodxy, TNA-TRA_sweep_both	
BLAEV0044624	TPR-TNA_rhodxy, TPR-TNA_sweep_both	NA
BLAEV0044627	TNA-TRA_rhodxy, TNA-TRA_sweep_both	NA
BLAEV0044628	TPR-TNA_rhodxy, TPR-TNA_sweep_both	NA
BLAEV0044638	TBU-TNA_sweep_both, TFU-TBU_sweep_pop2only	NA
BLAEV0045882	TBU-TPR_sweep_pop1only	NA
BLAEV0046191	TBU-TRA_sweep_pop1only	NA
BLAEV0046614	TDS-TRA_sweep_pop2only	NA
BLAEV0046982	TBU-TDS_rhodxy, TBU-TDS_sweep_both, TPR-TDS_rhodxy, TPR-TDS_sweep_both	NA
BLAEV0046994	TBU-TDS_sweep_pop2only, TBU-TPR_sweep_pop2only	NA
BLAEV0047843	TBU-TDS_sweep_both	NA
BLAEV0047844	TBU-TDS_sweep_both	NA
BLAEV0048965	TBU-TDS_sweep_pop1only	NA
BLAEV0049301	TFU-TDS_sweep_pop2only	NA
BLAEV0049757	TFU-TDS_rhodxy, TFU-TDS_sweep_both	NA
BLAEV0049759	TFU-TDS_rhodxy, TFU-TDS_sweep_both	NA
BLAEV0049762	TFU-TDS_rhodxy, TFU-TDS_sweep_both	NA
BLAEV0049763	TFU-TDS_rhodxy, TFU-TDS_sweep_both	NA
BLAEV0050697	TPR-TNA_rhodxy	NA
BLAEV0051647	TBU-TNA_sweep_both, TPR-TNA_sweep_both	NA
BLAEV0051785	TFU-TNA_sweep_pop2only	NA
BLAEV0051997	TBU-TDS_rhodxy, TBU-TDS_sweep_both, TBU-TNA_rhodxy, TBU-TNA_sweep_pop2only, TFU-TBU_rhodxy, TFU-	NA

	TBU_sweep_both, TNA-TRA_rhodxy, TNA-TRA_sweep_both

Table S8 - Gene ontology terms significantly enriched in tetraploids

Gene ontology terms significantly enriched in tetraploids of *Biscutella laevigata*.

Type	GO.ID	Term	Annotated	Significant	Expected	topgoFisher
Rho and d_{XY}	GO:0080003	thalianol metabolic process	1	1	0	0.00084
	GO:0052696	flavonoid glucuronidation	3	1	0	0.00253
	GO:0080164	regulation of nitric oxide metabolic process	4	1	0	0.00337
	GO:0009909	regulation of flower development	405	3	0.34	0.00475
	GO:0031048	regulatory ncRNA-mediated heterochromatin formation	128	2	0.11	0.00524
	GO:0006346	DNA methylation-dependent heterochromatin formation	145	2	0.12	0.00667
	GO:0048255	mRNA stabilization	10	1	0.01	0.00841
	GO:0010167	response to nitrate	169	2	0.14	0.00896
	GO:0033617	mitochondrial cytochrome c oxidase assembly	12	1	0.01	0.01008
	GO:0009298	GDP-mannose biosynthetic process	12	1	0.01	0.01008
	GO:0034551	mitochondrial respiratory chain complex III assembly	13	1	0.01	0.01092
	GO:1902456	regulation of stomatal opening	14	1	0.01	0.01175
	GO:0031507	heterochromatin formation	198	3	0.17	0.01184
	GO:0006002	fructose 6-phosphate metabolic process	24	1	0.02	0.02006
	GO:0061615	glycolytic process through fructose-6-phosphate	24	1	0.02	0.02006
	GO:0010152	pollen maturation	25	1	0.02	0.02089
	GO:0006086	acetyl-CoA biosynthetic process from pyruvate	32	1	0.03	0.02666
	GO:0046482	para-aminobenzoic acid metabolic process	32	1	0.03	0.02666
	GO:0006885	regulation of pH	36	1	0.03	0.02995
	GO:0009269	response to desiccation	37	1	0.03	0.03077
	GO:0008216	spermidine metabolic process	38	1	0.03	0.03159
	GO:0035725	sodium ion transmembrane transport	44	1	0.04	0.03649
	GO:0008356	asymmetric cell division	51	1	0.04	0.04217
	GO:0048510	regulation of timing of transition from vegetative to reproductive phase	52	1	0.04	0.04298
	GO:0001731	formation of translation preinitiation complex	55	1	0.05	0.0454
	GO:0006596	polyamine biosynthetic process	56	1	0.05	0.04621
Sweep	GO:0006817	phosphate ion transport	82	4	0.47	0.0013
	GO:0007165	signal transduction	4000	27	22.87	0.0033
	GO:0006361	transcription initiation at RNA polymerase I promoter	1	1	0.01	0.0057
	GO:0080003	thalianol metabolic process	1	1	0.01	0.0057
	GO:0006446	regulation of translational initiation	68	3	0.39	0.007
	GO:1902358	sulfate transmembrane transport	24	2	0.14	0.0083
	GO:0031640	killing of cells of another organism	150	4	0.86	0.011
	GO:2000031	regulation of salicylic acid mediated signaling pathway	28	2	0.16	0.0111
	GO:0019370	leukotriene biosynthetic process	2	1	0.01	0.0114
	GO:0006311	meiotic gene conversion	2	1	0.01	0.0114

GO:0080160	selenate transport	2	1	0.01	0.0114
GO:0046482	para-aminobenzoic acid metabolic process	32	2	0.18	0.0144
GO:0006952	defense response	2619	24	14.97	0.0147
GO:0002181	cytoplasmic translation	152	5	0.87	0.0165
GO:0006433	prolyl-tRNA aminoacylation	3	1	0.02	0.0171
GO:0052696	flavonoid glucuronidation	3	1	0.02	0.0171
GO:0003400	regulation of COPII vesicle coating	3	1	0.02	0.0171
GO:1902184	negative regulation of shoot apical meristem development	3	1	0.02	0.0171
GO:0030422	siRNA processing	140	3	0.8	0.0199
GO:0048449	floral organ formation	166	4	0.95	0.0224
GO:0045128	negative regulation of reciprocal meiotic recombination	4	1	0.02	0.0227
GO:1901684	arsenate ion transmembrane transport	4	1	0.02	0.0227
GO:0043171	peptide catabolic process	4	1	0.02	0.0227
GO:0080164	regulation of nitric oxide metabolic process	4	1	0.02	0.0227
GO:0000494	box C/D sno(s)RNA 3'-end processing	4	1	0.02	0.0227
GO:0017186	peptidyl-pyroglutamic acid biosynthetic process, using glutaminyl-peptide cyclotransferase	4	1	0.02	0.0227
GO:0006290	pyrimidine dimer repair	5	1	0.03	0.0283
GO:0006734	NADH metabolic process	6	1	0.03	0.0338
GO:0022414	reproductive process	3220	17	18.41	0.0342
GO:0008356	asymmetric cell division	51	2	0.29	0.0345
GO:0031048	regulatory ncRNA-mediated heterochromatin formation	128	3	0.73	0.0374
GO:0034051	negative regulation of plant-type hypersensitive response	7	1	0.04	0.0393
GO:0046355	mannan catabolic process	7	1	0.04	0.0393
GO:0080140	regulation of jasmonic acid metabolic process	7	1	0.04	0.0393
GO:0001731	formation of translation preinitiation complex	55	2	0.31	0.0396
GO:0006260	DNA replication	487	4	2.78	0.0432
GO:1990592	protein K69-linked ufmylation	8	1	0.05	0.0448
GO:0006107	oxaloacetate metabolic process	8	1	0.05	0.0448
GO:0006855	xenobiotic transmembrane transport	139	3	0.79	0.0458

Notes S1: Adaptation to whole-genome duplication (WGD) in *Biscutella laevigata.*
Disrupting multiple physiological processes, WGD is expected to induce significant fitness costs (Cavalier-Smith 2005; Clo and Kolář 2021). In particular, WGD-induced larger genome size (Beaulieu et al. 2008) leads to increased cell size underpinning nucleotypic effects and resulting in series of morphological, anatomical, developmental, phenological as well as physiological challenges that must be overcome by polyploids to establish and expand (Comai 2005). Another critical issue brought with WGD is proper chromosome segregation during meiosis (Ramsey and Schemske 2002) as the lack of pairing preference between the four homologous chromosomes coupled to multiple crossing over per chromosome can lead to the formation of multivalent associations, hence increasing the likelihood of missegregation or chromosome breakage (Bomblies and Madlung 2014; Bomblies et al. 2016). Adaptation to WGD has been investigated in *Arabidopsis arenosa* (Hollister et al. 2012; Yant et al. 2013; Wright et al. 2015; Bohutínská et al. 2021; Morgan et al. 2021; Gonzalo et al. 2023), with a particular focus on meiotic adaptation, where adaptation to WGD was at least partially acquired from adaptive gene flow with *A. lyrata* (Marburger et al. 2019). Insights from other angiosperm species are restricted to *Cardamine amara* (Bohutínská et al. 2021) and the *Cochlearia* species complex (Bray et al. 2024).
Genome scans contrasting diploid and tetraploid samples aimed at identifying loci involved in adaptation to WGD, encompassed 11,691 genomic windows that contained at least 1,000 covered sites and together spanned 90.5% of annotated gene models (Fig. T1). Out of a genome-wide rho differentiation of 0.124 between ploidal levels, 23 windows (0.20%) were identified as outliers for both rho and d_{XY} and contained 50 genes, 29 of which were retained after prioritization. In addition, 62 windows (0.53%) showed evidence of selective sweep in tetraploids, encompassing 65 genes with 32 retained after prioritization. Together these scans yielded a final list of 53 candidate genes putatively underlying adaptation to WGD (Table T1, Table T2). None of these genes overlapped with meiosis genes putatively under selection in autotetraploid populations of *Arabidopsis arenosa* and *A. lyrata* (Table T3). Although most of the 71 meiosis-related genes compiled by Yant et al. (Yant et al. 2013) were included in here analyzed windows, only one candidate gene overlapped (Tabe T4). The candidate gene overlapping with the list of meiosis-related genes is the orthologus of AT5G16270, which is involved in somatic DNA double strand break damage repair. Nevertheless, candidate adaptive genes to WGD in *B. laevigata* were enriched in GO terms related to metabolic processes such as fatty acid biosynthesis and amino acid biosynthesis, as well as processes typically associated with stress responses such as jasmonic acid biosynthesis (Tabe T5).

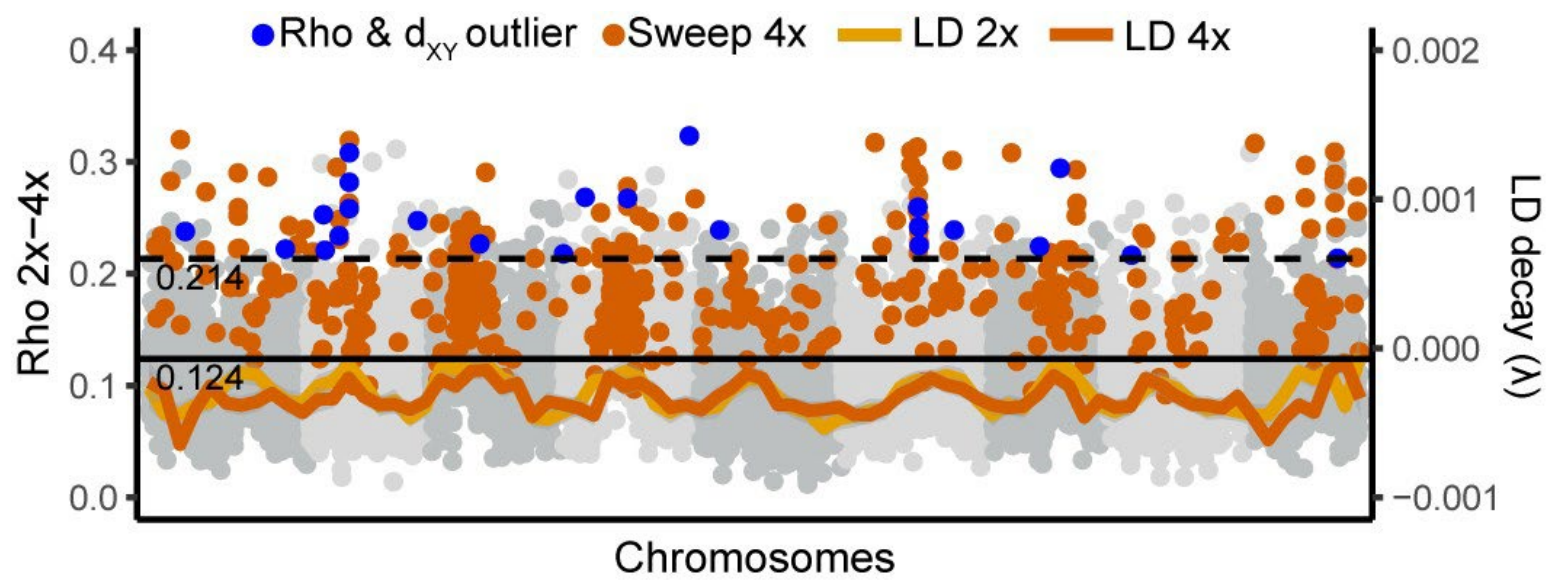

Fig. T1. Genome scans identifying signatures of selection between all diploid (2x) vs all tetraploid (4x) samples of *Biscutella laevigata*. Rho differentiation along chromosomes, with outlier windows for both relative differentiation (rho) and absolute divergence (d_{XY}) highlighted in blue. Loci putatively driven by a selective sweep in tetraploids are shown as windows presenting an outlier differentiation relative to their level of diversity in the tetraploids. Orange and yellow lines present the linkage disequilibrium (LD) decay (λ) in corresponding ploidal level.

Table T1. Number of genomic windows (w) and genes (g) analyzed and identified as loci presenting signatures of selection and number of candidate genes (cg) identified following gene prioritization as putatively underlying adaptation to whole-genome duplication (WGD) in tetraploid *Biscutella laevigata*

	**diploids-tetraploids**		
**Set of windows/genes**	**w**	**g**	**cg**
Analyzed (> 1000 covered sites)	11691	38126	-
Rho and d_{XY} outliers ¶	23	50	29
Sweep tetraploids#	62	65	32
Candidate genes	-	-	53

¶Loci identified as outlier for both rho and d_{XY}, supporting reduced gene flow by selection

#Loci showing excessive differentiation relative to their diversity, supporting a selective sweep in tetraploids

Table T2. Candidate genes underlying adaptation to whole-genome duplication in *Biscutella laevigata* and their putative functions based on orthologs in *Arabidopsis thaliana*.

**Gene**	**Ortholog**	**Description TAIR10**
BLAEV0051128	AT1G09380	Nodulin MtN21-like transporter family protein
BLAEV0051134	AT1G09390	GDSL-motif esterase/acyltransferase/lipase. Enzyme group with broad substrate specificity that may catalyze acyltransfer or hydrolase reactions with lipid and non-lipid substrates.
BLAEV0051133	AT1G09410	Pentatricopeptide (PPR) repeat-containing protein;(source:Araport11)

BLAEV0051131	AT1G09420	Encodes a protein similar to glucose-6-phosphate dehydrogenase but; based on amino acid differences in the active site and lack of activity, does not encode a functional G6PDH. The amino acid sequence for the consensus sequence of the G6PDH active site (DHYLGKE) differs in three places in this protein. gc exon splice site at 20574 is based on protein alignment, and is not confirmed experimentally.
BLAEV0051129	AT1G09480	Similar to Eucalyptus gunnii alcohol dehydrogenase of unknown physiological function (GI:1143445); Vigna unguiculata (gi:1854445), NOT a cinnamyl-alcohol dehydrogenase The mRNA is cell-to-cell mobile.
BLAEV0028341	AT1G56210	Heavy metal transport/detoxification superfamily protein;(source:Araport11)
BLAEV0025687	AT1G74950	Key regulator in alternative splicing in the jasmonate signaling pathway; alone and in collaboration with other regulators.
BLAEV0025688	AT1G74960	Encodes a plastidic beta-ketoacyl-ACP synthase II; involved in fatty acid elongation from 16:0-ACP to 18:0-ACP. Homozygous knock-out mutants are embryo lethal, indicating early embryo development is sensitive to elevated 16:0.
BLAEV0004264	AT2G28160	Encodes a putative transcription factor that regulates iron uptake responses. mRNA is detected in the outer cell layers of the root and accumulates in response to iron deficiency. The expression of many iron-regulated genes is dependent on FIT1. It specifically regulates FRO2 at the level of mRNA accumulation and IRT1 at the level of protein accumulation.Similar to FER in tomato and is a regulator of iron uptake. It is post-transcriptionally controlled.
BLAEV0003925	AT2G28640	A member of EXO70 gene family; putative exocyst subunits, conserved in land plants. Arabidopsis thaliana contains 23 putative EXO70 genes, which can be classified into eight clusters on the phylogenetic tree.
BLAEV0006926	AT3G20395	RING/U-box superfamily protein;(source:Araport11)
BLAEV0006925	AT3G20410	calmodulin-domain protein kinase CDPK isoform 9 (CPK9)
BLAEV0006924	AT3G20420	double-stranded RNA binding / ribonuclease III. Required for 3' external transcribed spacer (ETS) cleavage of the pre-rRNA in vivo. Localizes in the nucleus and cytoplasm.
BLAEV0006923	AT3G20430	phosphorylated adapter RNA export-like protein;(source:Araport11)
BLAEV0006922	AT3G20440	Encodes BE1; a putative glycoside hydrolase. Involved in organogenesis and somatic embryogenesis by regulating carbohydrate metabolism. Mutation in BE1 has pleotrophic effect on the whole plant development.
BLAEV0051982	AT3G47570	Leucine-rich repeat protein kinase family protein;(source:Araport11)
BLAEV0038922	AT3G55620	Translation initiation factor IF6;(source:Araport11)
BLAEV0048536	AT4G33080	Ubiquitously expressed protein kinase.
BLAEV0048533	AT4G33110	S-adenosyl-L-methionine-dependent methyltransferases superfamily protein;(source:Araport11)
BLAEV0048532	AT4G33130	Rho GTPase-activating protein;(source:Araport11)
BLAEV0048531	AT4G33140	Haloacid dehalogenase-like hydrolase (HAD) superfamily protein;(source:Araport11)
BLAEV0011503	AT5G16220	Octicosapeptide/Phox/Bem1p family protein;(source:Araport11)
BLAEV0011507	AT5G16230	Encodes one of Δ9 palmitoyl-ACP desaturases responsible for the biosynthesis of ω-7 fatty acids in the maturing endosperm.
BLAEV0011504	AT5G16270	Encodes a SCC1/REC8 ortholog that may be involved in mitosis and may represent a mitotic cohesin. Plays a role in somatic DNA double strand break damage repair. The mRNA is cell-to-cell mobile.
BLAEV0035878	AT5G27970	ARM repeat superfamily protein;(source:Araport11)

BLAEV0016940	AT5G60600	Encodes a chloroplast-localized hydroxy-2-methyl-2-(E)-butenyl 4-diphosphate (HMBPP) synthase (HDS); catalyzes the formation of HMBPP from 2-C-methyl-D-erythrytol 2,4-cyclodiphosphate (MEcPP). The HDS enzyme controls the penultimate steps of the biosynthesis of IPP and dimethylallyl diphosphate (DMAPP) via the MEP pathway and may serve as a metabolic control point for SA-mediated disease resistance. In the light, the electrons required for the reaction catalyzed by HDS are directly provided by the electron flow from photosynthesis via ferredoxin. In the dark however, the enzyme requires an electron shuttle: ferredoxin-$NADP^+$ reductase. The mRNA is cell-to-cell mobile.
BLAEV0016941	AT5G60615	Encodes a defensin-like (DEFL) family protein.
BLAEV0016942	AT5G60620	Glycerol-3-phosphate acyltransferase localized to the ER. Similar to mammalian cells involved in storage oil formation. ER-localized GPAT enzyme responsible for plant membrane lipid and oil biosynthesis.
BLAEV0036288	AT5G61350	Encodes a membrane-localized receptor-like kinase that regulates root hair tip growth by maintaining cytoplasmic Ca2+ gradients. Knockouts of CAP1 produced more cytoplasmic NH4+ and ceased growth of root hairs on MS medium except when NH4+ was depleted; NH4+ depletion reestablished the Ca2+ gradient necessary for normal growth. The lower net NH4+ influx across the vacuolar membrane and relatively alkaline cytosolic pH of root hairs in cap1-1 relative to wild type implied that mutation of CAP1 results in more NH4+ accumulation in the cytoplasm. Furthermore; CAP1 functionally complemented npr1 kinase yeast mutant defective in high-affinity NH4+ uptake via MEP2, distinguishing CAP1 as a cytosolic modulator of NH4+ level that participates in NH4+ homeostasis-regulated root hair growth by modulating tip-focused cytoplasmic Ca2+ gradients.
BLAEV0030923	AT5G63080	Encodes a HR demethylase that acts as a positive regulator of seed germination in the PHYB-PIL5-SOM pathway.
BLAEV0030924	AT5G63090	Involved in lateral organ development
BLAEV0010040	NA	
BLAEV0010042	NA	
BLAEV0011506	NA	
BLAEV0014934	NA	
BLAEV0014935	NA	
BLAEV0016848	NA	
BLAEV0016943	NA	
BLAEV0017008	NA	
BLAEV0028342	NA	
BLAEV0028343	NA	
BLAEV0031561	NA	
BLAEV0035877	NA	
BLAEV0035879	NA	
BLAEV0036282	NA	
BLAEV0036284	NA	
BLAEV0038916	NA	
BLAEV0038918	NA	
BLAEV0038919	NA	
BLAEV0044098	NA	

BLAEV0044127	NA
BLAEV0044758	NA
BLAEV0046193	NA

Table T3. List of meiosis genes highlighted in field populations of diploid-autotetraploid plant species (i.e. *Arabidopsis arenosa* and *A. lyrata*) by previous studies.

Gene	A. thaliana ID	Reference	Analyzed windows	Candidates
AML1	AT5G61960	H12[a]	yes	no
SCC2	AT5G15540	H12	yes	no
SMC3	AT2G27170	H12	yes	no
SMC5	AT5G15920	H12	yes	no
SMC6a	AT5G07660	H12	NA	NA
ASY1	AT1G67370	H12, Y13[b], W15[c], M19[d], B21[e]	yes	no
ASY3	AT2G46980	Y13, W15, M19, B21	yes	no
PDS5b	AT1G77600	Y13, W15, M19, B21	yes	no
PRD3	AT1G01690	Y13, M19, B21	yes	no
SMC3	AT2G27170	Y13	yes	no
SYN1	AT5G05490	Y13, W15, M19, B21	yes	no
ZYP1a	AT1G22260	Y13, W15	yes	no
ZYP1b	AT1G22275	Y13, W15, B21	NA	NA
CYCA2;3	AT1G15570	M19, B21	yes	no
DYAD	AT5G51330	B21	yes	no
SCC3	AT2G47980	B21	yes	no
SCC4	AT5G51340	B21	NA	NA
SDS	AT1G14750	B21	yes	no
SHOC1	AT5G52290	B21	yes	no
SMG7	AT5G19400	B21	yes	no
SMC1	AT3G54670	W15	yes	no

[a] Hollister JD, Arnold BJ, Svedin E, Xue KS, Dilkes BP, Bomblies K. 2012. Genetic adaptation associated with genome-doubling in autotetraploid *Arabidopsis arenosa*. *PLOS Genetics* 8: 1–10.
[b] Yant L, Hollister JD, Wright KM, Arnold BJ, Higgins JD, Franklin FCH, Bomblies K. 2013. Meiotic adaptation to genome duplication in *Arabidopsis arenosa*. *Current Biology* 23: 2151–2156.
[c] Wright KM, Arnold B, Xue K, Surinova M, O'Connell J, Bomblies K. 2015. Selection on meiosis genes in diploid and tetraploid *Arabidopsis arenosa*. *Molecular Biology and Evolution* 32: 944–955.
[d] Marburger S, Monnahan P, Seear PJ, Martin SH, Koch J, Paajanen P, Bohutínská M, Higgins JD, Schmickl R, Yant L. 2019. Interspecific introgression mediates adaptation to whole genome duplication. *Nature communications* 10: 5218.
[e] Bohutínská M, Alston M, Monnahan P, Mandáková T, Bray S, Paajanen P, Kolář F, Yant L. 2021. Novelty and convergence in adaptation to whole genome duplication. *Molecular Biology and Evolution* 38: 3910–3924.

Table T4. List of meiosis-related genes compiled from TAIR10 by Yant *et al.* (2013)[a] and whether the *B. laevigata* ortholog is encompassed within analyzed windows (AW) and in the set of candidate genes (CG)

AT_ID	Name	Description	BL_ID	AW	CG
AT1G01690	PRD3	Novel plant-specific protein involved in meiotic double strand break formation.	BLAEV0051826	yes	no
AT1G06660	JASON	Mutant produces diploid male gametes	BLAEV0051366	yes	no
AT1G08880	H2AX		BLAEV0009509	yes	no
AT1G10710	PHS1	regulates recombination and pairing	NA	NA	NA
AT1G10930	RECQ4A	DNA damage response, suppression of homologous recombination.	BLAEV0009766	yes	no
AT1G22260	ZYP1a	SC transverse filament protein	BLAEV0022206	yes	no
AT1G22275	ZYP1b	SC transverse filament protein	NA	NA	NA
AT1G27900			BLAEV0034447	yes	no
AT1G34355	PS1	Mutant produces 2N pollen (Meiosis II defect)	BLAEV0033328	yes	no
AT1G35530	FANCM	helicase that functions as a major factor limiting meiotic crossover formation.	BLAEV0033284	yes	no
AT1G53490	HEI10	Belongs to a group of ZMM proteins well conserved among species	BLAEV0023217	yes	no
AT1G54690	HTA3	(No data - Poorly aligned in A. arenosa) Encodes HTA3, a histone H2A.	NA	NA	NA
AT1G60930	RECQ4B	Involved in homologous recombination	NA	NA	NA
AT1G63990	AtSPO11-2	DSB induction, Required for meiotic recombination.	BLAEV0021833	yes	no
AT1G66170	MMD1	DNA binding, male meiosis, microsporogenesis	BLAEV0024449	yes	no
AT1G67370	ASY1	Chiasma assembly	BLAEV0022789	yes	no
AT1G77320	MEI1	Mutant defective in meiosis and produces abnormal microspores.	BLAEV0022098	yes	no
AT1G77390	CYCA1;2	core cell cycle gene involved in meiosis II during microsporogenesis.	BLAEV0025776	yes	no
AT1G77600		Homologous to animal sister chromatid cohesion protein PDS5	BLAEV0025767	yes	no
AT2G06510	RPA1A	(No data - Poorly aligned in A. arenosa), meiosis I in pollen mother cells.	BLAEV0031392	yes	no
AT2G27170	SMC3	Member of the cohesin complex, partner of SMC1	BLAEV0004140	yes	no
AT2G31970	RAD50	Involved in double strand break repair.	BLAEV0003585	yes	no
AT2G45280	ATRAD51C	(No data - Poorly aligned in A. arenosa) RAD51 homolog, DNA repair	BLAEV0005029	yes	no
AT2G46980	ASY3	Encodes ASY3, a coiled-coil domain protein required for normal meiosis.	BLAEV0001797	yes	no
AT2G47980	SCC3	Essential to the monopolar orientation of the kinetochores during meiosis.	BLAEV0001725	yes	no
AT3G02680	NBS1	DNA repair and meiotic recombination protein	BLAEV0008545	yes	no
AT3G02980	MCC1	required in meiosis for normal chiasma number and distribution	NA	NA	NA
AT3G05480	RAD9	DNA repair and homologous recombination	BLAEV0008218	yes	no
AT3G13170	AtSPO11-1	DSB induction, Required for meiotic recombination.	BLAEV0026944	yes	no
AT3G18524	MSH2	MutS-homolog family, required for normal levels of recombination in yeast	BLAEV0007220	yes	no

AT3G19210	ATRAD54	RAD54, member of SWI2/SNF2 family. DNA repair via HR	BLAEV0007143	yes	no
AT3G20475	MSH5	MutS-homolog family, required for normal levels of recombination in yeast	BLAEV0031919	yes	no
AT3G22880	DMC1	(No data - Poorly aligned in A. arenosa) Chiasma assembly / DNA repair	BLAEV0010499	yes	no
AT3G25100	CDC45	Required for normal meiosis	BLAEV0031508	yes	no
AT3G43210	TETRASPORE	Required for cytokinesis in pollen.	BLAEV0040229	yes	no
AT3G48190	ATM	homolog of human ataxia telangiectasia mutated	BLAEV0039647	yes	no
AT3G52115	AtCOM1	Sae2 like, DNA repair	BLAEV0018754	yes	no
AT3G54670	SMC1	Structural maintenance of chromosomes family; member of cohesin complex.	BLAEV0032319	yes	no
AT3G57300	INO80	SWI/SNF ATPase. positive regulator of homologous recombination (HR).	BLAEV0032654	yes	no
AT4G00020	BRCA2A	Ortholog of breast cancer susceptibility protein 2. Essential at meiosis.	NA	NA	NA
AT4G02070	MSH6	Homolog of MSH6	BLAEV0000268	yes	no
AT4G09140	MLH1	Encodes a protein with similarity to Mut1 DNA mismatch repair protein.	BLAEV0001066	yes	no
AT4G14220	RHF1A	encodes a RING-type E3 ubiquitin ligase implicated in gametogenesis.	BLAEV0038577	yes	no
AT4G17380	MSH4	homolog of MSH4, meiosis-specific member of MutS-homolog family of genes.	BLAEV0043184	yes	no
AT4G20900	MS5	In ms5 mutants, pollen tetrads have an extra round of division after meiosis II	BLAEV0036797	yes	no
AT4G21270	ATK1	Encodes a kinesin-like motor protein heavy chain involved in spindle formation	BLAEV0046646	yes	no
AT4G22970	ESP	Encodes a separase (ESP), homologous to human / mouse separase protein.	BLAEV0037074	yes	no
AT4G25540	MSH3	DNA mismatch repair	BLAEV0049499	yes	no
AT4G29170	ATMND1	might play a role in DSB repair during meiosis.	BLAEV0049049	yes	no
AT4G30870	MUS81	Associates with noninterfering crossovers	BLAEV0045631	no	no
AT4G32200	ASY2	homologue of ASY1, redundant	NA	NA	NA
AT5G01630	BRCA2B	Ortholog of breast cancer susceptibility protein 2. Essential at meiosis.	NA	NA	NA
AT5G05490	SYN1	Encodes a RAD21-like gene essential for meiosis.	BLAEV0019201	yes	no
AT5G07660	SMC6a	SMC5/6 complex; sister chromatid alignment and homologous recombination	NA	NA	NA
AT5G15540	SCC2	Encodes Adherin SCC2. Sister chromatid cohesion	BLAEV0020099	yes	no
AT5G15920	SMC5	SMC5/6 complex; sister chromatid alignment and homologous recombination	BLAEV0020051	yes	no
AT5G16270	SYN4	Rad21 homolog	BLAEV0011504	yes	yes
AT5G19400	SMG7	Required for exit from meiosis.	BLAEV0017364	yes	no
AT5G22000	RHF2A	allows mitoses associated with megagametogenesis and microgametogenesis.	BLAEV0011120	yes	no
AT5G24280	GMI1	Involved in somatic homologous recombination.	BLAEV0030399	yes	no
AT5G40820	ATR	Encodes a Arabidopsis ortholog of the ATR protein kinase that is involved in a wide range of responses to DNA damage and plays a central role in cell-cycle regulation.	BLAEV0025309	yes	no

AT5G47690	PDS5-like	Homologous to animal sister chromatid cohesion protein PDS5	BLAEV0038259	yes	no
AT5G48390	ATSPO22	Involved in crossover formation.	BLAEV0044515	yes	no
AT5G48720	XRI1	resolution of meiotic recombination intermediates	NA	NA	NA
AT5G51330	DYAD/SWI1	Involved in sister chromatid cohesion and meiotic chromosome organization	BLAEV0015623	yes	no
AT5G52290	SHOC1	Mutants have defects in meiosis.	BLAEV0015759	yes	no
AT5G54260	MRE11	DNA repair / meiotic recombination protein, component of MRE11 complex	BLAEV0016021	yes	no
AT5G57450	XRCC3	homologous recombination / repair.	BLAEV0016487	yes	no
AT5G61460	SMC6b	SMC5/6 complex; sister chromatid alignment and homologous recombination	BLAEV0036304	yes	no
AT5G63920	TOP3a	Encodes topoisomerase 3alpha	BLAEV0031017	yes	no
AT5G66130	RAD17	regulation of DNA damage repair and homologous recombination	BLAEV0035597	yes	no

[a]Yant L, Hollister JD, Wright KM, Arnold BJ, Higgins JD, Franklin FCH, Bomblies K. 2013. Meiotic adaptation to genome duplication in *Arabidopsis arenosa*. *Current Biology* 23: 2151–2156.

Table T5. Gene ontology terms significantly enriched in candidate genes underlying adaptation to whole-genome duplication in *Biscutella laevigata*. Only terms supported by three or more significant genes were considered.

**Term**	**Annotated**	**Significant**	**Expected**	**topgoFisher**
jasmonic acid biosynthetic process	120	3	0.16	0.00060
sulfur amino acid metabolic process	352	3	0.48	0.00147
amino acid biosynthetic process	648	4	0.88	0.00372
fatty acid biosynthetic process	442	6	0.6	0.00561
regulation of plant-type hypersensitive response	305	3	0.42	0.00833
protein targeting to membrane	353	3	0.48	0.01235
biosynthetic process	14815	26	20.16	0.01288
response to ethylene	390	3	0.53	0.03638
defense response to fungus	548	3	0.75	0.03860

References

Beaulieu JM, Leitch IJ, Patel S, Pendharkar A, Knight CA. 2008. Genome size is a strong predictor of cell size and stomatal density in angiosperms. *New Phytologist* 179:975–986.

Bohutínská M, Alston M, Monnahan P, Mandáková T, Bray S, Paajanen P, Kolář F, Yant L. 2021. Novelty and convergence in adaptation to whole genome duplication. *Molecular Biology and Evolution* 38:3910–3924.

Bomblies K, Jones G, Franklin C, Zickler D, Kleckner N. 2016. The challenge of evolving stable polyploidy: could an increase in “crossover interference distance” play a central role? *Chromosoma* 125:287–300.

Bomblies K, Madlung A. 2014. Polyploidy in the *Arabidopsis* genus. *Chromosome Research* 22:117–134.

Bray SM, Hämälä T, Zhou M, Busoms S, Fischer S, Desjardins SD, Mandáková T, Moore C, Mathers TC, Cowan L, et al. 2024. Kinetochore and ionomic adaptation to whole-genome duplication in *Cochlearia* shows evolutionary convergence in three autopolyploids. *Cell Reports* 43:114576.

Cavalier-Smith T. 2005. Economy, speed and size matter: evolutionary forces driving nuclear genome miniaturization and expansion. *Annals of Botany* 95:147–175.

Clo J, Kolář F. 2021. Short- and long-term consequences of genome doubling: a meta-analysis. *American Journal of Botany* 108:2315–2322.

Comai L. 2005. The advantages and disadvantages of being polyploid. *Nature reviews. Genetics* 6:836–846.

Gonzalo A, Parra-Nunez P, Bachmann AL, Sanchez-Moran E, Bomblies K. 2023. Partial cytological diploidization of neoautotetraploid meiosis by induced cross-over rate reduction. *Proceedings of the National Academy of Sciences* 120:e2305002120.

Hollister JD, Arnold BJ, Svedin E, Xue KS, Dilkes BP, Bomblies K. 2012. Genetic adaptation associated with genome-doubling in autotetraploid *Arabidopsis arenosa*. *PLOS Genetics* 8:1–10.

Marburger S, Monnahan P, Seear PJ, Martin SH, Koch J, Paajanen P, Bohutínská M, Higgins JD, Schmickl R, Yant L. 2019. Interspecific introgression mediates adaptation to whole genome duplication. *Nature communications* 10:5218.

Morgan C, White MA, Franklin FCH, Zickler D, Kleckner N, Bomblies K. 2021. Evolution of crossover interference enables stable autopolyploidy by ensuring pairwise partner connections in *Arabidopsis arenosa*. *Current Biology* 31:4713-4726.e4.

Ramsey J, Schemske DW. 2002. Neopolyploidy in flowering plants. *Annual Review of Ecology and Systematics* 33:589–639.

Wright KM, Arnold B, Xue K, Surinova M, O’Connell J, Bomblies K. 2015. Selection on meiosis genes in diploid and tetraploid *Arabidopsis arenosa*. *Molecular Biology and Evolution* 32:944–955.

Yant L, Hollister JD, Wright KM, Arnold BJ, Higgins JD, Franklin FCH, Bomblies K. 2013. Meiotic adaptation to genome duplication in *Arabidopsis arenosa*. *Current Biology* 23:2151–2156.
